# NAD(H) and NADP(H) in plants and mammals

**DOI:** 10.1016/j.molp.2025.05.004

**Published:** 2025-06-02

**Authors:** Danying Lu, Murray Grant, Boon Leong Lim

**Affiliations:** 1 School of Biological Sciences, University of Hong Kong, Pokfulam, Hong Kong, China; 2 School of Life Sciences, University of Warwick, Gibbet Hill Campus, Coventry, UK; 3 State Key Laboratory of Agrobiotechnology, The Chinese University of Hong Kong, Shatin, Hong Kong, China

**Keywords:** chloroplasts, mitochondria, pyridine nucleotides, redox

## Abstract

Nicotinamide adenine dinucleotide (NAD) and nicotinamide adenine dinucleotide phosphate (NADP) are essential metabolic coenzymes in prokaryotic and eukaryotic cells, with their reduced forms, NAD(P)H, serving as electron donors for myriad reactions. NADH is mainly involved in catabolic reactions, whereas NADPH is mainly involved in anabolic and antioxidative reactions. The presence of endosymbiont-derived organelles in eukaryotes has made the functional division of NADH and NADPH systems more complex. Chloroplasts in photoautotrophic eukaryotes provide additional sources of reductants, complicating the maintenance of the redox balance of NAD(P)^+^/NAD(P)H compared with heterotrophic eukaryotes. In this review, we discuss the two redox systems in plants and systematically compare them with those in mammals, including the similarities and differences in the biosynthesis and subcellular transport of NAD^+^, the biosynthesis of NADP^+^, and metabolic reactions for the reduction and oxidation of NAD(P)H. We also review the regulation of pyridine nucleotide pools and their ratios in different plant subcellular compartments and the effects of light on these ratios. We discuss the advantages of having both NADH and NADPH systems, highlight current gaps in our understanding of NAD(P)H metabolism, and propose research approaches that could fill in those gaps. The knowledge about NADH and NADPH systems could be used to guide bioengineering strategies to optimize redox-regulated processes and improve energy-use efficiency in crop plants.

## Teaser

This review comprehensively compares the NADH/NAD^+^ and NADPH/NADP^+^ redox systems in plants and mammals. It highlights reasons for the coexistence of NADH and NADPH systems, points out key gaps in our understanding of pyridine nucleotide metabolism, and proposes future research directions to address these questions.

## Introduction

The oxidized and reduced forms of nicotinamide adenine dinucleotide (NAD^+^/NADH) and nicotinamide adenine dinucleotide phosphate (NADP^+^/NADPH) play essential roles in energy metabolism in all eukaryotes and prokaryotes. NAD^+^ and NADP^+^ “pick up” electrons and protons to generate NADH and NADPH, respectively. These reduced molecules serve as electron donors that drive hundreds of redox reactions in living organisms. Adenosine triphosphate (ATP), the energy currency of many biological activities, is generated via the breakdown of carbon molecules through glycolysis and oxidative phosphorylation. During oxidative phosphorylation, electrons are transferred from NADH to oxygen (Tourmente et al., 2015; Wilson, 2017; Zhang et al., 2020a). Therefore, NADH is a key molecule that supplies reducing power to many catabolic pathways. NADPH also serves as a key electron donor and is mainly involved in anabolic pathways and antioxidative reactions (Chandel, 2021).

In this review, we describe how the two different redox coupling systems (NAD^+^/NADH and NADP^+^/NADPH) are deployed in plants and other organisms and discuss the importance of having both redox systems. Moreover, we compare heterotrophic eukaryotes, which acquired mitochondria as their major powerhouses from one endosymbiotic event, with photoautotrophic eukaryotes, which acquired chloroplasts and mitochondria from two distinct endosymbiotic events (Bjorn and Govindjee, 2009). Comparing mammals and plants provides insights into the diversity of redox coupling across eukaryotes.

## NAD^+^ biosynthesis

**Figure 1:**
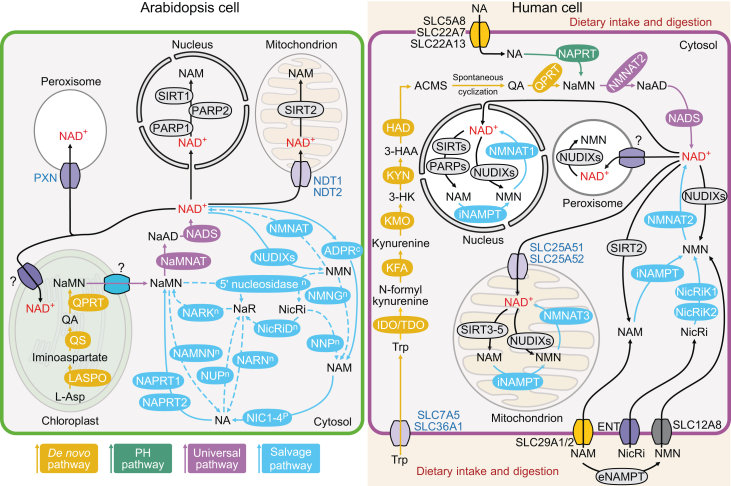
NAD^+^ biosynthetic pathways in *Arabidopsis* and human cells. Plants use the *de novo* pathway and the salvage pathway to synthesize NAD^+^. In the *de novo* pathway, plants use Asp as the precursor to generate QA in plastids through a two-step enzymatic reaction catalyzed by LASPO and QS; QA is converted to NaMN by QPRT. NaMN is transported into the cytosol and used as a substrate to generate NaAD by NaMNAT. Finally, NaAD is converted to NAD^+^ through NADS-mediated catalysis. The salvage pathway of plants primarily occurs in the cytosol and can be divided into two major recycling routes: via NicRi or via NAM. Cytosolic NAM can be generated from NAD^+^ in two steps via NUDIXs and NMNG or directly from NAD^+^ via ADPRc. NMN produced by NUDIX-catalyzed NAD^+^ degradation may be directly converted back to NAD^+^ via NMNAT. In addition, NicRi can generate NAM via NNP^n^. NAM is then converted to NA via NIC1-4^P^, and the resulting NA is further converted to NaMN by NAPRT1 and NAPRT2. Subsequently, NaMN enters the universal pathway to generate NAD^+^. NaMN can be converted back to NA via NAMNN^n^. NicRi is generated through NMN degradation via 5′ nucleosidases. NicRi is then converted to NaR via NicRiD^n^. NaR is converted to NaMN through NaRK and enters the universal pathway to produce NAD^+^. NaMN can be converted back to NaR via 5′ nucleosidases. NaR and NA can be interconverted via NARN^n^ and NUP^n^, respectively. NAD^+^ can be transported into mitochondria via the transporters NDT1 and NDT2 and degraded into NAM via SIRT2. NAD^+^ can also enter the nucleus through the nuclear pore complex, where it will be degraded into NAM through the nucleus-localized NAD^+^-consuming enzymes SIRT1, PARP1, and PARP2. Human cells synthesize NAD^+^ through three pathways: the *de novo* pathway, the salvage pathway, and the Preiss–Handler (PH) pathway. The human *de novo* pathway occurs strictly in the cytosol. Extracellular Trp is transported into the cytosol through SLC7A5 and SLC36A1 and is subsequently converted to *N*-formylkynurenine via IDO or TDO, whose product is converted to kynurenine by KFA. Kynurenine is converted to 3-HK by KMO, followed by the conversion of 3-HK to 3-HAA via HYN. 3-HAA is further converted to ACMS via HAD, after which ACMS is converted to QA through spontaneous cyclization. The resulting QA is converted to NaMN by QPRT, and NaMN is then converted to NaAD by NMNAT2. Finally, NaAD is converted to NAD^+^ by NADS. In the PH pathway, extracellular NA enters the cytosol through SLC5A8, SLC22A7, and SLC22A13 before being converted to NaMN via NAPRT. NaMN then enters the universal pathway to generate NAD^+^. The human salvage pathway takes place in the cytosol, nucleus, and mitochondria. In the cytosolic salvage pathway, extracellular NAM and NicRi are transported into the cytosol by SLC29A1/2 and ENT, respectively. Cytosolic NAM and NicRi are subsequently converted to NMN via iNAMPT and via NicRiK1 or NicRiK2, respectively. Extracellular NAM can also be converted to NMN by eNAMPT before entering the cytosol via SLC12A8. Cytosolic NMN is further converted to NAD^+^ by NMNAT2. Cytosolic NAD^+^ can be degraded to NAM by NAD-consuming enzymes (such as SIRT2) or to NMN via NUDIXs, the products of which can enter the recycling pathway. In addition, cytosolic NAD^+^, NAM, and NMN can enter the nucleus through the nuclear pore complex. In the nucleus, NAD^+^ can be degraded to NAM by SIRTs and PARPs, and the resulting NAM can be converted to NMN by iNAMPT. NMN can also be obtained from NAD degradation via nucleus-localized NUDIXs. NMN is ultimately converted to NAD^+^ by NMNAT1. Cytosolic NAD^+^ can also enter mitochondria through SLC25A51 and SLC25A52 to be converted to NAM via SIRT3–5. NAM is then converted to NMN by mitochondrial iNAMPT, and NMN is converted to NAD^+^ by NMNAT3. Mitochondrial NAD can also be degraded into NMN by NUDIX. In mammalian cells, NUDIX is also present in peroxisomes; it is thought that cytosolic NAD^+^ can enter peroxisomes through unknown transporters. ACMS, 2-amino-3-carboxy-muconate-semialdehyde; ADPRc, ADP-ribose cyclase; Asp, aspartate; ENT, equilibrative nucleoside transporters; eNAMPT, extracellular nicotinamide phosphoribosyl transferase; HAD, 3-hydroxyanthranilic acid 3,4-dioxygenase; 3-HK, 3-hydroxy-kynurenine; 3-HAA, 3-hydroxyanthranilic acid; IDO, indoleamine 2,3-dioxygenase; iNAMPT, intracellular nicotinamide phosphoribosyl transferase; KFA, kynurenine formamidase; KMO, kynurenine-3-monooxygenase; KYN, kynureninase; LASPO, L-aspartate oxidase; NAM, nicotinamide; NADS, NAD synthetase; NaAD, nicotinate adenine dinucleotide; Na/NMNAT, nicotinate mononucleotide/nicotinamide mononucleotide adenylyltransferase; NaRK, nicotinate ribose kinase; NARN, nicotinate ribose nucleoside; NaR, nicotinate ribose; NAPRT, nicotinate phosphoribosyltransferase; NAMNN, nicotinate mononucleotide nucleosidase; NaMN, nicotinate mononucleotide; NA, nicotinic acid; NDT, NAD^+^ transporters; NIC, nicotinamidase; NicRiD, nicotinamide riboside deaminase; NicRi, nicotinamide ribose; NUDIX, NUDIX hydrolases; NuP, nucleoside phosphorylase; NMN, nicotinamide mononucleotide; NMNG, nicotinamide mononucleotide glycohydrolase; NNP, nicotinate nucleoside pyrophosphatase; NicRiK, nicotinamide riboside kinase; PARP, poly(ADP-ribose) polymerases; PXN, peroxisomal NAD carrier; QA, quinolinic acid; QPRT, quinolate phosphoribosyltransferase; QS, quinolinate synthase; SLC, soluble carrier; SIRT, sirtuin; Trp, tryptophan; TDO, tryptophan 2,3-dioxygenase. Superscript “P” indicates that the subcellular localizations of these enzymes were predicted by MULocDeep (https://mu-loc.org/). Superscript “n” indicates that the enzymes and their encoding genes have not yet been identified in *Arabidopsis*, although their activities have been identified in other plants.

All living organisms require NAD(P)H to support their life activities. However, not all organisms synthesize NAD^+^ or NADP^+^ [NAD(P)^+^ hereafter]. Indeed, several prokaryotes, such as *Haemophilus influenzae* and *Chlamydia trachomatis*, have lost the biosynthetic enzymes necessary for NAD(P)^+^ biosynthesis and thus rely on the uptake of NAD(P)^+^ from their hosts (Reidl et al., 2000; Fisher et al., 2014). Other prokaryotes generate NAD^+^ via three pathways: the *de novo* pathway, the salvage pathway, and the Preiss–Handler (PH) pathway (Supplemental Figure 1). Any given prokaryotic species may contain one or more of these pathways (Kurnasov et al., 2003). Similar to prokaryotes, mammals utilize the *de novo* pathway, the salvage pathway, and the PH pathway to synthesize NAD^+^. By contrast, vascular plants only possess the *de novo* and salvage/recycling pathways, as, unlike mammals, plants effectively synthesize nicotinic acid (NA) (Hunt et al., 2004; Xiao et al., 2018) (Figure 1).

### The *de novo* pathway

In the *de novo* pathway (Figure 1), NAD^+^ is generated from quinolinic acid (QA), which is converted to nicotinate mononucleotide (NaMN) by quinolate phosphoribosyltransferase. NaMN is then adenylated to form nicotinate adenine dinucleotide (NaAD) by nicotinate mononucleotide/nicotinamide mononucleotide adenylyltransferase (NaMNAT/NMNAT), a bifunctional enzyme that also converts NAD^+^ to nicotinamide mononucleotide (NMN) in the salvage pathway. Finally, NaAD is amidated to form NAD^+^ via NAD synthetase. This biosynthetic pathway from QA to NAD^+^ is highly conserved among prokaryotes, plants, and mammals (Rodionov et al., 2008; Gakière et al., 2018; Covarrubias et al., 2021). L-aspartate (Asp) and L-tryptophan (Trp) both serve as precursors for QA biosynthesis. The biosynthesis of QA from Asp is accomplished via a two-step reaction catalyzed by L-aspartate oxidase (LASPO) and quinolinate synthase (QS) (Katoh et al., 2006; Spaans et al., 2015). In the Trp-to-QA pathway, Trp is converted to 2-amino-3-carboxy-muconate-semialdehyde through a five-step enzymatic reaction, which then spontaneously cyclizes to QA (Figure 1) (Magni et al., 2008; Badawy, 2017).

Prokaryotes use Asp or Trp as a precursor for QA biosynthesis, but plants only utilize Asp, whereas mammals only utilize Trp (Kurnasov et al., 2003; Gakière et al., 2018; Xiao et al., 2018). Most prokaryotes, including *Escherichia coli*, *Bacillus subtilis*, and cyanobacteria, use Asp as a precursor for *de novo* NAD^+^ biosynthesis, while some prokaryotes, such as *Xanthomonas pruni*, use Trp (Kurnasov et al., 2003; Gerdes et al., 2006; Lima et al., 2009). Cyanobacteria, a division of prokaryotes that perform photosynthesis and are thought to be the evolutionary ancestors of plant chloroplasts, solely utilize Asp for *de novo* NAD^+^ biosynthesis, a pathway that might have been retained throughout evolution from cyanobacteria to vascular plants (Gerdes et al., 2006; Ishikawa and Kawai, 2019).

### Differences in precursor choices in the *de novo* pathway in plants and mammals

Plants synthesize all 20 standard amino acids, whereas mammals synthesize only 11, including Asp. Therefore, mammals must obtain the nine remaining essential amino acids, including Trp, from their diet (Trovato et al., 2021) (Figure 1). Thus, it is worth reflecting on why plants might have evolved the ability to use Asp, whereas mammals evolved the ability to use Trp for their respective *de novo* NAD^+^ biosynthesis pathways.

Efficiency and economy are important reasons for plants to use Asp rather than Trp for *de novo* NAD^+^ biosynthesis. In plants, Trp biosynthesis begins with the shikimic acid pathway in plastids, which comprises a series of enzymatic reactions that require large amounts of energy. Furthermore, Trp is one of the least abundant amino acids in plants, with low abundance in solution (1–15 μM) within plant cells (Last, 1995). Compared to the complex and energetically costly Trp biosynthetic process, Asp biosynthesis is much more economical (Akashi and Gojobori, 2002). Aspartate transaminase (AspAT) directly converts glutamate (Glu) and oxaloacetate (OAA) to 2-oxoglutarate (2-OG) and Asp. In plants, Glu is *de novo* synthesized via nitrate assimilation, with the final step occurring in chloroplasts or plastids via the glutamine synthetase (GS)–glutamate synthase (GOGAT) cycle (Dragicevic et al., 2016). As a result, Glu is abundant in chloroplasts, making these organelles a good location for Asp production. It is therefore easy to understand why QA biosynthesis occurs in chloroplasts and why Asp was selected as the precursor for *de novo* NAD^+^ biosynthesis during evolution: Asp biosynthesis is more energetically efficient than Trp biosynthesis.

In mammalian cells, *de novo* Asp biosynthesis primarily occurs through the transamination of OAA via AspAT (Ndrepepa, 2021). Asp is also obtained directly from the diet, as the amount of Asp derived from food is markedly higher than the amount of Trp (Gardner et al., 2019). In humans, Trp from the diet is transported into the cytosol via the solute carrier family members SLC7A5 and SLC36A1 (Larsen et al., 2009; Jiang et al., 2024). Nevertheless, it is surprising that mammals have lost the enzymes LASPO and QS, preventing them from using Asp as a precursor for *de novo* NAD^+^ biosynthesis (Bossi et al., 2002; Gerdes et al., 2002). The computer-calculated values of the change in Gibbs free energy (ΔG) for the reactions catalyzed by LASPO and QS are −53.2 and −24.0 kcal/mol, respectively, indicating that these two reactions are highly favorable and irreversible (https://biocyc.org/). Accordingly, we suggest that, if these two enzymes were retained in mammals during evolution, most of the Asp obtained from the diet would be consumed for *de novo* NAD^+^ biosynthesis, which would deplete the Asp pool available for protein synthesis. This may explain why mammals adopted the Trp pathway rather than the Asp pathway for *de novo* NAD^+^ biosynthesis during their evolution. Notably, most mammalian cells do not express the genes encoding the enzymes necessary for the Trp-to-QA conversion pathway and rely on circulating nicotinamide (NAM) to NAD^+^ via the salvage pathway (Liu et al., 2018; Covarrubias et al., 2021). This preference for the salvage pathway is attributed to its energetic efficiency compared with the high energy cost of *de novo* NAD^+^ biosynthesis via Trp.

### The salvage/recycling pathway

Following NAD^+^ biosynthesis via the *de novo* pathway, NAD^+^ is degraded to NAM by various NAD^+^-consuming enzymes, including NAD^+^ glycohydrolases, polyADP-ribose polymerases (PARPs), and sirtuins (SIRTs). NAM itself is subsequently degraded to other by-products (Figure 1). In addition, NAD^+^ is directly converted to NMN by NUDIX hydrolases (NUDIXs). The products of these reactions are recycled through the salvage pathway to regenerate NAD^+^ (Hunt et al., 2004; Braidy et al., 2019; Kulikova and Nikiforov, 2020). In general, salvage pathways recycle NAD^+^ enzymatically via NAM, nicotinamide ribose (NicRi), or NMN. Another NAD^+^ recycling pathway, the PH pathway, uses NA as a substrate (Figure 1) (Preiss and Handler, 1958; Noctor et al., 2011). Precursors of the salvage and PH pathways in prokaryotes and mammals are obtained from the environment and diet, respectively, and through the recycling of NAD^+^ degradation products. Most prokaryotes recycle NAD^+^ using the NA, NicRi, or NAM pathway or two of these pathways (Supplemental Figure 1) (Gerdes et al., 2006; Rodionov et al., 2008), whereas mammals contain both a complete PH pathway and a salvage pathway (Figure 1). Due to the compartmentalization of mammalian cells, the NAD^+^ recycling pathway is much more complex in mammals than in prokaryotes.

In humans, extracellular NA enters the cytosol via SLC5A8, SLC22A7, and SLC22A13 (Ganapathy et al., 2005; Bahn et al., 2008; Mathialagan et al., 2020). Cytosolic NA is subsequently converted to NaMN via nicotinate phosphoribosyltransferase (NAPRT), which then enters the universal pathway to regenerate NAD^+^ (Figure 1) (Fricker et al., 2018; Mehmel et al., 2020; Chini et al., 2021). The human salvage pathway is active in the cytosol, mitochondria, and nucleus (Figure 1). In these compartments, NAD^+^ is degraded into NAM and NMN by NAD^+^-consuming enzymes and NUDIXs, respectively. NAM is then converted to NMN by intracellular nicotinamide phosphoribosyltransferase (NAMPT) in these compartments, and NMN is ultimately regenerated to form NAD^+^ via the corresponding NMNAT (Berger et al., 2005; Michishita et al., 2005; Boehler et al., 2011; Bai and Canto, 2012; Kulikova and Nikiforov, 2020; Covarrubias et al., 2021). Mammals also contain extracellular NAMPT. This enzyme converts extracellular NAM to NMN, which enters the cytosol via the importer SLC12A8 (Travelli et al., 2018; Grozio et al., 2019). The extracellular NAM and NicRi are imported into cells through SLC29A1-2 and equilibrative nucleoside transporters, respectively (Kropotov et al., 2021; Chen et al., 2025).

Notably, the NAD^+^ recycling pathway in plants differs greatly from those in animals and prokaryotes, as the precursors are all derived from NAD^+^ degradation and the NAD^+^ recycling pathway itself (Figure 1). In plants, the salvage pathway, which primarily functions in the cytosol, is divided into two recycling routes based on the source of precursors (NicRi or NAM). In the NAM recycling branch, NAD^+^ is first degraded to NMN by cytosolic NUDIXs and is then converted to NAM via NMN glycohydrolase (NMNG) (Wagner et al., 1986; Yoshimura and Shigeoka, 2015). NAM is also generated from NAD^+^ via ADP-ribose cyclase present at the plasma membrane (Sanchez et al., 2004). Subsequently, NAM generates NA via cytosolic nicotinamidase (NIC), and NA generates NaMN via NAPRTs before entering the universal pathway to regenerate NAD^+^ (Gakière et al., 2018; Ahmad et al., 2021; Jiang et al., 2021). NaMN is also converted back to NA via NaMN nucleosidase (NAMNN) (Wagner et al., 1986). In the NicRi recycling branch, NMN is converted to NicRi by 5′ nucleosidase, and NaMN is then generated through a two-step reaction via NicRi deaminase (NicRiD) and nicotinate ribose kinase (NaRK), ultimately regenerating NAD^+^. Notably, in this branch of the recycling pathway, NaMN is converted back to nicotinate ribose (NaR) by 5′ nucleosidase.

The intermediate products of these two recycling pathways are interconvertible, facilitating the flexible adjustment of the NAD^+^ pool (Figure 1). NicRi is converted to NAM via nicotinate nucleoside pyrophosphatase (NNP). NaR is transformed to NA via NaR nucleoside (NARN), and the reverse reaction is catalyzed by nucleoside phosphorylase (NuP) (Matsui and Ashihara, 2008; Katahira and Ashihara, 2009; Ashihara and Deng, 2012). The plant salvage pathway is less well characterized. The presence, function, and encoding genes of NaRK, 5′ nucleosidase, NAMNN, NARN, NuP, NNP, NMNG, and NicRiD in Arabidopsis (*Arabidopsis thaliana*) are still unclear (Gakière et al., 2018). The enzyme activities of NaRK, 5′ nucleosidase, NARN, and NNP were detected in both potato (*Solanum tuberosum*) tubers and tea plant (*Camellia sinensis*) (Katahira and Ashihara, 2009; Ashihara and Deng, 2012). The enzyme activities of NAMNN and NMNG were detected in tobacco (*Nicotiana tabacum*) (Wagner et al., 1986). Furthermore, the enzyme activity of NuP was detected in mung bean (*Vigna radiata*) (Matsui and Ashihara, 2008), and the enzyme activity of NicRiD was detected in potato (Katahira and Ashihara, 2009).

As in mammals, NAD^+^ synthesized in the plant cytosol is transported to different organelles and degraded (Figure 1). In Arabidopsis, mitochondrial NAD^+^ is degraded to NAM via SIRT2, and NAD^+^ in the nucleus is degraded to NAM through SIRT1, and PARP1-2 (Konig et al., 2014; Song et al., 2015; Liu et al., 2017). Arabidopsis NAD-metabolizing NUDIXs are only found in the cytosol, and the subcellular localizations and characteristics of several Arabidopsis NUDIXs still need to be verified (Yoshimura and Shigeoka, 2015). NMNAT activity was detected in the mitochondria of *Helianthus tuberosus* tubers, suggesting it may regenerate NAD^+^ from NMN in mitochondria (Martino and Pallotta, 2011). The Arabidopsis genome contains only one gene encoding Na/NMNAT, which prefers NaMN, but it is unclear whether it encodes a mitochondrial isoform (Hashida et al., 2007). Therefore, evidence to support the existence of NAD^+^ recycling pathways in the mitochondria, nucleus, or peroxisomes of Arabidopsis is not yet available (Figure 1).

## NAD^+^ transport

In eukaryotic cells, the final step of *de novo* NAD^+^ biosynthesis takes place only in the cytoplasm (Figure 1). Therefore, NAD^+^ must be transported into organelles for specific metabolic pathways following its biosynthesis. The nuclear membrane is freely permeable to NAD^+^ through nuclear pores (Fjeld et al., 2003), and cytosolic NAD^+^ is also transported to multiple organelles via NAD^+^ transporters. In Arabidopsis, three NAD^+^ transporters—AtNDT1, AtNDT2, and PEROXISOMAL NAD^+^ CARRIER (AtPXN)—have been identified, with AtNDT1 and AtNDT2 both localized to mitochondria (Douce and Neuburger, 1989; Chaves et al., 2019; Luo et al., 2019). Although early studies indicated that AtNDT1 localizes to the inner chloroplast membrane, more recent subcellular localization assays indicated that AtNDT1 is instead specifically present in the inner mitochondrial membrane (Palmieri et al., 2009; Chaves et al., 2019). Therefore, exactly how plant plastids acquire NAD^+^ from the cytosol remains to be elucidated. Early *in vitro* studies suggested that the peroxisomal NAD^+^ transporter AtPXN could transport NAD(H) and coenzyme A (CoA) (Agrimi et al., 2012b; Bernhardt et al., 2012). However, heterologous expression of *AtPXN* in various mutant yeast strains defective in CoA or NADH transport suggested that AtPXN does not transport either CoA or NADH but may instead function as an NAD/adenosine monophosphate (AMP) antiporter, importing cytosolic NAD^+^ via counter-exchange with peroxisomal AMP (van Roermund et al., 2016).

In human cells, cytosolic NAD^+^ is transported into mitochondria via the mitochondrial NAD^+^ transporter SLC25A51 and its homolog SLC25A52 (Luongo et al., 2020). Findings on mammalian peroxisomal NAD^+^ transporters are still controversial. In 2012, *in vitro* experiments indicated that the human peroxisome-localized transporter SLC25A17 transports CoA and NAD^+^, but not ATP (Agrimi et al., 2012a). Subsequently, the zebrafish (*Danio rerio*) Slc25a17 homolog was shown to transport CoA *in vivo*, but not NAD^+^ (Kim et al., 2020). Additionally, the contents of CoA, ATP, NAD^+^, and other cofactors in the peroxisomes of mice (*Mus musculus*) lacking Slc25a17 did not change significantly (Van Veldhoven et al., 2020), suggesting that Slc25a17 is not a mammalian peroxisomal NAD^+^ transporter and that a true peroxisomal NAD^+^ transporter remains to be identified.

## NADP^+^ biosynthesis from NAD^+^

**Figure 2:**
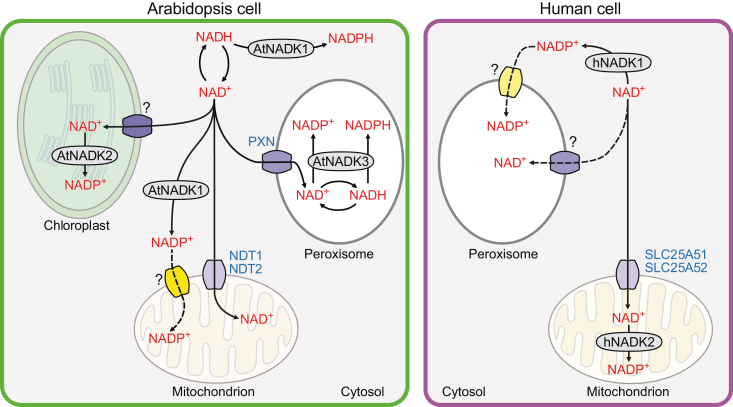
*De novo* NADP^+^ biosynthesis in *Arabidopsis* and human cells. Arabidopsis has three NADK isoforms: AtNADK1–3. Cytosolic AtNADK1 and peroxisomal AtNADK3 can use both NAD^+^ and NADH, although their preferred substrate is NADH. In humans, there are two NADK isoforms, cytoplasmic hNADK1 and mitochondrial hNADK2, both of which prefer NAD^+^ as a substrate. As Arabidopsis mitochondria lack NADK activity, cytosolic NADP^+^ may be transported into mitochondria through as-yet-unidentified transporters to supply mitochondrial cellular metabolism. Likewise, NADKs are absent from peroxisomes in human cells, so cytosolic NADP^+^ may enter peroxisomes via an unknown transporter.

NAD^+^ kinase (NADK) is the sole enzyme responsible for producing NADP(H) *de novo* via the phosphorylation of NAD(H) (Figure 2) (Kawai et al., 2001b; Pollak et al., 2007). NADKs are classified as NAD^+^ kinases and NADH kinases based on their preference for NAD^+^ or NADH, respectively (Li et al., 2018). NADKs are also classified as calmodulin (CaM)-independent or CaM-regulated isoforms based on their dependence on CaM. The CaM-regulated NADKs are activated under stress conditions to provide NADPH for an NADPH-dependent oxidative burst (Ruiz et al., 2002; Li et al., 2014).

The number of NADKs varies among species. Most prokaryotes only contain one NADK (Kawai et al., 2001a), whereas eukaryotes usually contain multiple NADKs that function in different organelles (Li et al., 2014). Humans contain two NADK isoforms: cytosolic hNADK1 and mitochondrial hNADK2 (also known as C5orf33). hNADK1 and hNADK2 both prefer NAD^+^, and only hNADK1 is regulated by CaM (Ohashi et al., 2012; Oka et al., 2023). Arabidopsis contains three NADK isoforms: cytosolic AtNADK1, chloroplast AtNADK2, and peroxisomal AtNADK3. Of these three Arabidopsis NADKs, only AtNADK2 is regulated by CaM and specifically uses NAD^+^. AtNADK1 and AtNADK3 utilize NAD(H), although NADH is their preferred substrate (Berrin et al., 2005; Li et al., 2018). Wheat (*Triticum aestivum*) contains four TaNADKs, among which TaNADK1 and TaNADK2 are located in the cytosol, TaNADK3 in chloroplasts, and TaNADK4 in peroxisomes (Wang et al., 2015). NADKc, a recently identified plant NADK, localizes to the outer membrane of mitochondria in Arabidopsis; NADKc is regulated by CaM and supplies NADP^+^ for the cytosolic oxidative pentose phosphate pathway (OPPP) (Dell'Aglio et al., 2019).

Intriguingly, no mitochondrial NADK has yet been identified in plants, and humans appear to lack a peroxisome-specific NADK (Figure 2). As various NADP^+^/NADPH-dependent enzymatic reactions take place in human peroxisomes, a transporter is likely to transport cytosolic NADP^+^ into peroxisomes, but it remains to be identified (Chornyi et al., 2020). Various studies in plants have demonstrated the existence of NADPH utilization pathways in plant mitochondria, but no plant mitochondrion-localized NADK or transporter for NADP^+^ into mitochondria has been identified or validated (Gakière et al., 2018; Moller et al., 2020).

## Why do organisms require two different energy currencies?

NADH is primarily involved in energy-producing catabolic reactions, whereas NADPH mainly participates in anabolic and antioxidative pathways (Oka et al., 2012; Ju et al., 2020). The two redox regulatory mechanisms of NADH and NADPH in cells are precise and non-conflicting, so how do the two mechanisms operate?

## The NADH redox system

In living cells, NADH is primarily generated via catabolic reactions, including glycolysis, pyruvate oxidation, the tricarboxylic acid (TCA) cycle, fatty acid oxidation, glycine oxidation, and glutamate oxidation (Supplemental Table 1). The most important role of NADH is to provide electrons for aerobic ATP production by the mitochondrial electron transport chain (mETC) to support fundamental cellular activities (Rasmusson et al., 2008; Schertl and Braun, 2014). In the following sections, we compare NADH metabolism in plants and mammals.

### NADH generation in plants

**Figure 3:**
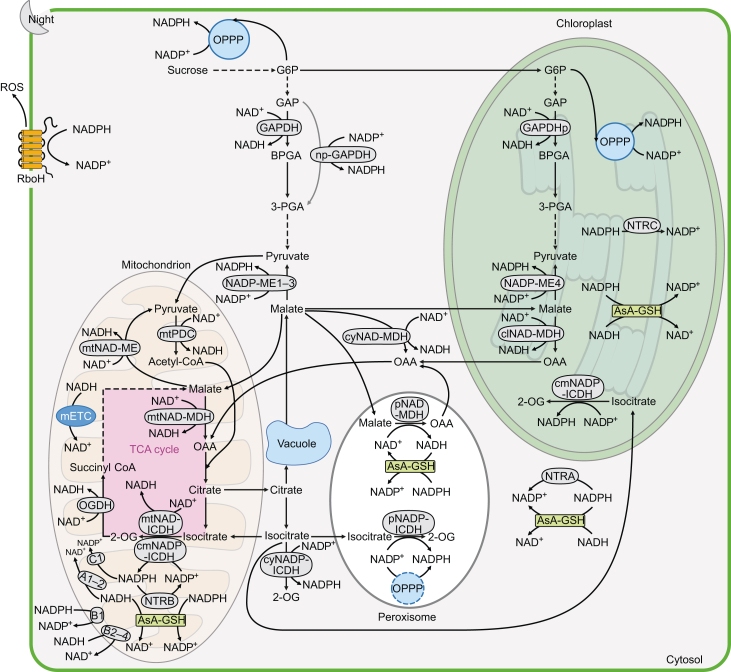
Major NAD(P)H production and consumption pathways in green tissues of *Arabidopsis* in the dark. In the dark, chloroplast/cytosolic glycolysis and the TCA cycle become more active. The sucrose and malate that accumulated during the day are released from the vacuole and can serve as sources of reducing equivalents. Sucrose can supply NADPH to chloroplasts and the cytosol via OPPP and NADH (via chloroplast GAPDH [GAPDHp] and cytosolic GAPDH, respectively) via glycolysis. Whether np-GAPDH can provide cytosolic NADPH at night requires validation. Malate stored in the vacuole during the day can be released to the cytosol during the night to supply cytosolic NADPH and NADH via NADP-ME1–3 and cyNAD-MDH, respectively. In addition, cytosolic malate can enter chloroplasts, mitochondria, and peroxisomes to supply NADH and NADPH via their respective MDHs and MEs. Specifically, clNAD-MDH and NADP-ME4 provide NADH and NADPH for chloroplasts, pNAD-MDH provides NADH for peroxisomes, and mtNAD-MDH and light-inactivated mtNAD-ME provide mitochondrial NADH. In mitochondria, OGDH in the TCA cycle also provides NADH. Pyruvate generated from glycolysis and cytosolic NADP-MEs enters the mitochondria to feed the TCA cycle. Citrate is exported from the mitochondria and stored in the vacuole during the night. Isocitrate can serve as a source of NADPH in the cytosol, mitochondria, peroxisomes, and chloroplasts via their respective NADP-ICDHs. NADH and NADPH in the matrix can be consumed by NDA1-2 (A1-2) and NDC1 (C1), respectively. During the night, the incomplete OPPP pathway in peroxisomes is driven by the import of plastid G6PD1 to provide NADPH. NADPH can participate in oxidative reactions throughout the day. Cytosolic NADH can be consumed by NDB2-4 (B2-4). Cytosolic NADPH can be consumed by NDB1 (B1), RboH, and NTRA; plastid-localized NADPH can be consumed through NTRC; and mitochondrial NADPH can be consumed through NTRB. The AsA–GSH cycle can consume both NADH and NADPH in the cytosol, chloroplasts, peroxisomes, and mitochondria regardless of light conditions. Actyl-CoA, acetyl coenzyme A; AsA–GSH, ascorbate–glutathione cycle; BPGA, 1,3-bisphosphoglycerate; cyNAD-MDH, cytosolic NAD-dependent malate dehydrogenase; clNAD-MDH, chloroplast NAD-dependent malate dehydrogenase; clNADP-MDH, chloroplast NADP-dependent malate dehydrogenase; cmNADP-ICDH, chloroplast-mitochondrial NADP-dependent isocitrate dehydrogenase; cyNADP-ICDH, cytosolic NADP-dependent isocitrate dehydrogenase; ETC, electron transport chain; FNR, ferredoxin-NADP(H) oxidoreductase; GAPDH, cytosolic NAD-specific glyceraldehyde 3-phosphate dehydrogenase; GAPDHp, plastidic NAD-specific glyceraldehyde 3-phosphate dehydrogenase; G6P, glucose 6-phosphate; GAP, glyceraldehyde 3-phosphate; mETC, mitochondrial electron transport chain; mPDC, mitochondrial pyruvate dehydrogenase complex; mNAD-ME, mitochondrial NAD-dependent malate enzyme; mtNAD-MDH, mitochondrial NAD-dependent malate dehydrogenase; mNAD-ICDH, mitochondrial NAD-dependent isocitrate dehydrogenase; np-GAPDH, non-phosphorylating glyceraldehyde 3-phosphate dehydrogenase; NADP-ME, NADP-dependent malate enzyme; NDA1-2 (A1-2), alternative NADH dehydrogenases A1-2; NDB1 (B1), alternative NADPH dehydrogenase B1; NDB2-4 (B2-4), alternative NADPH dehydrogenase B2-4; NDC1 (C1), alternative NADPH dehydrogenase C1; NTRA, NADPH-dependent thioredoxin reductase A; NTRB, NADPH-dependent thioredoxin reductase B; NTRC, NADPH-dependent thioredoxin reductase C; OGDH, 2-oxoglutarate dehydrogenase complex; OAA, oxaloacetate; OPPP, oxidative pentose phosphate pathway; pNAD-MDH, peroxisomal NAD-dependent malate dehydrogenase; pNADP-ICDH, peroxisomal NADP-dependent isocitrate dehydrogenase; RboH, respiratory burst oxidase homologs; Succinyl CoA, succinyl-coenzyme A; TCA cycle, tricarboxylic acid cycle; 3-PGA, 3-phosphoglycerate; 2-OG; 2-oxoglutarate.

Plants use different sources of NADH in the light and dark. In the dark, malate and complex carbon molecules that accumulated during the day fuel the production of NADH. Glycolysis is a biochemical process that predominantly occurs in the dark (Figure 3). During glycolysis, glyceraldehyde 3-phosphate dehydrogenases (GAPDHs) generate NADH by catalyzing the oxidation of glyceraldehyde-3-phosphate (GAP) to 1,3-bisphosphoglycerate. Cytosolic NAD-GAPDH (encoded by *GAPC*) and plastidic NAD-GAPDH (encoded by *GAPCp*) participate in glycolysis in the cytosol and plastid, respectively (Zeng et al., 2016). In photosynthetic cells, glycolysis in chloroplasts is mainly responsible for supplying stromal ATP at night (Voon et al., 2018). In non-photosynthetic cells, glycolysis occurs in both the cytosol and plastids throughout the day; both pathways are interconnected via highly selective transporters present on the inner plastid membrane (Petersen et al., 2003; Anoman et al., 2015, 2016).

Pyruvate oxidation connects glycolysis and the TCA cycle. During this process, pyruvate is converted to acetyl-CoA by the pyruvate dehydrogenase complex (PDC) to produce CO_2_ and NADH (Patel et al., 2014). Plant cells contain two PDC isoforms: a mitochondrion-localized PDC (mtPDC) and a plastid-localized PDC (plPDC). In green tissues, plPDC is active in the light and supplies acetyl-CoA for fatty acid biosynthesis, whereas mtPDC is active in the dark and feeds the TCA cycle (Budde and Randall, 1990; Tovar-Mendez et al., 2003).

**Figure 4:**
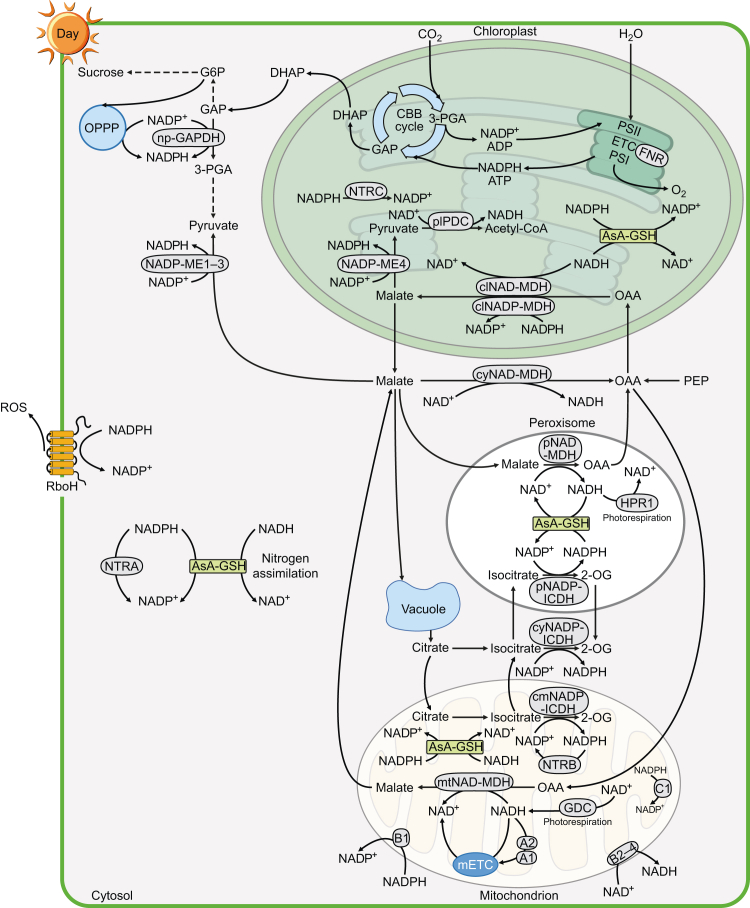
Major NAD(P)H production and consumption pathways in green tissues of *Arabidopsis* in the light. Under illumination, the photosynthetic light reactions are the main source of NADPH in the chloroplasts. During this process, electrons are transferred from water molecules to reduce NADP^+^ to NADPH via FNR, and NADPH is mainly consumed by the Calvin cycle. Malate accumulating in chloroplasts can be converted to pyruvate and provides NADPH via NADP-ME4. Pyruvate is then oxidized to acetyl-CoA and produces NADH in the light. NADH produced during this reaction can be consumed by clNAD-MDH during the conversion of OAA to malate. In the light, this step can also be catalyzed by clNADP-MDH. Excess reducing equivalents are exported from chloroplasts to the cytosol in the form of malate and triose-P; the latter is mainly used for sucrose biosynthesis. Malate can also be a source of cytosolic NADPH and NADH via the actions of NADP-ME1–3 and cyNAD-MDH, respectively. np-GAPDH and cytosolic OPPP can also supply cytosolic NADPH. The conversion of PEP into OAA in the cytosol is light dependent, offering an important source of cytosolic OAA during the day. Cytosolic OAA enters the mitochondria and is converted into malate by mtNAD-MDH. This reaction and the mETC consume large amounts of mitochondrial NADH provided by the glycine decarboxylation step of photorespiration via GDC. The excess NADH can also be consumed by NDA1-2 (A1-2), with NDA1 (A1) being significantly induced in the light. During the daytime, the tricarboxylic acid (TCA) cycle is not actually a cycle, as the activities of mtPDC, citrate synthase, NAD-ICDH, and OGDH are suppressed. Surplus mitochondrial NADH can be exported to the cytosol in the form of malate. Some cytosolic malate is imported into peroxisomes to generate NADH via pNAD-MDH and is then consumed in the hydroxypyruvate reduction step of photorespiration via HPR1. The remaining surplus cytosolic malate and synthesized sucrose are stored in the vacuole. Citrate accumulating in the vacuole at night can be released during the day via conversion to isocitrate in the cytosol and mitochondria by cyNADP-ICDH and cmNADP-ICDH, respectively, offering a supply of NADPH. Cytosolic isocitrate is transported to peroxisomes and can supply NADPH via pNADP-ICDH. In the light, cytosolic nitrogen assimilation is the major cytosolic NADH sink. Under stress conditions, the NADPH oxidase RboH can consume cytosolic NADPH. TrxRs also consume NADPH in the cytosol (NTRA), chloroplasts (NTRC), and mitochondria (NTRB). The AsA–GSH cycle can consume both NADH and NADPH in the cytosol, chloroplasts, peroxisomes, and mitochondria regardless of light conditions. In addition, NADPH in the matrix can be consumed by NDC1 (C1); cytosolic NADPH and NADH can be consumed by NDB1 (B1) and NDB2-4 (B2-4), respectively. DHAP, dihydroxyacetone phosphate; GDC, glycine decarboxylase; HPR1, hydroxypyruvate reductase 1; PEP, phosphoenolpyruvate; plPDC, plastidic pyruvate dehydrogenase complex; PSI, photosystem I; PSII, photosystem II.

Each acetyl-CoA molecule that is fed into the TCA cycle reduces three molecules of NAD^+^ to NADH. The first NADH molecule is generated by isocitrate dehydrogenase (ICDH), which converts isocitrate to 2-OG and reduces NAD(P)^+^ to NAD(P)H. In vascular plants, NAD-dependent ICDHs are strictly localized to mitochondria (Lemaitre and Hodges, 2006). The second molecule of NADH is produced by the conversion of 2-OG into succinyl-CoA via the mitochondrial multienzyme 2-oxoglutarate dehydrogenase complex (OGDH). The structure of this multienzyme is conserved across plants, mammals, and prokaryotes (Frank et al., 2007; Nemeria et al., 2014; Condori-Apfata et al., 2019). The third NADH molecule is produced by a reversible malate oxidation reaction via malate dehydrogenase (MDH). Arabidopsis has multiple NAD-MDH isoforms, including mitochondrial AtmtNAD-MDH1 and AtmtNAD-MDH2 (which function in the TCA cycle and photorespiration), three cytosolic MDHs (cyNAD-MDH1–3), and one plastidic MDH (plNAD-MDH), as well as two peroxisomal MDHs (pNAD-MDH1–2) involved in photorespiration (Selinski and Scheibe, 2019). In the light, the TCA cycle in photosynthetic cells does not operate as a cycle and does not provide a substantial amount of NADH in mitochondria as it does in the dark because the activities of mtPDC, mtNAD-ICDH, and OGDH are suppressed under illumination (Figure 4) (Sweetlove et al., 2010; Gardestrom and Igamberdiev, 2016; Fedorin et al., 2022; Igamberdiev and Bykova, 2023).

Glutamate oxidation can supply mitochondrial NADH through glutamate dehydrogenase (GDH), which reversibly deaminates glutamate, producing NAD(P)H and 2-OG. Plants contain distinct isozymes of GDH that are either NAD^+^ or NADP^+^ specific (Dubois et al., 2003; Qiu et al., 2019). Plant NAD-specific GDHs in mitochondria and NADP-specific GDHs in chloroplasts both function in nitrogen assimilation (Terce-Laforgue et al., 2004; Fontaine et al., 2012; Du et al., 2019).

### Photorespiration as a major source of NADH in C3 plants

In C3 plants, during photorespiration, decarboxylation and deamination of glycine, catalyzed by glycine decarboxylase (GDC) in mitochondria, generates NADH, ammonia (NH_3_), and CO_2_ (Douce et al., 2001). This is the major source of NADH for the mETC during photosynthesis (Hagemann and Bauwe, 2016; Lim et al., 2020). An abundant supply of NADH from photorespiration allosterically suppresses the activities of various TCA cycle enzymes (Gardestrom and Igamberdiev, 2016). C4 plants have evolved the capacity to spatially compartmentalize initial CO_2_ fixation and decarboxylation, which greatly increases the CO_2_ concentration around Rubisco, thereby minimizing photorespiration and diminishing the mitochondrial NADH pool (Sage, 2016; Schulze et al., 2016). In Kranz-type C4 plants, anaplerotic CO_2_ fixation occurs in mesophyll cells (MSCs), and malate decarboxylation and the Calvin–Benson–Bassham (CBB) cycle occur in bundle sheath cells (BSCs). In C4 plants, GDC protein is mainly expressed in BSC mitochondria, which primarily function in C1 metabolism, as photorespiration is significantly inhibited by the high CO_2_ concentration resulting from the C4 mechanism (Hylton et al., 1988; Schulze et al., 2016). By contrast, the C4 plant *Bienertia sinuspersici* achieves spatial compartmentalization in a single cell using dimorphic chloroplasts (i.e., peripheral and central chloroplasts) (Offermann et al., 2011, 2015).

C4 plants are divided into the NADP-malic enzyme (NADP-ME), NAD-malic enzyme (NAD-ME), and phosphoenolpyruvate carboxykinase (PEPCK) subtypes, but no pure PEPCK C4 species has been identified to date. In the NAD-ME subtype, Asp generated from OAA in MSC chloroplasts is transported to mitochondria in the BSCs and converted back to OAA via AspAT and then to malate by mtNAD-MDH. The malate is then decarboxylated by NAD-ME to release CO_2_ and NADH (Rao and Dixon, 2016). Light significantly induces *NAD-MEα* expression in NAD-ME C4 but not C3 *Cleome* species (Hudig et al., 2022). By contrast, in the C3 plant Arabidopsis, the activities and expression of two mitochondrial *NAD-ME* genes (*mtNAD-ME1* and *mtNAD-ME2*) decrease during the day (Tronconi et al., 2008). The high expression of *mtNAD-ME2* could represent an adaptation of NAD-ME C4 plants. For example, *mtNAD-ME2* is expressed at higher levels in *B. sinuspersici* than in Arabidopsis, and this gene is also expressed at much higher levels in NAD-ME C4 grasses than in C3 grasses (Rao and Dixon, 2016; Watson-Lazowski et al., 2018; Han et al., 2023). Unlike *mtNAD-ME*, *mtNAD-MDH* transcription did not markedly differ between C3 and NAD-ME C4 *Cleome* species, and mtNAD-MDH enzyme activity was high in both species, suggesting that the capacity of mtNAD-MDH is sufficient in both C3 and C4 *Cleome* species (Brautigam et al., 2011; Sommer et al., 2012).

### NADH generation from the glyoxylate cycle of plants

In addition to the main TCA cycle in the mitochondrial matrix, plants also employ a unique cycle that bypasses the TCA cycle known as the glyoxylate cycle, which occurs in glyoxysomes (Graham, 2001; Pracharoenwattana et al., 2005; Kunze and Hartig, 2013). Glyoxysomes are specialized microbodies found in plant cells, particularly in oil-rich germinating seeds such as in soybean (*Glycine max*), Arabidopsis, and sunflower (*Helianthus annuus*). Germinating oilseeds mainly rely on the degradation of their oil reserves as a source of carbon backbones and energy. The core glyoxylate cycle involves the conversion of acetyl-CoA to succinate, with each cycle producing one NADH molecule that is generated via the oxidation of malate by glyoxysomal NAD-MDH. In leaves, glyoxysomes disappear and peroxisomes appear when photosynthesis is initiated (Oikawa et al., 2019; De Bellis et al., 2020). During the phototrophic stage, fatty acids are oxidized via β-oxidation in peroxisomes to generate NADH for plant growth under limited carbon conditions (Goepfert and Poirier, 2007; Yu et al., 2019).

### NADH generation in mammalian cells

In mammals, glycolysis occurs only in the cytosol and generates NADH via a single GAPDH (Tristan et al., 2011; Ikeda et al., 2012). Subsequently, pyruvate, the end product of glycolysis, is transported into the mitochondria and oxidized by PDC to generate NADH and acetyl-CoA, the latter entering the TCA cycle. As in plants, each turn of the TCA cycle in mammals produces three NADH molecules, which are produced sequentially by ICDH, OGDH, and MDH. Humans contain three ICDHs: hICDH1–hICDH3. hICDH3 generates NADH in mitochondria, and the other two enzymes generate NADPH (Reitman and Yan, 2010). The human MDH isoforms hMDH1 and hMDH2 are both NAD^+^ specific, and, while mitochondrial hMDH2 is involved in the TCA cycle, hMDH1 localizes to the cytosol and peroxisomes (McCue and Finzel, 2022). Malate is also decarboxylated via NAD(P)-ME to provide NAD(P)H. Human cells contain three malic enzymes—hNADP-ME1, hNADP-ME2, and hNAD(P)-ME3—with hNADP-ME1 localized to the cytosol and the other two enzymes localized to mitochondria. hNAD(P)-ME3 participates in the TCA cycle and generates NADH, and it also produces NADPH for lipid biosynthesis and glutathione reduction (Hsieh et al., 2019).

In mammals, fatty acid β-oxidation occurs and generates NADH in both mitochondria and peroxisomes (Poirier et al., 2006). As mammals only obtain sugars from their diet, when cells contain insufficient glucose levels, fat reserves are converted into fatty acids via lipolysis. The resulting fatty acids undergo β-oxidation in the mitochondria to produce NADH, thereby supporting ATP production via the mETC (Elmadfa and Kornsteiner, 2009). The oxidation of amino acids also provides NADH in mammalian cells. In mammals, glycine oxidation via GDC functions in mitochondrial folate metabolism (Pai et al., 2015). Unlike plant GDHs, mammalian mitochondrial GDHs use both NAD^+^ and NADP^+^, allowing mitochondria to use glutamate as an NADH source for the mETC when required (Bunik et al., 2016; Plaitakis et al., 2017).

### Major sources of NADH for mitochondrial ATP production in mammals versus plants

In mammals, the TCA cycle is the main source of NADH for ATP production in mitochondria. In plants, however, the sources of mitochondrial NADH vary in different tissues, developmental stages, light conditions, and photosynthetic types. During oilseed germination, β-oxidation and the glyoxylate cycle convert stored lipids into succinate, which is transported to mitochondria for NADH production (Voon and Lim, 2019). In the photosynthetic tissues of C3 plants, the glycine decarboxylation step of photorespiration supplies a large amount of NADH to mitochondria, exceeding the NADH-consuming capacity of the mETC, and the surplus NADH is exported to the cytosol in the form of malate (Lim et al., 2020). Hence, in the photosynthetic tissues of C3 plants, photorespiration and the TCA cycle are the major sources of NADH for ATP production in mitochondria during the day and night, respectively. In the non-photosynthetic tissues of plants and in the photosynthetic tissues of C4 plants, the TCA cycle represents the major source of fuel for ATP production in mitochondria during the day.

### NADH consumption in plants

Under aerobic conditions, NADH produced in mammal and plant mitochondria is mainly consumed through the mETC (Efremov and Sazanov, 2012; Lapuente-Brun et al., 2013; Sazanov, 2014). Here, NADH is consumed by complex I to generate the proton motive force needed to produce ATP through ATP synthase. However, the system is more sophisticated in plants. Alternative oxidase (AOX) and alternative NAD(P)H dehydrogenases (NDs) in plant mitochondria only transfer electrons without proton translocation and thus do not directly contribute to oxidative phosphorylation. AOX directly transfers electrons from ubiquinone to O_2_ to generate water. NDs located on the inner or outer surface of the inner mitochondrial membrane transfer electrons from NAD(P)H in the mitochondrial intermembrane space or matrix to ubiquinone (UQ), which is then transferred to complexes III–IV or AOX (Rasmusson et al., 2008; McDonald et al., 2009; Antos-Krzeminska and Jarmuszkiewicz, 2019). Arabidopsis contains seven ND isoforms divided into three subfamilies: NDA, NDB, and NDC. NADH-dependent NDA1-2 (NDin) are located on the inner surface of the mitochondrial inner membrane. Light significantly induces the transcription of *AtNDA1* and it might function in the dissipation of mitochondrial NADH produced by photorespiration (Elhafez et al., 2006). NADPH-dependent NDB1 (NPDex) and NADH-dependent NDB2-NDB4 (NDex) are located on the outer surface of the inner mitochondrial membrane (Schertl and Braun, 2014; Moller et al., 2021). AtNDBs have a low pH optimum, and the activities of AtNDB1 and AtNDB2 were strongly and moderately stimulated by Ca^2+^, respectively (Geisler et al., 2007). Some stress conditions, such as hypoxia and ammonium and salt treatments, increase cytosolic Ca^2+^ level, which then activates Ca^2+^-dependent AtNDB1 and AtNDB2, thereby alleviating ROS generation by consuming cytosolic NAD(P)H (Igamberdiev and Hill, 2018; Rasmusson et al., 2020; Bachani et al., 2022). The activity of NADPH-dependent NDC1, located on the inner surface of the mitochondrial inner membrane, is also stimulated by Ca^2+^ and low pH (Rasmusson and Moller, 1991). Under certain stress conditions, concurrent upregulation of ND and AOX genes was observed, which helps plants to decrease UQ pool reduction and prevent excessive generation of ROS (Moller, 2001; Van Aken et al., 2009; Vanlerberghe, 2013; Sweetman et al., 2019; Rasmusson et al., 2020).

Fermentation is a common anaerobic pathway that consumes NADH without ATP production. When cellular O_2_ concentrations are below a given threshold, pyruvate in the plant cytosol is reduced to lactate by lactate dehydrogenase (LDH) or converted to acetaldehyde by PDC, followed by further reduction to ethanol by acetaldehyde dehydrogenase (Dolferus et al., 2008; Wei et al., 2009).

In plant nitrogen assimilation, nitrate (NO_3_^−^) reduction is catalyzed by nitrate reductase (NR), the activity of which is light activated in photosynthetic tissues (Riens and Heldt, 1992). NR is present in the plant cytosol and uses NAD(P)H as its electron donor. In most vascular plants, NR uses NADH as an electron donor, whereas a bi-specific form of NR that uses NADH/NADPH is present in monocotyledons and some dicotyledons (e.g., soybean); NR in mosses and fungi uses NADPH as the electron donor (Tischner and Kaiser, 2007). In vascular plants, nitrite (NO_2_^−^), a product of nitrate reduction, is transported to plastids, converted to ammonium (NH_4_^+^) by nitrite reductase (NIR), and participates in the GS–GOGAT cycle (Takahashi et al., 2001; Gupta et al., 2022). In the GS–GOGAT cycle, Gln is converted to Glu by GOGAT, which uses reduced ferredoxin (Fd_red_) as the electron donor in photosynthetic tissues and NADH as the electron donor in non-photosynthetic tissues (Kojima et al., 2014).

In C3 plants, toxic glyoxylate is generated during photorespiration in peroxisomes and is rapidly converted to glycine via glyoxylate aminotransferase. Glyoxylate transported to the cytosol and chloroplasts is detoxified by NAD(P)H-dependent glyoxylate reductase (GR). In Arabidopsis and rice, there are two GR isoforms, cytosolic GR and chloroplast GR, and both prefer NADPH rather than NADH as a cofactor (Simpson et al., 2008; Zhang et al., 2020b). Peroxisomal glycine is transported and converted to serine in mitochondria, which is exported and converted to hydroxypyruvate in peroxisomes and further reduced to glycerate using NADH via hydroxypyruvate reductase (HPR). Glycerate is further converted to 3-phosphoglyceric acid (3-PGA) in chloroplasts to complete the photorespiration cycle. Arabidopsis has three HPR isoforms: peroxisome-localized NADH-dependent AtHPR1, cytosolic NADPH-dependent AtHPR2, and chloroplast-localized NADPH-dependent AtHPR3 (Timm et al., 2011). We suggest that the preferences of enzymes with NAD(P)H dual specificity have evolved as an adaptation to the prevailing concentrations of NADH and NADPH in their respective subcellular compartments. In both the cytosol and chloroplasts, where NADPH levels are higher than those of NADH, NADPH is preferred by GRs and by AtHPR2 and AtHPR3 (Gakière et al., 2018).

### NADH consumption in mammals

In mammals, NADH is mainly consumed through the mETC under aerobic conditions via complex I. Under anerobic conditions, mammalian cells perform lactate fermentation to recycle NAD^+^ via cytosolic LDH. In addition to functioning in the cytosolic lactate fermentation pathway, LDH is also found in mammalian peroxisomes, where it utilizes peroxisomal NADH to reduce pyruvate (Schueren et al., 2014). In mammals, glyoxylate is also toxic and is reduced to glycolate by the dual-localized bifunctional enzyme glyoxylate reductase/HPR using NAD(P)H in mitochondria and the cytosol. In peroxisomes, glyoxylate is metabolized to glycine by alanine/glyoxylate aminotransferase (Booth et al., 2006; Belostotsky et al., 2012; Salido et al., 2012; Garrelfs et al., 2024).

## The NADPH redox system

NADPH is a key electron donor for ROS detoxification and a variety of anabolic pathways, including fatty acid and nucleic acid biosynthesis, the CBB cycle, carotenoid biosynthesis, and proline biosynthesis (Aghdam et al., 2020; Moller et al., 2020). A major difference between mammalian cells and plant cells is that plant cells contain chloroplasts, which have a profound influence on the plant NADPH regulatory system (Supplemental Table 2).

### NADPH in plant chloroplasts/plastids

In photosynthetic tissues under illumination, photosynthesis is the primary source of NADPH production in chloroplasts (Figure 4). Linear electron flow (LEF; powered by sunlight) is the key electron flow in the photosystems, in which electrons are transferred from water to reduce NADP^+^ to NADPH by ferredoxin-NADP(H) oxidoreductase (FNR) (Morigasaki et al., 1990; Hanke et al., 2005; Mulo, 2011). By contrast, in non-photosynthetic tissues and in photosynthetic tissues in the dark, OPPP is the major source of NADPH in plastids (Figure 3). Plants have two complete OPPPs: one in the cytosol and one in plastids. Both pathways convert one molecule of glucose-6-phosphate to ribulose-5-phosphate to generate two molecules of NADPH via glucose-6-phosphate dehydrogenase (G6PD) and 6-phosphogluconate dehydrogenase (6PGD). There are six G6PDs in Arabidopsis, namely plastid-localized G6PD1–4 and cytosolic G6PD5 and G6PD6 (Wakao and Benning, 2005). The Arabidopsis genome encodes three 6PGDs: PGD1 and PGD3 localize in the cytosol and plastids, and PGD2 localizes in the cytosol and peroxisomes (Holscher et al., 2016).

Plant chloroplasts/plastids also contain other NADPH-producing enzymes, including NADP-ICDH, NADP-MDH, and NADP-ME. Four NADP-ICDH isozymes were identified in Arabidopsis, including one isoenzyme localized to peroxisomes, one to the cytosol, one to mitochondria, and one to chloroplasts. Notably, the mitochondrion-localized and chloroplast-localized NADP-ICDH isozymes are encoded by the same gene (Hodges et al., 2003; Leterrier et al., 2016).

NADP-MDH is localized to the chloroplast (Ocheretina et al., 2000) (Figure 4). In C3 plants, the enzymatic activity of NADP-MDH is light activated and contributes to the export of surplus reducing equivalents from the chloroplast to the cytosol via malate export (Foyer et al., 2009; Lim et al., 2020; Yokochi et al., 2021). During the evolution of NADP-ME C4 plants, the enzyme activity and transcript abundance of NADP-MDH have been shaped to support C4 photosynthesis. In this type of photosynthesis, OAA is converted to malate by NADP-MDH in MSCs, which is then transported to BSCs and decarboxylated via NADP-ME to release CO_2_ via NADP-ME (Rao and Dixon, 2016). Although the *NADP-MDH* gene exists as a single copy in both C3 and NADP-ME C4 *Flaveria* species, the enzyme activity and transcript abundance of NADP-MDH are much higher in the latter. In addition, light significantly induced the transcription of *NADP-MDH* in C4 *Flaveria* species and maize (*Zea mays*) but had no effect on this gene in C3 *Flaveria* species (Metzler et al., 1989; McGonigle and Nelson, 1995; Lyu et al., 2025).

Similar to *NADP-MDH*, the transcription of *NADP-ME* in NADP-ME C4 *Flaveria* species is induced by light, and its enzyme activity is much higher in NADP-ME C4 *Flaveria* species than in C3 *Flaveria* species (Lyu et al., 2025). Arabidopsis contains only one chloroplast NADP-ME isoform (NADP-ME4). By contrast, in maize, the original *non-C4-NADP-ME* gene was duplicated and an additional *C4-NADP-ME* gene evolved (Wheeler et al., 2005; Alvarez et al., 2013; Böhm et al., 2025). The enzymatic activity of maize C4 NADP-ME is much higher than that of maize non-C4 NADP-ME and AtNADP-ME4 (Maier et al., 2011). In addition, maize *C4-NADP-ME* is expressed at much higher levels in leaves compared to *non-C4-NADP-ME*, and UV treatment significantly increased the expression of maize *C4-NADP-ME* but had little effect on non-C4 and cytosolic *NADP-ME*s (Alvarez et al., 2013).

In chloroplasts, NADPH generated via the light reactions of photosynthesis is mainly used in the CBB cycle via NADP-specific GAPDH (Marri et al., 2009; Zeng et al., 2016). This pool of NADPH is also used by thioredoxins, as well as for lipid and chlorophyll biosynthesis (Mulo, 2011). In non-photosynthetic plastids, root FNR reduces Fd using NADPH derived from the OPPP to drive various metabolic pathways (Hachiya et al., 2016; Guan et al., 2018). In vascular plants, nitrite reduction during nitrogen assimilation also occurs in plastids/chloroplasts, a process catalyzed by NIR using Fd_red_ as an electron donor in photosynthetic chloroplasts and NADPH as an electron donor in non-photosynthetic plastids (Joy and Hageman, 1966; Takahashi et al., 2001).

NADPH is also involved in ROS detoxification, which is important for defense responses and signal transduction. In chloroplasts, ROS is mainly produced via the Mehler reaction, which generates superoxide (O_2_^−^) from O_2_ in photosystem I; the resulting ROS is detoxified via the ascorbate–glutathione (AsA–GSH) cycle and the thioredoxin (Trx) system, both of which use NADPH as an electron donor. In the AsA–GSH cycle, glutathione reductase (GTR) uses NADPH as a source of reducing equivalents to catalyze the reduction of oxidized glutathione (GSSG) to reduced glutathione (GSH) (Noctor et al., 2012). GSH is then converted back to GSSG by dehydroascorbate reductases (DHARs). Electrons from hydrogen peroxide (H_2_O_2_) are then dissipated by the oxidation of ascorbate (AsA) to monodehydroascorbate (MDHA) via ascorbate peroxidase. AsA is recycled either from dehydroascorbate via DHAR or from MDHA via NAD(P)H-dependent monodehydroascorbate reductase (MDAR) (Noctor et al., 2000). Arabidopsis contains two GTR isoforms: AtGTR1 localizes in the cytosol, nucleus, and peroxisomes and AtGTR2 is targeted to plastids and mitochondria (Foyer and Noctor, 2011; Marty et al., 2019). Arabidopsis also contains five MDAR isoforms: cytosolic/peroxisomal AtMDAR1, cytosolic AtMDAR2 and AtMDAR3, peroxisomal AtMDAR4, and chloroplastic/mitochondrial AtMDAR5. AtMDARs have different NAD(P)H preferences, with AtMDAR1 and AtMDAR5 preferring NADH and AtMDAR2 preferring NADPH (Vanacker et al., 2018; Tanaka et al., 2021). In the Trx system, peroxiredoxins, a class of cysteine-dependent peroxidases, are reduced by NADPH oxidation via thioredoxin reductase (TrxR) and Trx (Hebbelmann et al., 2012). In Arabidopsis and rice, three NADPH-dependent TrxRs (NTRs) with disulfide-bond reductase activity have been reported: NTRA, the major cytosolic NTR; NTRB, the major mitochondrion-localized NTR, sharing a redundant function in the cytosol and mitochondria with NTRA; and chloroplast-localized NTRC (Cha et al., 2015).

### NADPH in the cytosol

NADPH levels in eukaryotic cells are usually lower in the cytosol than in organelles (Tao et al., 2017; Lim et al., 2020). As mentioned above, the OPPP is an essential source of NADPH in plant plastids and cytosol; in mammals, the OPPP is only found in the cytosol (TeSlaa et al., 2023). In addition to the OPPP, cytosolic NADPH is also produced via several NADPH-generating enzymes, including non-phosphorylating GAPDH (np-GAPDH), NADP-ICDH, and NADP-ME. np-GAPDH, an enzyme mainly found in autotrophic eukaryotes, catalyzes the oxidation of GAP to 3-PGA in the cytosol and generates one molecule of NADPH (Bustos and Iglesias, 2003; Wieloch, 2021). Cytosolic NADP-ICDH activity has been detected in all Arabidopsis tissues, with high activity in leaves (Mhamdi et al., 2010). Arabidopsis contain three cytosolic NADP-ME isoforms, AtNADP-ME1–3, and only *AtNADP-ME2* is continuously expressed in leaves and roots (Wheeler et al., 2005). In humans, hICDH1 and hNADP-ME1 generate NADPH in the cytosol, and the latter plays a role in cytosolic lipogenesis (Reitman and Yan, 2010; Hsieh et al., 2014).

In plants, cytosolic NADPH provides reducing power for members of the respiratory burst oxidase homolog (RboH) family. RboHs are a class of transmembrane proteins that mediate the transfer of electrons from intracellular NADPH to extracellular O_2_ to give O_2_^−^, which is subsequently catabolized to H_2_O_2_ by superoxide dismutase (Kaur and Pati, 2016; Foyer and Noctor, 2020). In mammals, the transmembrane enzymes NADPH oxidases catalyze electron transfer from cytosolic NADPH across the cell membrane to generate H_2_O_2_ (Torres and Dangl, 2005; Nazari et al., 2023). The H_2_O_2_ is detoxified by the cytosolic AsA–GSH cycle and the Trx systems using NADPH as a cofactor. In Arabidopsis, AtGTR1 and NTRA consume NADPH and function in the cytosolic AsA–GSH cycle and the Trx system, respectively. In humans, a single gene, *hGTR*, encodes both cytosolic and mitochondrion-localized isoenzymes that function in the AsA–GSH cycle in each compartment (Kelner and Montoya, 2000). The two major TrxR isoforms in humans, cytosolic TrxR1 and mitochondrial TrxR2, function in redox signaling in the corresponding organelles (Misevičienė et al., 2022). Notably, in plants, cytosolic NAD(P)H can also be consumed by NDBs located on the outer surface of the inner mitochondrial membrane, thereby participating in cytosolic redox metabolism (Rasmusson et al., 2008, 2020).

### NADPH in mitochondria

The mitochondrion is an important site for ROS production during cellular oxidative phosphorylation in both plant and mammalian cells (Lenaz, 2001; Moller, 2001; Navrot et al., 2006). In humans, hNADK2 generates NADP^+^ in mitochondria, which is then converted to NADPH by mitochondrion-localized NADPH-generating enzymes including hICDH2, hNADP-ME2, hNAD(P)-ME3, and proton-translocating transhydrogenase (Reitman and Yan, 2010; Ohashi et al., 2012; Hsieh et al., 2014, 2019; Francisco et al., 2022). Proton-translocating transhydrogenase, also known as nicotinamide nucleotide transhydrogenase (NNT), catalyzes the reversible transfer of hydride from NADH to NADP^+^ coupled to inward proton translocation. This enzyme is only found in the inner membranes of animal mitochondria and the plasma membranes of some prokaryotes (Jackson, 2012; Kastaniotis et al., 2017). In mammals, the electrochemical proton gradient (Δp) generated by respiration is mainly consumed by the NNT forward reaction. As a result, the pH of the mammalian mitochondrial matrix is 0.2 units lower than that of the plant mitochondrial matrix, which makes the [NADPH][NAD^+^]/[NADP^+^][NADH] ratio two orders of magnitude greater in mammalian than in plant mitochondria (Jackson, 2003; Shen et al., 2013; Gakière et al., 2018; Zou et al., 2018). In mammals, NADP^+^ produced by mitochondrial NADK and NADH generated by mitochondrial β-oxidation provide sufficient substrate for NNT, and the NADPH produced by NNT is consumed during fatty acid biosynthesis (Kastaniotis et al., 2017). As fatty acid biosynthesis in plants occurs in plastids and beta-oxidation occurs in peroxisomes, NNT is not needed to provide large amounts of NADPH in plant mitochondria and there is no evidence that it is present (Bykova et al., 1999; Moller et al., 2020).

NADPH in the plant mitochondrial matrix is mainly consumed by NDC and the NADPH-dependent ROS detoxification system, NTRB-Trx system, and AsA–GSH cycle via GTR2 (Moller and Rasmusson, 1998; Moller, 2001; Rasmusson et al., 2008, 2020; Cha et al., 2015; Marty et al., 2019). Although the activity of the mitochondrion-localized NADPH-generating enzyme NADP-ICDH has been detected in Arabidopsis and pea (*Pisum sativum*) (Rasmusson and Moller, 1990; Igamberdiev and Gardestrom, 2003; Leterrier et al., 2016), no mitochondrial NADK has been identified in plants. NADP^+^ was shown to be transported across the inner membrane of the plant mitochondrion (Bykova and Moller, 2001), but this result has not been verified, and there are no known plant mitochondrial membrane carriers for NADP^+^ (Moller et al., 2020). Therefore, the origin of plant mitochondrial NADP^+^ requires further investigation.

### NADPH in peroxisomes

Peroxisomes are highly dynamic organelles with pivotal roles in various metabolic pathways, such as fatty acid oxidation and glyoxylate metabolism. Many enzymes involved in these pathways generate distinct types of ROS. In mammalian peroxisomes, H_2_O_2_ is mainly metabolized into H_2_O by catalase or transported into the cytosol by peroxisomal membrane protein 2, thus maintaining redox balance in peroxisomes (Rokka et al., 2009; Fransen et al., 2012). Therefore, in mammals, peroxisomal NADPH mainly functions in anabolic pathways. In humans, NADP-dependent hICDH1, which localizes to both the cytosol and peroxisomes, provides NADPH for the biosynthesis of fat and cholesterol in peroxisomes (Reitman and Yan, 2010; Van Veldhoven, 2010). However, human cells lack NADK in their peroxisomes and thus require a transporter to transport cytosolic NADP^+^ into peroxisomes (Chornyi et al., 2020). The identity of this transporter remains unknown, and peroxisomal NADPH metabolism in mammalian cells also needs to be elucidated.

By contrast, NADK is present in plant peroxisomes, and the pathways for NADPH production in plant peroxisomes are more numerous. Plant peroxisomal NADP-ICDH (pNADP-ICDH) produces NADPH in peroxisomes (Leterrier et al., 2016). An incomplete OPPP present in plant peroxisomes also generates NADPH. In Arabidopsis, PGD2 is present in peroxisomes. However, Arabidopsis G6PD isoforms do not carry distinct C-terminal peroxisomal targeting signal 1 (PTS1) or N-terminal PTS2 motifs, and the interaction between G6PD4 and G6PD1 facilitates the import of G6PD1 into peroxisomes to complete the OPPP (Corpas et al., 1998; Meyer et al., 2011). Plant peroxisomal NADPH participates in ROS detoxification via the peroxisomal AsA–GSH cycle (Del Rio and Lopez-Huertas, 2016; Marty et al., 2019).

## Pyridine nucleotide pools and ratios in plants

NAD(H) and NADP(H) pools and the ratios of reduced/oxidized forms vary among different tissues and compartments and change dynamically under different conditions. *In vivo*, most NAD(P)H molecules are protein-bound, and the concentration of free NAD(P)H is relatively low (Agius et al., 2001; Kasimova et al., 2006; Smith et al., 2021). In plants, the levels of free and total NADH in cytosol under darkness were estimated to be 0.5 and 18 μM, respectively, and their levels in mitochondria were 70 and 190 μM, respectively, which were higher than the free NADH levels in human cell cytosol (0.12 μM) and mitochondria (30 μM) (Heineke et al., 1991; Igamberdiev and Gardestrom, 2003; Kasimova et al., 2006; Zhao et al., 2011). The combined levels of free NADP^+^ (38 μM) and NADPH (150 μM) in plant mitochondria and cytosol were also higher than that of human cell mitochondria (0.19 μM for NADP^+^ and 37 μM for NADPH) and cytosol (0.1 μM for NADP^+^ and 3.1 μM for NADPH) (Heineke et al., 1991; Tao et al., 2017; Zou et al., 2018).

Early studies employed *in vitro* methods to measure the subcellular concentrations of pyridine nucleotides in plant organelles rapidly fractionated from protoplasts. Under illumination, free NADPH and NADP^+^ levels are higher in spinach leaf chloroplasts than in the cytosol and mitochondria, and the NADPH/NADP^+^ ratio in the stroma under light (0.5) is a double of the ratio in the dark (0.23) (Heineke et al., 1991). The NADH level in mitochondria is higher than in chloroplasts and cytosol, and the NADH/NAD^+^ ratio in the cytosol is much lower than that in mitochondria and chloroplasts (Heineke et al., 1991; Szal et al., 2008). Illumination of pea leaves increased mitochondrial total NADH level from 75 to 455 μM and the NADH/NAD^+^ ratio from 0.05 to 0.29, and, in cytosol, total NADH level increased from 18 to 55 μM and the ratio from 0.03 to 0.1. These changes disappeared when photorespiration was inhibited by saturating CO_2_ level (Igamberdiev and Gardestrom, 2003). Similarly, the NADH/NAD^+^ ratio in the mitochondria of barley (*Hordeum vulgare*) protoplasts dropped from 0.22 to 0.07 when photorespiration was inhibited by high CO_2_ concentration under illumination (Wigge and Krömer, 1993). In plant and mammalian cells, the ratio of NADH/NAD^+^ is usually lower than that of NADPH/NADP^+^ (Wigge and Krömer, 1993; Zou et al., 2018). These two coenzyme couples have a marked difference in their reduction levels in plant matrix, as they are not in thermodynamic equilibrium but in a kinetic steady state (Moller et al., 2020).

The recent application of fluorescent biosensors in plants has enabled more sensitive and efficient monitoring of the dynamic changes in free pyridine nucleotide contents in different subcellular compartments *in planta* (Smith et al., 2021). An increase in the NADH/NAD^+^ ratio upon illumination was observed in Arabidopsis stroma using the *in planta* biosensor SoNar; this increase disappeared when photorespiration was inhibited. This analysis also revealed that the changes in the cytosolic NADH/NAD^+^ ratio were dependent on light intensity, with the ratio increasing at 296 mmol m^−2^ s^−1^ and decreasing at 40 mmol m^−2^ s^−1^ (Lim et al., 2020). NADH/NAD^+^ ratios and NADPH levels are differentially regulated in plants (Lim et al., 2020; Liu et al., 2022). Notably, 3 min of illumination increased the stromal NADPH level and NADH/NAD^+^ ratio. Both readings dropped after the light was turned off; the NADH/NAD^+^ ratio dropped to below basal levels, and the NADPH level only decreased during the first minute of darkness and subsequently stabilized above the basal level (Lim et al., 2020). These differences are due to the differential regulation of NAD(P)-MDH activities. While stromal NADP-MDH is activated by light and inactivated in the dark, the activation of NAD-MDHs in various organelles occurs independently of light. Mitochondrial NAD-MDH, due to its equilibrium properties and NADH/NAD^+^ buffering ability, rapidly recycles NAD^+^ from NADH, thereby alleviating the inhibition of mtPDC and GDC activities under high rates of respiration and photorespiration (Hagedorn et al., 2004; Bykova et al., 2014; Igamberdiev et al., 2014). These MDHs allow the rapid transport and storage of surplus reducing equivalents generated from photosynthesis and photorespiration in the form of malate, which accumulates in the vacuole during the day, and supplies NADH to various compartments in the dark (Figures 3 and 4).

## Concluding remarks and perspectives

The NADH and NADPH systems are robust and complex and have evolved to become increasingly sophisticated. Due to the endosymbiotic origin of chloroplasts, the functions of organelles in plant cells are more specialized than those in mammalian cells. In mammalian cells, mitochondria are enriched in both NADH and NADPH metabolic pathways. By contrast, plant mitochondria serve as powerhouses of the cell by consuming reductants harvested by chloroplasts and providing the cytosol with ATP and are therefore enriched in NADH metabolic pathways (Gardestrom and Igamberdiev, 2016). Plant chloroplasts capture solar energy and are involved in various anabolic pathways, resulting in the marked enrichment of NADPH-related metabolism.

More research on plant NAD(H) and NADP(H) metabolism is needed. The identity of chloroplast NAD^+^ transporter is still unclear. Homologs of AtNDT1, AtNDT2, and AtPXN can be searched among chloroplast transmembrane proteins and their NAD^+^ transport capacity can be examined. The origin of NADP^+^ in plant mitochondria remains to be elucidated, and the NADP^+^ transporter in the plant mitochondrial carrier family remains to be revealed.

Reduction and oxidation of pyridine nucleotides consume or release protons and these processes could affect subcellular pH. Their effect on pH is determined by the net release or uptake of protons in conjunction with concomitant reactions (Igamberdiev and Kleczkowski, 2019; Smith et al., 2021). For example, NADPH produced by Fd-FNR is consumed in the CBB cycle. Hence, the contribution to pH stat via proton release and consumption in the pyridine nucleotide redox reactions warrants further studies.

Under sufficient light, photosynthetic plant cells produce a surplus of reducing equivalents that will generate ROS, deplete NADP^+^, and impede LEF. This depletion of NADP^+^ particularly affects C3 photosynthesis, as tremendous NADH generated by photorespiration indirectly restricts stromal NADP^+^ regeneration from the malate valve (Lim et al., 2020). Therefore, inhibiting photorespiration by introducing photorespiratory bypasses (Xin et al., 2015; Shen et al., 2019) and enhancing NAD(P)H metabolism could improve energy efficiency of C3 plants. For example, overexpression of *AtPAP2* improved photosynthetic efficiency and productivity by enhancing chloroplast and mitochondrial activities, optimizing NAD(P)H metabolism, and increasing the NADP^+^/NADPH ratio (Sun et al., 2012; Liang et al., 2015; Voon et al., 2018; Cai et al., 2022). Other strategies such as optimizing NAD(P)H metabolic fluxes, enhancing enzyme stability, and reducing ATP costs, are potential pathways for improving energy-use efficiency in plants.

## Funding

This research was funded by the Hong Kong Research Grants Council General Research Fund (17102322), a 10.13039/501100000268Biotechnology and Biological Sciences Research Council grant (BB/W018748/1), the Area of Excellence Scheme (AoE/M-403/16), and the 10.13039/501100010428Innovation and Technology Fund (Funding Support to State Key Laboratory of Agrobiotechnology) of the Hong Kong Special Administrative Region, China. This project was also supported by the 10.13039/501100001809National Natural Science Foundation of China (32070394)

## Acknowledgments

No conflict of interest is declared.

## Author contributions

D.L. prepared the first draft, figures, and tables. B.L.L. and M.G. reviewed and edited the manuscript, figures, and tables. B.L.L. supervised the project. All authors have read and approved the final version of the manuscript.

## Supplemental information

Document S1. Supplemental Figure 1, Supplemental Tables 1, and 2

Document S2. Article plus supplemental information
