# Supplementary material for: NAD(H) and NADP(H) in plants and mammals
Source: Mol Plant. 2025 Jun 2;18(6):938–59. doi: 10.1016/j.molp.2025.05.004 (PMC12178909; doi:10.1016/j.molp.2025.05.004)
Supplement: Document S1. Supplemental Figure 1, Supplemental Tables 1, and 2 [file mmc1.pdf]

**Molecular Plant, Volume *18***

## Supplemental information

# NAD(H) and NADP(H) in plants and mammals

**Danying Lu, Murray Grant, and Boon Leong Lim**

**Supplemental Information**

**Supplemental Figure S1.** $NAD^+$ biosynthetic pathways in prokaryotes.

**Supplemental Table S1.** NADH generation and consumption pathways in Arabidopsis and Humans.

**Supplemental Table S2.** NADPH generation and consumption pathways in Arabidopsis and Humans.

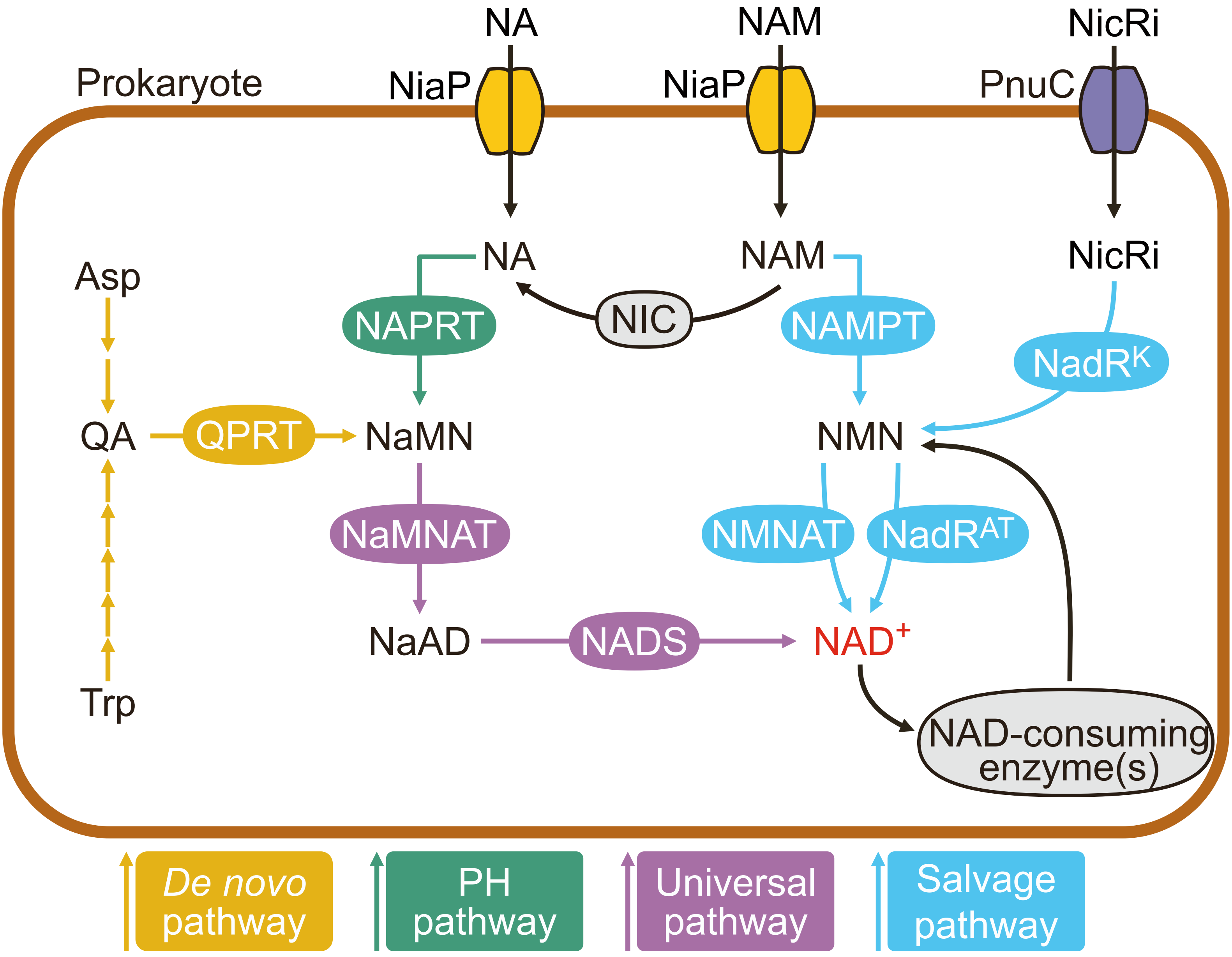

Prokaryote
NA
NAM
NicRi
NiaP
NiaP
PnuC
NA
NAM
NicRi
Asp
NAPRT
NIC
NAMPT
NadRK
QA
QPRT
NaMN
NMN
NaMNAT
NMNAT
NadRAT
NaAD
NADS
NAD+
Trp
NAD-consuming enzyme(s)
De novo pathway
PH pathway
Universal pathway
Salvage pathway

**Supplemental Figure S1. NAD$^+$ biosynthetic pathways in prokaryotes.**

In prokaryotes, NAD$^+$ can be synthesized via the *de novo* pathway, the Preiss–Handler (PH) pathway, or the salvage pathway. In the *de novo* pathway, QA is produced from Asp or Trp, which is then converted to NaMN by QPRT. NaMN is converted to NaAD and then to NAD$^+$ by NaMNAT and NADS, respectively. In the PH pathway, NA is converted to NaMN via NAPRT, which then enters the universal pathway to generate NAD$^+$. The salvage pathway uses NAM as a substrate to generate NAD$^+$ via NAMPT and NMNAT, or it uses NicRi as a substrate via two separate domains of NadR: NadR$^{AT}$ and NadR$^{K}$. In prokaryotes, NAM can be converted to NA via NIC. Exogenous NA and NAM are taken up by the transporter NiaP, and NicRi is taken up by the NicRi transporter PnuC.

Asp, aspartate; NA, nicotinic acid; NAM, nicotinamide; NaMN, nicotinate mononucleotide; NaAD, nicotinate adenine dinucleotide; NADS, NAD synthetase; Na/NMNAT, nicotinate/nicotinamide mononucleotide adenylyltransferase; NAPRT, nicotinate phosphoribosyltransferase; NAMPT, nicotinamide phosphoribosyl transferase; nadR$^{AT}$, nicotinamide mononucleotide adenylyltransferase; nadR$^{K}$, nicotinamide riboside kinase; niaP, niacin transporter; NIC, nicotinamidase; NMN, nicotinamide mononucleotide; NicRi, nicotinamide riboside; PnuC, NicRi uptake permease; QA, quinolinic acid; QPRT, quinolate phosphoribosyltransferase; Trp, tryptophan.

**Supplemental Table S1. NADH generation and consumption pathways in Arabidopsis and Humans.**

| Pathway | | Enzyme | Arabidopsis | | | Humans | | |
|---|---|---|---|---|---|---|---|---|
| | | | **isoform** | **subcellular location** | **reference** | **isoform** | **subcellular location** | **reference** |
| **Major NADH generation pathways** | Glycolysis | glyceraldehyde 3-phosphate dehydrogenase (GAPDH) | NAD-GAPDH | cytosol | Zeng et al., 2016 | GAPDH | cytosol | Ikeda et al., 2012; Tristan et al., 2011 |
| | | | NAD-GAPDHp | plastid | | - | - | - |
| | Pyruvate oxidation | pyruvate dehydrogenase complex (PDC) | mtPDC | mitochondria | Tovar-Mendez et al., 2003 | PDC | mitochondria | Patel et al., 2014 |
| | | | plPDC | plastid | | - | - | - |
| | TCA cycle | isocitrate dehydrogenase (ICDH) | NAD-ICDH | mitochondria | Lemaitre and Hodges, 2006 | NAD-ICDH | mitochondria | Reitman and Yan, 2010 |
| | | 2-Oxoglutarate dehydrogenase complex (OGDH) | OGDH | mitochondria | Condori-Apfata et al., 2019 | OGDH | mitochondria | Nemeria et al., 2014 |
| | | malate dehydrogenase | mtNAD-MDH | mitochondria | Lim et al., 2020; | mtNAD-MDH | mitochondria | McCue and Finzel, |

| | | | | | | | | |
|---|---|---|---|---|---|---|---|---|
| | | (MDH) | cyNAD-MDH | cytosol | Selinski and Scheibe, 2019 | cpNAD-MDH | cytosol, peroxisome | 2022 |
| | | | plNAD-MDH | plastid | | - | - | - |
| | | | pNAD-MDH | peroxisome | | - | - | - |
| | | NAD-malate enzyme (NAD-ME) | NAD-ME | mitochondria | Tronconi et al., 2008 | NAD-ME | mitochondria | Hsieh et al., 2019 |
| | Fatty acid β-oxidation | L-3-hydroxyacyl-CoA dehydrogenase (HADH) | HADH | peroxisome | Goepfert and Poirier, 2007 | HADH | mitochondria, peroxisomes | Van Veldhoven, 2010 |
| | Glyoxylate cycle | MDH | NAD-MDH | glyoxysome | De Bellis et al., 2020 | - | - | - |
| | Glycine oxidation | Glycine decarboxylase (GDC) | GDC | mitochondria | Douce et al., 2001 | GDC | mitochondria | Pai et al., 2015 |
| | Glutamate oxidation | glutamate dehydrogenase (GDH) | NAD-GDH | mitochondria | Qiu et al., 2019 | GDH | mitochondria | Bunik et al., 2016; Plaitakis et al., 2017 |

| | | | | | | | | |
|---|---|---|---|---|---|---|---|---|
| **Major NADH consumption pathways** | mETC | Complex I | Complex I | mitochondria | Efremov and Sazanov, 2012; Lapuente-Brun et al., 2013 | Complex I | mitochondria | Efremov and Sazanov, 2012; Lapuente-Brun et al., 2013 |
| | | Alternative NADH dehydrogenase | NDA1-2 | inner side of the mitochondrial inner membrane | Rasmusson et al., 2008; Rasmusson et al., 2020 | - | - | - |
| | | | NDB2-4 | outer side of the mitochondrial inner membrane | | - | - | - |
| | Fermentation | lactate dehydrogenase (LDH) | LDH | cytosol | Dolferus et al., 2008 | LDH | cytosol, peroxisome | Schueren et al., 2014 |
| | | acetaldehyde dehydrogenase (ADH) | ALDH | cytosol | Wei et al., 2009 | - | - | - |
| | Nitrogen assimilation | nitrate reductase (NR) | NR | cytosol | Tischner and Kaiser, 2007 | - | - | - |

| | | | | | | | | |
|---|---|---|---|---|---|---|---|---|
| | GS/GOGAT cycle | GOGAT | root-GOGAT | plastid | Kojima et al., 2014 | - | - | - |
| | Glyoxylate and hydroxypyruvate reduction | glyoxylate reductase (GR) | NAD(P)H-cyGR | cytosol | Simpson et al., 2008 | glyoxylate reductase/ hydroxypyruvate reductase (GRHPR) | cytosol, mitochondria | Booth et al., 2006 |
| | | | NAD(P)H-plGR | plastid | | | | |
| | | NAD(P)H-hydroxypyruvate reductase (HPR) | NADPH-cyHPR | cytosol | Timm et al., 2011 | | | |
| | | | NADH-pHPR | peroxisome | | | | |
| | | | NADPH-chlHPR | chloroplast | | | | |

**Supplemental Table S2. NADPH generation and consumption pathways in Arabidopsis and Humans.**

| Pathway | | Arabidopsis | | | | Mammal | | | |
|---|---|---|---|---|---|---|---|---|---|
| | | Enzyme | isoform | subcellular location | reference | Enzyme | isoform | subcellular location | reference |
| **Major NADPH generation pathways** | Photosyhthesis | Ferredoxin-NADP(H) oxidoreductase (FNR) | FNR | chloroplast | Morigasaki et al., 1990 | - | - | - | - |
| | The oxidative pentose phosphate pathway (OPPP) | Glucose-6-phosphate dehydrogenase (G6PD) | cyG6PD | cytosol | Wakao and Benning, 2005 | Glucose-6-phosphate dehydrogenase | G6PD | cytosol | TeSlaa et al., 2023 |
| | | | plG6PD | plastid | | | - | - | - |
| | | 6-Phosphogluconate dehydrogenase (6PDG) | PGD | cytosol, plastid, peroxisome | Holscher et al., 2016 | 6-Phosphogluconate dehydrogenase | PGD | cytosol | TeSlaa et al., 2023 |
| | | Malate dehydrogenase (MDH) | NADP-MDH | chloroplast | Lim et al., 2020; Yokochi et al., 2021 | - | - | - | - |

| | | | | | | | | | |
|---|---|---|---|---|---|---|---|---|---|
| | | NADP-malate enzyme (NADP-ME) | cyNADP-ME | cytosol | Wheeler et al., 2005 | NADP-malate enzyme (NADP-ME) | cyNADP-ME | cytosol | Ohashi et al., 2012 |
| | | | plNADP-ME | plastid | | | mNADP-ME | mitochondria | |
| | | Isocitrate dehydrogenase (ICDH) | cyNADP-ICDH | cytosol | Lemaitre and Hodges, 2006; Leterrier et al., 2016; Rasmusson and moller, 1990 | Isocitrate dehydrogenase (ICDH) | cyNADP-ICDH | cytosol, peroxisome | Reitman and Yan, 2010 |
| | | | cmNADP-ICDH | chloroplast, mitochondria | | | mNADP-ICDH | mitochondria | |
| | | | pNADP-ICDH | peroxisome | | - | - | - | - |
| | | Non-phosphorylating GAPDH | np-GAPDH | cytosol | Wieloch, 2021 | - | - | - | - |
| **Major NADPH consumption pathways** | Calvin cycle | NADP-specific GAPDH | NADP-GAPDH | chloroplast | Zeng et al., 2016 | - | - | - | - |
| | Alternative pathway of mETC | Alternative NADPH dehydrogenase | NDB1 | outer side of the mitochondrial inner membrane | Rasmusson et al., 2008; Rasmusson et al., 2020 | - | - | - | - |
| | | | NDC1 | inner side of the mitochondrial | | - | - | - | - |

| | | | | | | | | | |
|---|---|---|---|---|---|---|---|---|---|
| | | | | inner membrane | | | | | |
| | Root ferredoxin reduction | Root ferredoxin | Root-FNR | plastid | Guan et al., 2018; Hachiya et al., 2016 | - | - | - | - |
| | Nitrite reduction | nitrite reductase (NIR) | NIR | plastid | Takahashi et al., 2001 | - | - | - | - |
| | Reactive oxygen species (ROS) production | respiratory burst oxidase homologs (RboHs) | RboH | cytosol | Kaur and Pati, 2016 | NADPH oxidases (NOXs) | NOX | cytosol | Nazari et al., 2023 |
| | Ascorbate-Glutathione (AsA-GSH) cycle | glutathione reductase (GTR) | GTR1 | cytosol, nucleus and peroxisomes | Foyer and Noctor, 2011 | glutathione reductase (GTR) | GTR | cytosol, mitochondria | Kelner and Montoya, 2000 |
| | | | GTR2 | plastids, mitochondria | Marty et al., 2019 | - | - | - | - |
| | | NAD(P)H-dependent monodehydroascorbate reductase | MDAR1 | cytosol, peroxisome | Tanaka et al., 2021 | - | - | - | - |
| | | | MDAR2 | cytosol | | | | | |
| | | | MDAR3 | cytosol | | | | | |

| | | | | | | | | | |
|---|---|---|---|---|---|---|---|---|---|
| | | (MDAR) | MDAR4 | peroxisomal membrane-attached | | | | | |
| | | | MDAR5 | chloroplast, mitochondrial | | | | | |
| | The thioredoxin antioxidant system | thioredoxin reductase (TrxR) | NTRA | major NTR isoform in cytosol, also found in mitochondria and nucleus | Cha et al., 2015 | thioredoxin reductase (TrxR) | TrxR1 | cytosol | Misevičienė et al., 2022 |
| | | | NTRB | major NTR isoform in mitochondria, also found in cytosol | | | TrxR2 | mitochondria | |
| | | | NTRC | chloroplast | | | | | |
