# Supplementary material for: NAD(H) and NADP(H) in plants and mammals
Source: Mol Plant. 2025 Jun 2;18(6):938–59. doi: 10.1016/j.molp.2025.05.004 (PMC12178909; doi:10.1016/j.molp.2025.05.004)
Supplement: Document S2. Article plus supplemental information [file mmc2.pdf]

CellPress
Partner Journal

Molecular Plant
Review article

NAD(H) and NADP(H) in plants and mammals

Danying Lu[1], Murray Grant[2] and Boon Leong Lim[1,3,*]

[1]School of Biological Sciences, University of Hong Kong, Pokfulam, Hong Kong, China

[2]School of Life Sciences, University of Warwick, Gibbet Hill Campus, Coventry, UK

[3]State Key Laboratory of Agrobiotechnology, The Chinese University of Hong Kong, Shatin, Hong Kong, China

***Correspondence: Boon Leong Lim (bllim@hku.hk)**

https://doi.org/10.1016/j.molp.2025.05.004

ABSTRACT

Nicotinamide adenine dinucleotide (NAD) and nicotinamide adenine dinucleotide phosphate (NADP) are essential metabolic coenzymes in prokaryotic and eukaryotic cells, with their reduced forms, NAD(P)H, serving as electron donors for myriad reactions. NADH is mainly involved in catabolic reactions, whereas NADPH is mainly involved in anabolic and antioxidative reactions. The presence of endosymbiont-derived organelles in eukaryotes has made the functional division of NADH and NADPH systems more complex. Chloroplasts in photoautotrophic eukaryotes provide additional sources of reductants, complicating the maintenance of the redox balance of NAD(P)$^+$/NAD(P)H compared with heterotrophic eukaryotes. In this review, we discuss the two redox systems in plants and systematically compare them with those in mammals, including the similarities and differences in the biosynthesis and subcellular transport of NAD$^+$, the biosynthesis of NADP$^+$, and metabolic reactions for the reduction and oxidation of NAD(P)H. We also review the regulation of pyridine nucleotide pools and their ratios in different plant subcellular compartments and the effects of light on these ratios. We discuss the advantages of having both NADH and NADPH systems, highlight current gaps in our understanding of NAD(P)H metabolism, and propose research approaches that could fill in those gaps. The knowledge about NADH and NADPH systems could be used to guide bioengineering strategies to optimize redox-regulated processes and improve energy-use efficiency in crop plants.

Key words: chloroplasts, mitochondria, pyridine nucleotides, redox

Lu D., Grant M., and Lim B.L. (2025). NAD(H) and NADP(H) in plants and mammals. Mol. Plant. **18**, 938–959.

INTRODUCTION

The oxidized and reduced forms of nicotinamide adenine dinucleotide (NAD$^+$/NADH) and nicotinamide adenine dinucleotide phosphate (NADP$^+$/NADPH) play essential roles in energy metabolism in all eukaryotes and prokaryotes. NAD$^+$ and NADP$^+$ "pick up" electrons and protons to generate NADH and NADPH, respectively. These reduced molecules serve as electron donors that drive hundreds of redox reactions in living organisms. Adenosine triphosphate (ATP), the energy currency of many biological activities, is generated via the breakdown of carbon molecules through glycolysis and oxidative phosphorylation. During oxidative phosphorylation, electrons are transferred from NADH to oxygen (Tourmente et al., 2015; Wilson, 2017; Zhang et al., 2020a). Therefore, NADH is a key molecule that supplies reducing power to many catabolic pathways. NADPH also serves as a key electron donor and is mainly involved in anabolic pathways and antioxidative reactions (Chandel, 2021).

In this review, we describe how the two different redox coupling systems (NAD$^+$/NADH and NADP$^+$/NADPH) are deployed in plants and other organisms and discuss the importance of having both redox systems. Moreover, we compare heterotrophic eukaryotes, which acquired mitochondria as their major powerhouses from one endosymbiotic event, with photoautotrophic eukaryotes, which acquired chloroplasts and mitochondria from two distinct endosymbiotic events (Bjorn and Govindjee, 2009). Comparing mammals and plants provides insights into the diversity of redox coupling across eukaryotes.

NAD$^+$ BIOSYNTHESIS

All living organisms require NAD(P)H to support their life activities. However, not all organisms synthesize NAD$^+$ or NADP$^+$ [NAD(P)$^+$ hereafter]. Indeed, several prokaryotes, such as *Haemophilus influenzae* and *Chlamydia trachomatis*, have lost the biosynthetic enzymes necessary for NAD(P)$^+$ biosynthesis and thus rely on the uptake of NAD(P)$^+$ from their hosts (Reidl et al., 2000; Fisher et al., 2014). Other prokaryotes generate NAD$^+$ via three pathways: the *de novo* pathway, the salvage pathway, and the Preiss–Handler (PH) pathway (Supplemental Figure 1). Any given prokaryotic species may contain one or more of these pathways (Kurnasov et al., 2003). Similar to prokaryotes, mammals utilize the *de novo* pathway, the salvage pathway,

 © 2025 The Author(s). Published by Elsevier Inc. on behalf of CAS Center for Excellence in Molecular Plant Sciences, Chinese Academy of Sciences, and Chinese Society for Plant Biology.
This is an open access article under the CC BY license (http://creativecommons.org/licenses/by/4.0/).

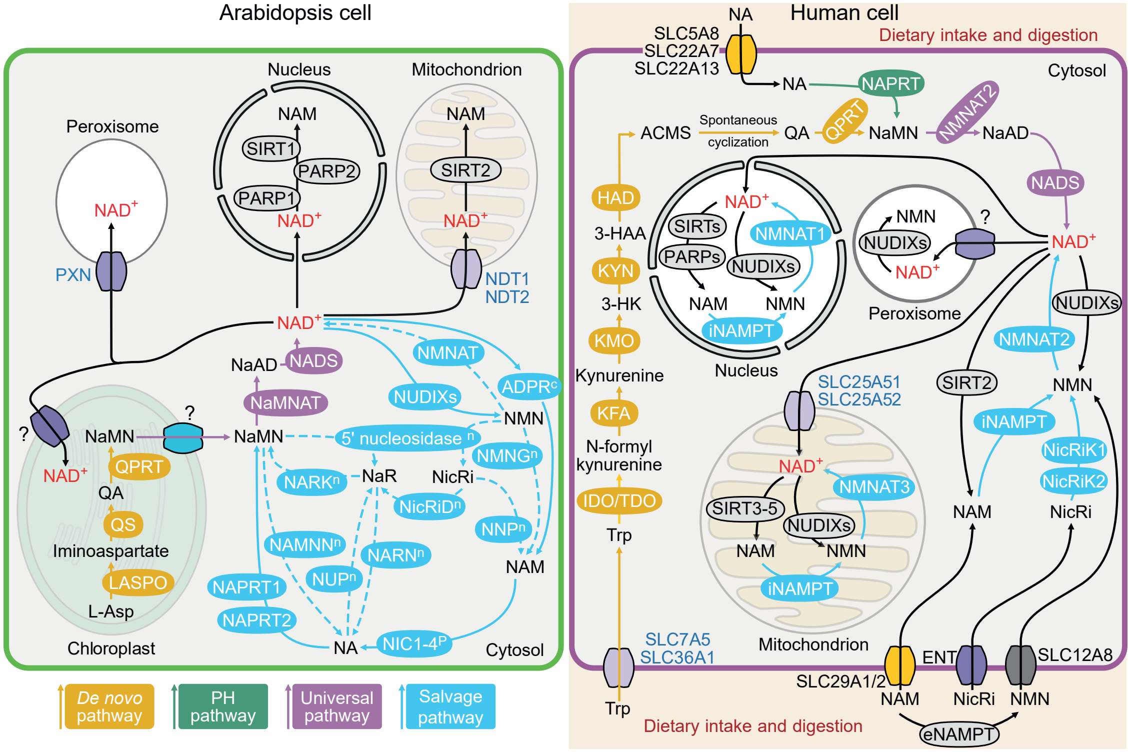

Figure 1. NAD$^+$ biosynthetic pathways in *Arabidopsis* and human cells.

Plants use the *de novo* pathway and the salvage pathway to synthesize NAD$^+$. In the *de novo* pathway, plants use Asp as the precursor to generate QA in plastids through a two-step enzymatic reaction catalyzed by LASPO and QS; QA is converted to NaMN by QPRT. NaMN is transported into the cytosol and used as a substrate to generate NaAD by NaMNAT. Finally, NaAD is converted to NAD$^+$ through NADS-mediated catalysis. The salvage pathway of plants primarily occurs in the cytosol and can be divided into two major recycling routes: via NicRi or via NAM. Cytosolic NAM can be generated from NAD$^+$ in two steps via NUDIXs and NMNG or directly from NAD$^+$ via ADPRc. NMN produced by NUDIX-catalyzed NAD$^+$ degradation may be directly converted back to NAD$^+$ via NMNAT. In addition, NicRi can generate NAM via NNP^n. NAM is then converted to NA via NIC1-4^P, and the resulting NA is further converted to NaMN by NAPRT1 and NAPRT2. Subsequently, NaMN enters the universal pathway to generate NAD$^+$. NaMN can be converted back to NA via NAMNN^n. NicRi is generated through NMN degradation via 5′ nucleosidases. NicRi is then converted to NaR via NicRiD^n. NaR is converted to NaMN through NaRK and enters the universal pathway to produce NAD$^+$. NaMN can be converted back to NaR via 5′ nucleosidases. NaR and NA can be interconverted via NARN^n and NUP^n, respectively. NAD$^+$ can be transported into mitochondria via the transporters NDT1 and NDT2 and degraded into NAM via SIRT2. NAD$^+$ can also enter the nucleus through the nuclear pore complex, where it will be degraded into NAM through the nucleus-localized NAD$^+$-consuming enzymes SIRT1, PARP1, and PARP2.

Human cells synthesize NAD$^+$ through three pathways: the *de novo* pathway, the salvage pathway, and the Preiss–Handler (PH) pathway. The human *de novo* pathway occurs strictly in the cytosol. Extracellular Trp is transported into the cytosol through SLC7A5 and SLC36A1 and is subsequently converted to *N*-formylkynurenine via IDO or TDO, whose product is converted to kynurenine by KFA. Kynurenine is converted to 3-HK by KMO, followed by the conversion of 3-HK to 3-HAA via HYN. 3-HAA is further converted to ACMS via HAD, after which ACMS is converted to QA through spontaneous cyclization. The resulting QA is converted to NaMN by QPRT, and NaMN is then converted to NaAD by NMNAT2. Finally, NaAD is converted to NAD$^+$ by NADS. In the PH pathway, extracellular NA enters the cytosol through SLC5A8, SLC22A7, and SLC22A13 before being converted to NaMN via NAPRT. NaMN then enters the universal pathway to generate NAD$^+$. The human salvage pathway takes place in the cytosol, nucleus, and mitochondria. In the cytosolic salvage pathway, extracellular NAM and NicRi are transported into the cytosol by SLC29A1/2 and ENT, respectively. Cytosolic NAM and NicRi are subsequently converted to NMN via iNAMPT and via NicRiK1 or NicRiK2, respectively. Extracellular NAM can also be converted to NMN by eNAMPT before entering the cytosol via SLC12A8. Cytosolic NMN is further converted to NAD$^+$ by NMNAT2. Cytosolic NAD$^+$ can be degraded to NAM by NAD-consuming enzymes (such as SIRT2) or to NMN via NUDIXs, the products of which can enter the recycling pathway. In addition, cytosolic NAD$^+$, NAM, and NMN can enter the nucleus through the nuclear pore complex. In the nucleus, NAD$^+$ can be degraded to NAM by SIRTs and PARPs, and the resulting NAM can be converted to NMN by iNAMPT. NMN can also be obtained from NAD degradation via nucleus-localized NUDIXs. NMN is ultimately converted to NAD$^+$ by NMNAT1. Cytosolic NAD$^+$ can also enter mitochondria through SLC25A51 and SLC25A52 to be converted to NAM via SIRT3–5. NAM is then converted to NMN by mitochondrial iNAMPT, and NMN is converted to NAD$^+$ by NMNAT3. Mitochondrial NAD can also be degraded into NMN by NUDIX. In mammalian cells, NUDIX is also present in peroxisomes; it is thought that cytosolic NAD$^+$ can enter peroxisomes through unknown transporters.

ACMS, 2-amino-3-carboxy-muconate-semialdehyde; ADPRc, ADP-ribose cyclase; Asp, aspartate; ENT, equilibrative nucleoside transporters; eNAMPT, extracellular nicotinamide phosphoribosyl transferase; HAD, 3-hydroxyanthranilic acid 3,4-dioxygenase; 3-HK, 3-hydroxy-kynurenine; 3-HAA, 3-hydroxyanthranilic acid; IDO, indoleamine 2,3-dioxygenase; iNAMPT, intracellular nicotinamide phosphoribosyl transferase; KFA, kynurenine formamidase; KMO, kynurenine-3-monooxygenase; KYN, kynureninase; LASPO, L-aspartate oxidase; NAM, nicotinamide; NADS, NAD synthetase; NaAD, nicotinate adenine dinucleotide; Na/NMNAT, nicotinate mononucleotide/nicotinamide mononucleotide adenylyltransferase; NaRK, nicotinate ribose kinase; NARN, nicotinate ribose nucleoside; NaR, nicotinate ribose; NAPRT, nicotinate phosphoribosyltransferase; NAMNN, nicotinate mononucleotide nucleosidase; NaMN, nicotinate mononucleotide; NA, nicotinic acid; NDT, NAD$^+$ transporters; NIC, nicotinamidase; NicRiD, nicotinamide riboside

(legend continued on next page)

and the PH pathway to synthesize NAD^+. By contrast, vascular plants only possess the *de novo* and salvage/recycling pathways, as, unlike mammals, plants effectively synthesize nicotinic acid (NA) (Hunt et al., 2004; Xiao et al., 2018) (Figure 1).

The *de novo* pathway

In the *de novo* pathway (Figure 1), NAD^+ is generated from quinolinic acid (QA), which is converted to nicotinate mononucleotide (NaMN) by quinolate phosphoribosyltransferase. NaMN is then adenylated to form nicotinate adenine dinucleotide (NaAD) by nicotinate mononucleotide/nicotinamide mononucleotide adenylyltransferase (NaMNAT/NMNAT), a bifunctional enzyme that also converts NAD^+ to nicotinamide mononucleotide (NMN) in the salvage pathway. Finally, NaAD is amidated to form NAD^+ via NAD synthetase. This biosynthetic pathway from QA to NAD^+ is highly conserved among prokaryotes, plants, and mammals (Rodionov et al., 2008; Gakière et al., 2018; Covarrubias et al., 2021). L-aspartate (Asp) and L-tryptophan (Trp) both serve as precursors for QA biosynthesis. The biosynthesis of QA from Asp is accomplished via a two-step reaction catalyzed by L-aspartate oxidase (LASPO) and quinolinate synthase (QS) (Katoh et al., 2006; Spaans et al., 2015). In the Trp-to-QA pathway, Trp is converted to 2-amino-3-carboxy-muconate-semialdehyde through a five-step enzymatic reaction, which then spontaneously cyclizes to QA (Figure 1) (Magni et al., 2008; Badawy, 2017).

Prokaryotes use Asp or Trp as a precursor for QA biosynthesis, but plants only utilize Asp, whereas mammals only utilize Trp (Kurnasov et al., 2003; Gakière et al., 2018; Xiao et al., 2018). Most prokaryotes, including *Escherichia coli*, *Bacillus subtilis*, and cyanobacteria, use Asp as a precursor for *de novo* NAD^+ biosynthesis, while some prokaryotes, such as *Xanthomonas pruni*, use Trp (Kurnasov et al., 2003; Gerdes et al., 2006; Lima et al., 2009). Cyanobacteria, a division of prokaryotes that perform photosynthesis and are thought to be the evolutionary ancestors of plant chloroplasts, solely utilize Asp for *de novo* NAD^+ biosynthesis, a pathway that might have been retained throughout evolution from cyanobacteria to vascular plants (Gerdes et al., 2006; Ishikawa and Kawai, 2019).

Differences in precursor choices in the *de novo* pathway in plants and mammals

Plants synthesize all 20 standard amino acids, whereas mammals synthesize only 11, including Asp. Therefore, mammals must obtain the nine remaining essential amino acids, including Trp, from their diet (Trovato et al., 2021) (Figure 1). Thus, it is worth reflecting on why plants might have evolved the ability to use Asp, whereas mammals evolved the ability to use Trp for their respective *de novo* NAD^+ biosynthesis pathways.

Efficiency and economy are important reasons for plants to use Asp rather than Trp for *de novo* NAD^+ biosynthesis. In plants, Trp biosynthesis begins with the shikimic acid pathway in plastids, which comprises a series of enzymatic reactions that require large amounts of energy. Furthermore, Trp is one of the least abundant amino acids in plants, with low abundance in solution (1–15 μM) within plant cells (Last, 1995). Compared to the complex and energetically costly Trp biosynthetic process, Asp biosynthesis is much more economical (Akashi and Gojobori, 2002). Aspartate transaminase (AspAT) directly converts glutamate (Glu) and oxaloacetate (OAA) to 2-oxoglutarate (2-OG) and Asp. In plants, Glu is *de novo* synthesized via nitrate assimilation, with the final step occurring in chloroplasts or plastids via the glutamine synthetase (GS)–glutamate synthase (GOGAT) cycle (Dragicevic et al., 2016). As a result, Glu is abundant in chloroplasts, making these organelles a good location for Asp production. It is therefore easy to understand why QA biosynthesis occurs in chloroplasts and why Asp was selected as the precursor for *de novo* NAD^+ biosynthesis during evolution: Asp biosynthesis is more energetically efficient than Trp biosynthesis.

In mammalian cells, *de novo* Asp biosynthesis primarily occurs through the transamination of OAA via AspAT (Ndrepepa, 2021). Asp is also obtained directly from the diet, as the amount of Asp derived from food is markedly higher than the amount of Trp (Gardner et al., 2019). In humans, Trp from the diet is transported into the cytosol via the solute carrier family members SLC7A5 and SLC36A1 (Larsen et al., 2009; Jiang et al., 2024). Nevertheless, it is surprising that mammals have lost the enzymes LASPO and QS, preventing them from using Asp as a precursor for *de novo* NAD^+ biosynthesis (Bossi et al., 2002; Gerdes et al., 2002). The computer-calculated values of the change in Gibbs free energy (ΔG) for the reactions catalyzed by LASPO and QS are −53.2 and −24.0 kcal/mol, respectively, indicating that these two reactions are highly favorable and irreversible (https://biocyc.org/). Accordingly, we suggest that, if these two enzymes were retained in mammals during evolution, most of the Asp obtained from the diet would be consumed for *de novo* NAD^+ biosynthesis, which would deplete the Asp pool available for protein synthesis. This may explain why mammals adopted the Trp pathway rather than the Asp pathway for *de novo* NAD^+ biosynthesis during their evolution. Notably, most mammalian cells do not express the genes encoding the enzymes necessary for the Trp-to-QA conversion pathway and rely on circulating nicotinamide (NAM) to NAD^+ via the salvage pathway (Liu et al., 2018; Covarrubias et al., 2021). This preference for the salvage pathway is attributed to its energetic efficiency compared with the high energy cost of *de novo* NAD^+ biosynthesis via Trp.

The salvage/recycling pathway

Following NAD^+ biosynthesis via the *de novo* pathway, NAD^+ is degraded to NAM by various NAD^+-consuming enzymes,

deaminase; NicRi, nicotinamide ribose; NUDIX, NUDIX hydrolases; NuP, nucleoside phosphorylase; NMN, nicotinamide mononucleotide; NMNG, nicotinamide mononucleotide glycohydrolase; NNP, nicotinate nucleoside pyrophosphatase; NicRiK, nicotinamide riboside kinase; PARP, poly(ADP-ribose) polymerases; PXN, peroxisomal NAD carrier; QA, quinolinic acid; QPRT, quinolate phosphoribosyltransferase; QS, quinolinate synthase; SLC, soluble carrier; SIRT, sirtuin; Trp, tryptophan; TDO, tryptophan 2,3-dioxygenase.

Superscript “P” indicates that the subcellular localizations of these enzymes were predicted by MULocDeep (https://mu-loc.org/).

Superscript “n” indicates that the enzymes and their encoding genes have not yet been identified in *Arabidopsis*, although their activities have been identified in other plants.

including NAD^+ glycohydrolases, polyADP-ribose polymerases (PARPs), and sirtuins (SIRTs). NAM itself is subsequently degraded to other by-products (Figure 1). In addition, NAD^+ is directly converted to NMN by NUDIX hydrolases (NUDIXs). The products of these reactions are recycled through the salvage pathway to regenerate NAD^+ (Hunt et al., 2004; Braidy et al., 2019; Kulikova and Nikiforov, 2020). In general, salvage pathways recycle NAD^+ enzymatically via NAM, nicotinamide ribose (NicRi), or NMN. Another NAD^+ recycling pathway, the PH pathway, uses NA as a substrate (Figure 1) (Preiss and Handler, 1958; Noctor et al., 2011). Precursors of the salvage and PH pathways in prokaryotes and mammals are obtained from the environment and diet, respectively, and through the recycling of NAD^+ degradation products. Most prokaryotes recycle NAD^+ using the NA, NicRi, or NAM pathway or two of these pathways (Supplemental Figure 1) (Gerdes et al., 2006; Rodionov et al., 2008), whereas mammals contain both a complete PH pathway and a salvage pathway (Figure 1). Due to the compartmentalization of mammalian cells, the NAD^+ recycling pathway is much more complex in mammals than in prokaryotes.

In humans, extracellular NA enters the cytosol via SLC5A8, SLC22A7, and SLC22A13 (Ganapathy et al., 2005; Bahn et al., 2008; Mathialagan et al., 2020). Cytosolic NA is subsequently converted to NaMN via nicotinate phosphoribosyltransferase (NAPRT), which then enters the universal pathway to regenerate NAD^+ (Figure 1) (Fricker et al., 2018; Mehmel et al., 2020; Chini et al., 2021). The human salvage pathway is active in the cytosol, mitochondria, and nucleus (Figure 1). In these compartments, NAD^+ is degraded into NAM and NMN by NAD^+-consuming enzymes and NUDIXs, respectively. NAM is then converted to NMN by intracellular nicotinamide phosphoribosyltransferase (NAMPT) in these compartments, and NMN is ultimately regenerated to form NAD^+ via the corresponding NMNAT (Berger et al., 2005; Michishita et al., 2005; Boehler et al., 2011; Bai and Canto, 2012; Kulikova and Nikiforov, 2020; Covarrubias et al., 2021). Mammals also contain extracellular NAMPT. This enzyme converts extracellular NAM to NMN, which enters the cytosol via the importer SLC12A8 (Travelli et al., 2018; Grozio et al., 2019). The extracellular NAM and NicRi are imported into cells through SLC29A1-2 and equilibrative nucleoside transporters, respectively (Kropotov et al., 2021; Chen et al., 2025).

Notably, the NAD^+ recycling pathway in plants differs greatly from those in animals and prokaryotes, as the precursors are all derived from NAD^+ degradation and the NAD^+ recycling pathway itself (Figure 1). In plants, the salvage pathway, which primarily functions in the cytosol, is divided into two recycling routes based on the source of precursors (NicRi or NAM). In the NAM recycling branch, NAD^+ is first degraded to NMN by cytosolic NUDIXs and is then converted to NAM via NMN glycohydrolase (NMNG) (Wagner et al., 1986; Yoshimura and Shigeoka, 2015). NAM is also generated from NAD^+ via ADP-ribose cyclase present at the plasma membrane (Sanchez et al., 2004). Subsequently, NAM generates NA via cytosolic nicotinamidase (NIC), and NA generates NaMN via NAPRTs before entering the universal pathway to regenerate NAD^+ (Gakière et al., 2018; Ahmad et al., 2021; Jiang et al., 2021). NaMN is also converted back to NA via NaMN nucleosidase (NAMNN) (Wagner et al., 1986). In the NicRi recycling branch, NMN is converted to NicRi by 5′ nucleosidase, and NaMN is then generated through a two-step reaction via NicRi deaminase (NicRiD) and nicotinate ribose kinase (NaRK), ultimately regenerating NAD^+. Notably, in this branch of the recycling pathway, NaMN is converted back to nicotinate ribose (NaR) by 5′ nucleosidase.

The intermediate products of these two recycling pathways are interconvertible, facilitating the flexible adjustment of the NAD^+ pool (Figure 1). NicRi is converted to NAM via nicotinate nucleoside pyrophosphatase (NNP). NaR is transformed to NA via NaR nucleoside (NARN), and the reverse reaction is catalyzed by nucleoside phosphorylase (NuP) (Matsui and Ashihara, 2008; Katahira and Ashihara, 2009; Ashihara and Deng, 2012). The plant salvage pathway is less well characterized. The presence, function, and encoding genes of NaRK, 5′ nucleosidase, NAMNN, NARN, NuP, NNP, NMNG, and NicRiD in Arabidopsis (*Arabidopsis thaliana*) are still unclear (Gakière et al., 2018). The enzyme activities of NaRK, 5′ nucleosidase, NARN, and NNP were detected in both potato (*Solanum tuberosum*) tubers and tea plant (*Camellia sinensis*) (Katahira and Ashihara, 2009; Ashihara and Deng, 2012). The enzyme activities of NAMNN and NMNG were detected in tobacco (*Nicotiana tabacum*) (Wagner et al., 1986). Furthermore, the enzyme activity of NuP was detected in mung bean (*Vigna radiata*) (Matsui and Ashihara, 2008), and the enzyme activity of NicRiD was detected in potato (Katahira and Ashihara, 2009).

As in mammals, NAD^+ synthesized in the plant cytosol is transported to different organelles and degraded (Figure 1). In Arabidopsis, mitochondrial NAD^+ is degraded to NAM via SIRT2, and NAD^+ in the nucleus is degraded to NAM through SIRT1, and PARP1-2 (Konig et al., 2014; Song et al., 2015; Liu et al., 2017). Arabidopsis NAD-metabolizing NUDIXs are only found in the cytosol, and the subcellular localizations and characteristics of several Arabidopsis NUDIXs still need to be verified (Yoshimura and Shigeoka, 2015). NMNAT activity was detected in the mitochondria of *Helianthus tuberosus* tubers, suggesting it may regenerate NAD^+ from NMN in mitochondria (Martino and Pallotta, 2011). The Arabidopsis genome contains only one gene encoding Na/NMNAT, which prefers NaMN, but it is unclear whether it encodes a mitochondrial isoform (Hashida et al., 2007). Therefore, evidence to support the existence of NAD^+ recycling pathways in the mitochondria, nucleus, or peroxisomes of Arabidopsis is not yet available (Figure 1).

NAD^+ TRANSPORT

In eukaryotic cells, the final step of *de novo* NAD^+ biosynthesis takes place only in the cytoplasm (Figure 1). Therefore, NAD^+ must be transported into organelles for specific metabolic pathways following its biosynthesis. The nuclear membrane is freely permeable to NAD^+ through nuclear pores (Fjeld et al., 2003), and cytosolic NAD^+ is also transported to multiple organelles via NAD^+ transporters. In Arabidopsis, three NAD^+ transporters—AtNDT1, AtNDT2, and PEROXISOMAL NAD^+ CARRIER (AtPXN)—have been identified, with AtNDT1 and AtNDT2 both localized to mitochondria (Douce and Neuburger, 1989; Chaves et al., 2019; Luo et al., 2019). Although early studies indicated that AtNDT1 localizes to the inner chloroplast membrane, more recent subcellular localization assays indicated that AtNDT1 is instead specifically present in the inner

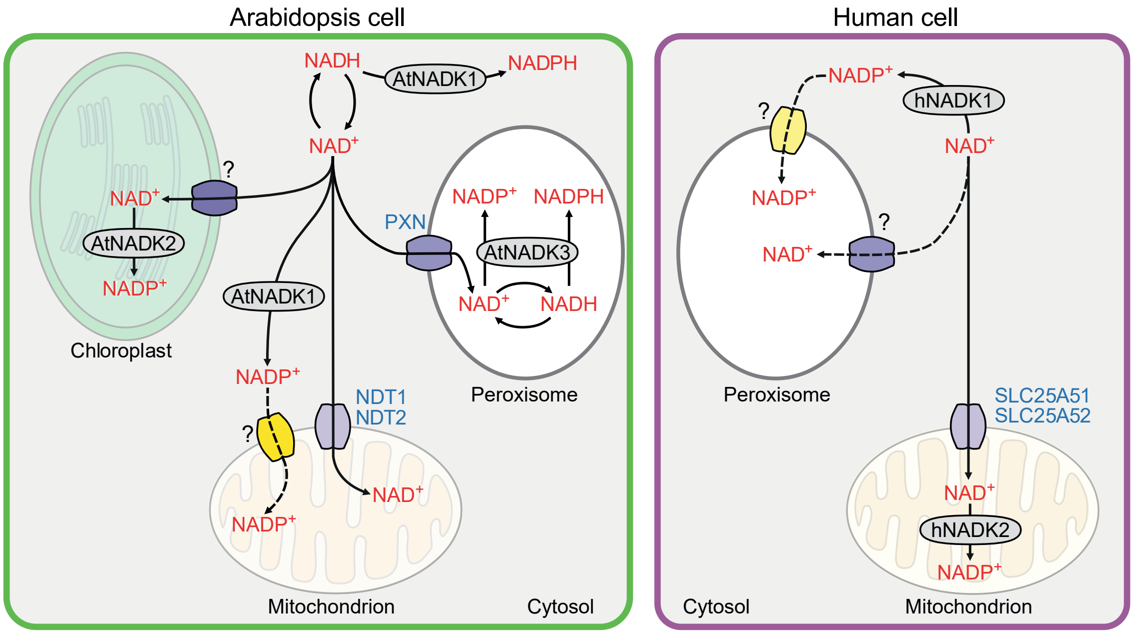

Figure 2. *De novo* NADP$^+$ biosynthesis in *Arabidopsis* and human cells.
Arabidopsis has three NADK isoforms: AtNADK1–3. Cytosolic AtNADK1 and peroxisomal AtNADK3 can use both NAD$^+$ and NADH, although their preferred substrate is NADH. In humans, there are two NADK isoforms, cytoplasmic hNADK1 and mitochondrial hNADK2, both of which prefer NAD$^+$ as a substrate. As Arabidopsis mitochondria lack NADK activity, cytosolic NADP$^+$ may be transported into mitochondria through as-yet-unidentified transporters to supply mitochondrial cellular metabolism. Likewise, NADKs are absent from peroxisomes in human cells, so cytosolic NADP$^+$ may enter peroxisomes via an unknown transporter.

mitochondrial membrane (Palmieri et al., 2009; Chaves et al., 2019). Therefore, exactly how plant plastids acquire NAD$^+$ from the cytosol remains to be elucidated. Early *in vitro* studies suggested that the peroxisomal NAD$^+$ transporter AtPXN could transport NAD(H) and coenzyme A (CoA) (Agrimi et al., 2012b; Bernhardt et al., 2012). However, heterologous expression of *AtPXN* in various mutant yeast strains defective in CoA or NADH transport suggested that AtPXN does not transport either CoA or NADH but may instead function as an NAD/adenosine monophosphate (AMP) antiporter, importing cytosolic NAD$^+$ via counter-exchange with peroxisomal AMP (van Roermund et al., 2016).

In human cells, cytosolic NAD$^+$ is transported into mitochondria via the mitochondrial NAD$^+$ transporter SLC25A51 and its homolog SLC25A52 (Luongo et al., 2020). Findings on mammalian peroxisomal NAD$^+$ transporters are still controversial. In 2012, *in vitro* experiments indicated that the human peroxisome-localized transporter SLC25A17 transports CoA and NAD$^+$, but not ATP (Agrimi et al., 2012a). Subsequently, the zebrafish (*Danio rerio*) Slc25a17 homolog was shown to transport CoA *in vivo*, but not NAD$^+$ (Kim et al., 2020). Additionally, the contents of CoA, ATP, NAD$^+$, and other cofactors in the peroxisomes of mice (*Mus musculus*) lacking Slc25a17 did not change significantly (Van Veldhoven et al., 2020), suggesting that Slc25a17 is not a mammalian peroxisomal NAD$^+$ transporter and that a true peroxisomal NAD$^+$ transporter remains to be identified.

NADP$^+$ BIOSYNTHESIS FROM NAD$^+$

NAD$^+$ kinase (NADK) is the sole enzyme responsible for producing NADP(H) *de novo* via the phosphorylation of NAD(H) (Figure 2) (Kawai et al., 2001b; Pollak et al., 2007). NADKs are classified as NAD$^+$ kinases and NADH kinases based on their preference for NAD$^+$ or NADH, respectively (Li et al., 2018). NADKs are also classified as calmodulin (CaM)-independent or CaM-regulated isoforms based on their dependence on CaM. The CaM-regulated NADKs are activated under stress conditions to provide NADPH for an NADPH-dependent oxidative burst (Ruiz et al., 2002; Li et al., 2014).

The number of NADKs varies among species. Most prokaryotes only contain one NADK (Kawai et al., 2001a), whereas eukaryotes usually contain multiple NADKs that function in different organelles (Li et al., 2014). Humans contain two NADK isoforms: cytosolic hNADK1 and mitochondrial hNADK2 (also known as C5orf33). hNADK1 and hNADK2 both prefer NAD$^+$, and only hNADK1 is regulated by CaM (Ohashi et al., 2012; Oka et al., 2023). Arabidopsis contains three NADK isoforms: cytosolic AtNADK1, chloroplast AtNADK2, and peroxisomal AtNADK3. Of these three Arabidopsis NADKs, only AtNADK2 is regulated by CaM and specifically uses NAD$^+$. AtNADK1 and AtNADK3 utilize NAD(H), although NADH is their preferred substrate (Berrin et al., 2005; Li et al., 2018). Wheat (*Triticum aestivum*) contains four TaNADKs, among which TaNADK1 and TaNADK2 are located in the cytosol, TaNADK3 in chloroplasts, and TaNADK4 in peroxisomes (Wang et al., 2015). NADKc, a recently identified plant NADK, localizes to the outer membrane of mitochondria in Arabidopsis; NADKc is regulated by CaM and supplies NADP$^+$ for the cytosolic oxidative pentose phosphate pathway (OPPP) (Dell'Aglio et al., 2019).

Intriguingly, no mitochondrial NADK has yet been identified in plants, and humans appear to lack a peroxisome-specific

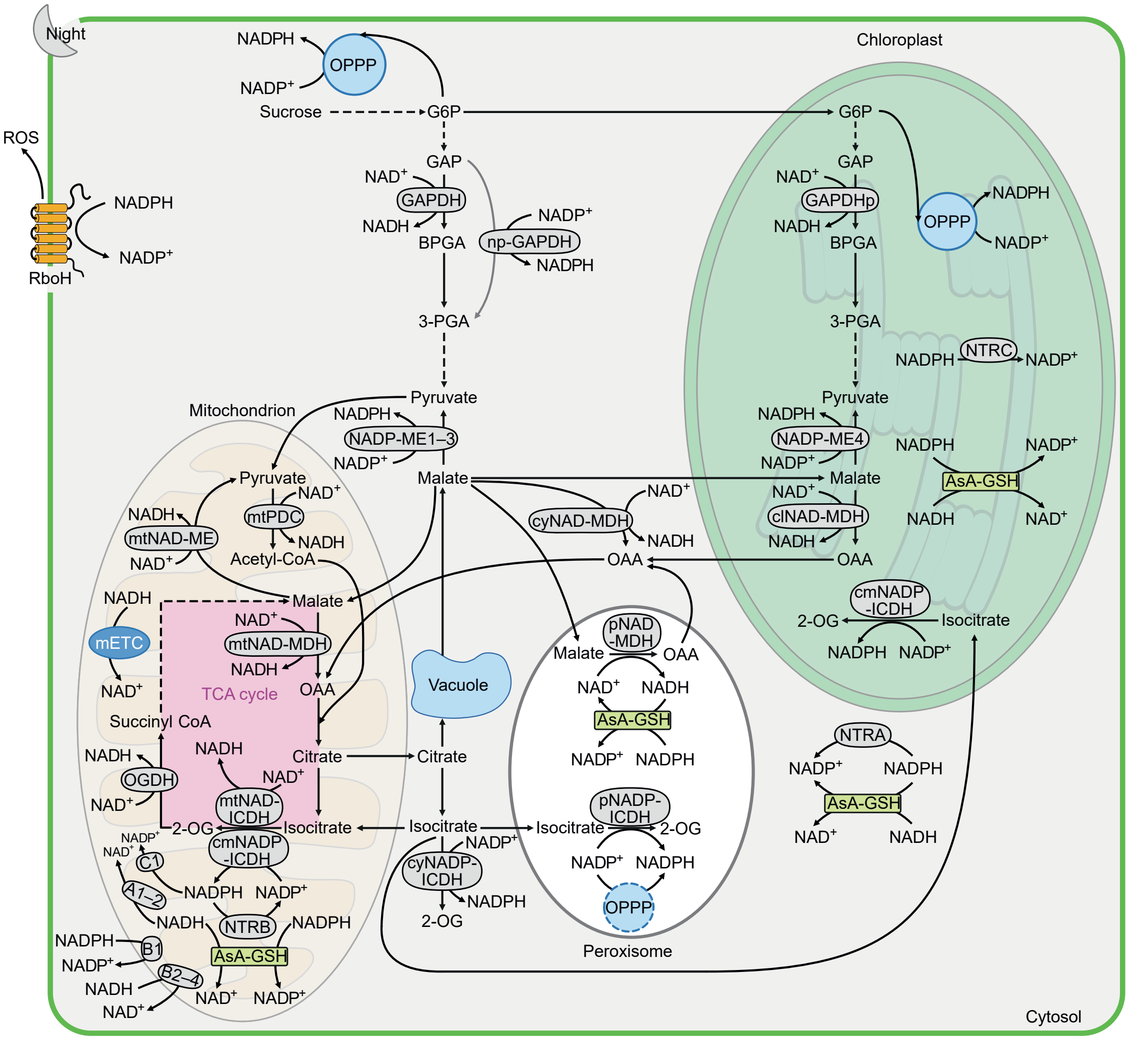

Figure 3. Major NAD(P)H production and consumption pathways in green tissues of *Arabidopsis* in the dark.

In the dark, chloroplast/cytosolic glycolysis and the TCA cycle become more active. The sucrose and malate that accumulated during the day are released from the vacuole and can serve as sources of reducing equivalents. Sucrose can supply NADPH to chloroplasts and the cytosol via OPPP and NADH (via chloroplast GAPDH [GAPDHp] and cytosolic GAPDH, respectively) via glycolysis. Whether np-GAPDH can provide cytosolic NADPH at night requires validation. Malate stored in the vacuole during the day can be released to the cytosol during the night to supply cytosolic NADPH and NADH via NADP-ME1–3 and cyNAD-MDH, respectively. In addition, cytosolic malate can enter chloroplasts, mitochondria, and peroxisomes to supply NADH and NADPH via their respective MDHs and MEs. Specifically, clNAD-MDH and NADP-ME4 provide NADH and NADPH for chloroplasts, pNAD-MDH provides NADH for peroxisomes, and mtNAD-MDH and light-inactivated mtNAD-ME provide mitochondrial NADH. In mitochondria, OGDH in the TCA cycle also provides NADH. Pyruvate generated from glycolysis and cytosolic NADP-MEs enters the mitochondria to feed the TCA cycle. Citrate is exported from the mitochondria and stored in the vacuole during the night. Isocitrate can serve as a source of NADPH in the cytosol, mitochondria, peroxisomes, and chloroplasts via their respective NADP-ICDHs. NADH and NADPH in the matrix can be consumed by NDA1-2 (A1-2) and NDC1 (C1), respectively. During the night, the incomplete OPPP pathway in peroxisomes is driven by the import of plastid G6PD1 to provide NADPH. NADPH can participate in oxidative reactions throughout the day. Cytosolic NADH can be consumed by NDB2-4 (B2-4). Cytosolic NADPH can be consumed by NDB1 (B1), RboH, and NTRA; plastid-localized NADPH can be consumed through NTRC; and mitochondrial NADPH can be consumed through NTRB. The AsA–GSH cycle can consume both NADH and NADPH in the cytosol, chloroplasts, peroxisomes, and mitochondria regardless of light conditions.

Actyl-CoA, acetyl coenzyme A; AsA–GSH, ascorbate–glutathione cycle; BPGA, 1,3-bisphosphoglycerate; cyNAD-MDH, cytosolic NAD-dependent malate dehydrogenase; clNAD-MDH, chloroplast NAD-dependent malate dehydrogenase; clNADP-MDH, chloroplast NADP-dependent malate dehydrogenase; cmNADP-ICDH, chloroplast-mitochondrial NADP-dependent isocitrate dehydrogenase; cyNADP-ICDH, cytosolic NADP-dependent isocitrate dehydrogenase; ETC, electron transport chain; FNR, ferredoxin-NADP(H) oxidoreductase; GAPDH, cytosolic NAD-specific glyceraldehyde 3-phosphate dehydrogenase; GAPDHp, plastidic NAD-specific glyceraldehyde 3-phosphate dehydrogenase; G6P, glucose 6-phosphate; GAP, glyceraldehyde 3-phosphate; mETC, mitochondrial electron transport chain; mPDC, mitochondrial pyruvate dehydrogenase complex; mNAD-ME, mitochondrial NAD-dependent malate enzyme; mtNAD-MDH, mitochondrial NAD-dependent malate dehydrogenase; mNAD-ICDH, mitochondrial NAD-dependent isocitrate dehydrogenase; np-GAPDH, non-phosphorylating glyceraldehyde 3-phosphate dehydrogenase; NADP-ME, NADP-dependent malate enzyme; NDA1-2 (A1-2), alternative NADH dehydrogenases A1-2; NDB1 (B1), alternative NADPH dehydrogenase B1; NDB2-4 (B2-4), alternative NADPH dehydrogenase B2-4; NDC1 (C1), alternative NADPH dehydrogenase C1; NTRA, NADPH-dependent thioredoxin reductase A; NTRB,

(legend continued on next page)

NADK (Figure 2). As various $NADP^+$/NADPH-dependent enzymatic reactions take place in human peroxisomes, a transporter is likely to transport cytosolic $NADP^+$ into peroxisomes, but it remains to be identified (Chornyi et al., 2020). Various studies in plants have demonstrated the existence of NADPH utilization pathways in plant mitochondria, but no plant mitochondrion-localized NADK or transporter for $NADP^+$ into mitochondria has been identified or validated (Gakière et al., 2018; Moller et al., 2020).

WHY DO ORGANISMS REQUIRE TWO DIFFERENT ENERGY CURRENCIES?

NADH is primarily involved in energy-producing catabolic reactions, whereas NADPH mainly participates in anabolic and antioxidative pathways (Oka et al., 2012; Ju et al., 2020). The two redox regulatory mechanisms of NADH and NADPH in cells are precise and non-conflicting, so how do the two mechanisms operate?

THE NADH REDOX SYSTEM

In living cells, NADH is primarily generated via catabolic reactions, including glycolysis, pyruvate oxidation, the tricarboxylic acid (TCA) cycle, fatty acid oxidation, glycine oxidation, and glutamate oxidation (Supplemental Table 1). The most important role of NADH is to provide electrons for aerobic ATP production by the mitochondrial electron transport chain (mETC) to support fundamental cellular activities (Rasmusson et al., 2008; Schertl and Braun, 2014). In the following sections, we compare NADH metabolism in plants and mammals.

NADH generation in plants

Plants use different sources of NADH in the light and dark. In the dark, malate and complex carbon molecules that accumulated during the day fuel the production of NADH. Glycolysis is a biochemical process that predominantly occurs in the dark (Figure 3). During glycolysis, glyceraldehyde 3-phosphate dehydrogenases (GAPDHs) generate NADH by catalyzing the oxidation of glyceraldehyde-3-phosphate (GAP) to 1,3-bisphosphoglycerate. Cytosolic NAD-GAPDH (encoded by *GAPC*) and plastidic NAD-GAPDH (encoded by *GAPCp*) participate in glycolysis in the cytosol and plastid, respectively (Zeng et al., 2016). In photosynthetic cells, glycolysis in chloroplasts is mainly responsible for supplying stromal ATP at night (Voon et al., 2018). In non-photosynthetic cells, glycolysis occurs in both the cytosol and plastids throughout the day; both pathways are interconnected via highly selective transporters present on the inner plastid membrane (Petersen et al., 2003; Anoman et al., 2015, 2016).

Pyruvate oxidation connects glycolysis and the TCA cycle. During this process, pyruvate is converted to acetyl-CoA by the pyruvate dehydrogenase complex (PDC) to produce CO_2 and NADH (Patel et al., 2014). Plant cells contain two PDC isoforms: a mitochondrion-localized PDC (mtPDC) and a plastid-localized PDC (plPDC). In green tissues, plPDC is active in the light and supplies acetyl-CoA for fatty acid biosynthesis, whereas mtPDC is active in the dark and feeds the TCA cycle (Budde and Randall, 1990; Tovar-Mendez et al., 2003).

Each acetyl-CoA molecule that is fed into the TCA cycle reduces three molecules of NAD^+ to NADH. The first NADH molecule is generated by isocitrate dehydrogenase (ICDH), which converts isocitrate to 2-OG and reduces $NAD(P)^+$ to NAD(P)H. In vascular plants, NAD-dependent ICDHs are strictly localized to mitochondria (Lemaitre and Hodges, 2006). The second molecule of NADH is produced by the conversion of 2-OG into succinyl-CoA via the mitochondrial multienzyme 2-oxoglutarate dehydrogenase complex (OGDH). The structure of this multienzyme is conserved across plants, mammals, and prokaryotes (Frank et al., 2007; Nemeria et al., 2014; Condori-Apfata et al., 2019). The third NADH molecule is produced by a reversible malate oxidation reaction via malate dehydrogenase (MDH). Arabidopsis has multiple NAD-MDH isoforms, including mitochondrial AtmtNAD-MDH1 and AtmtNAD-MDH2 (which function in the TCA cycle and photorespiration), three cytosolic MDHs (cyNAD-MDH1–3), and one plastidic MDH (plNAD-MDH), as well as two peroxisomal MDHs (pNAD-MDH1–2) involved in photorespiration (Selinski and Scheibe, 2019). In the light, the TCA cycle in photosynthetic cells does not operate as a cycle and does not provide a substantial amount of NADH in mitochondria as it does in the dark because the activities of mtPDC, mtNAD-ICDH, and OGDH are suppressed under illumination (Figure 4) (Sweetlove et al., 2010; Gardestrom and Igamberdiev, 2016; Fedorin et al., 2022; Igamberdiev and Bykova, 2023).

Glutamate oxidation can supply mitochondrial NADH through glutamate dehydrogenase (GDH), which reversibly deaminates glutamate, producing NAD(P)H and 2-OG. Plants contain distinct isozymes of GDH that are either NAD^+ or $NADP^+$ specific (Dubois et al., 2003; Qiu et al., 2019). Plant NAD-specific GDHs in mitochondria and NADP-specific GDHs in chloroplasts both function in nitrogen assimilation (Terce-Laforgue et al., 2004; Fontaine et al., 2012; Du et al., 2019).

Photorespiration as a major source of NADH in C3 plants

In C3 plants, during photorespiration, decarboxylation and deamination of glycine, catalyzed by glycine decarboxylase (GDC) in mitochondria, generates NADH, ammonia (NH_3), and CO_2 (Douce et al., 2001). This is the major source of NADH for the mETC during photosynthesis (Hagemann and Bauwe, 2016; Lim et al., 2020). An abundant supply of NADH from photorespiration allosterically suppresses the activities of various TCA cycle enzymes (Gardestrom and Igamberdiev, 2016). C4 plants have evolved the capacity to spatially compartmentalize initial CO_2 fixation and decarboxylation, which greatly increases the CO_2 concentration around Rubisco, thereby minimizing photorespiration and diminishing the mitochondrial NADH pool (Sage, 2016; Schulze et al., 2016). In Kranz-type C4 plants, anaplerotic CO_2 fixation occurs in mesophyll cells (MSCs), and malate decarboxylation and

NADPH-dependent thioredoxin reductase B; NTRC, NADPH-dependent thioredoxin reductase C; OGDH, 2-oxoglutarate dehydrogenase complex; OAA, oxaloacetate; OPPP, oxidative pentose phosphate pathway; pNAD-MDH, peroxisomal NAD-dependent malate dehydrogenase; pNADP-ICDH, peroxisomal NADP-dependent isocitrate dehydrogenase; RboH, respiratory burst oxidase homologs; Succinyl CoA, succinyl-coenzyme A; TCA cycle, tricarboxylic acid cycle; 3-PGA, 3-phosphoglycerate; 2-OG; 2-oxoglutarate.

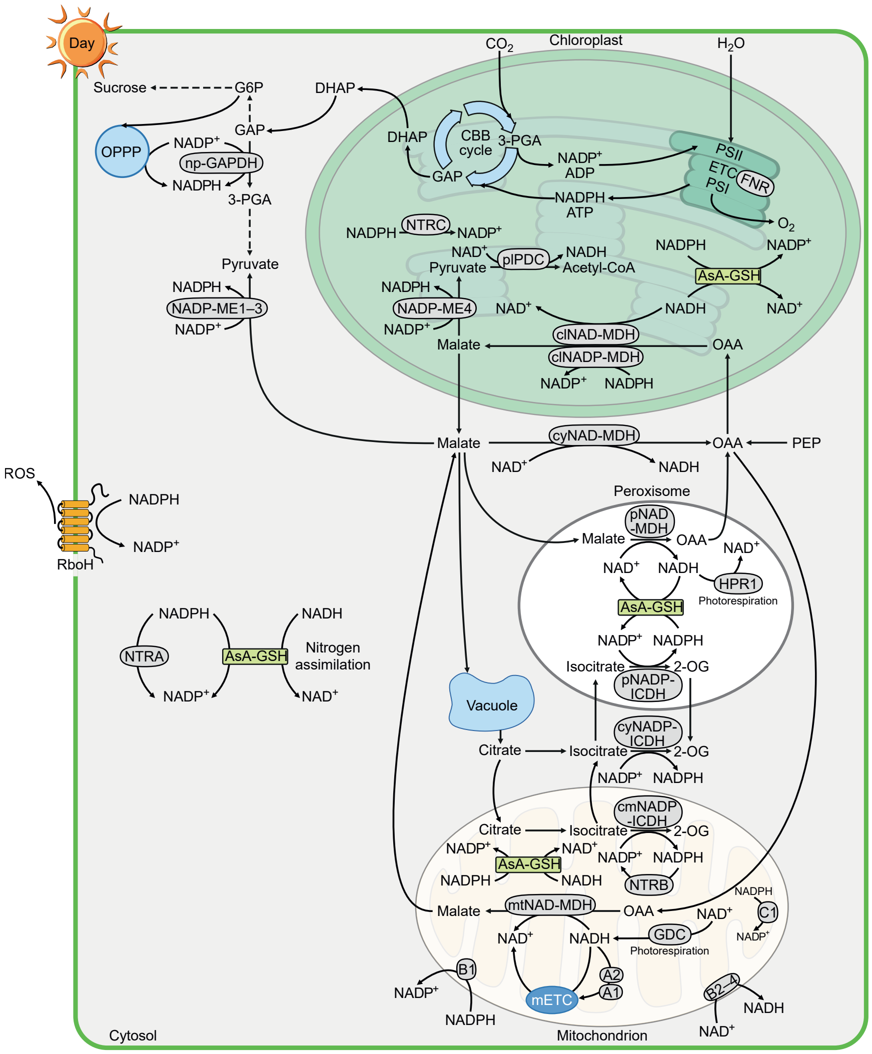

Figure 4. Major NAD(P)H production and consumption pathways in green tissues of *Arabidopsis* in the light.

Under illumination, the photosynthetic light reactions are the main source of NADPH in the chloroplasts. During this process, electrons are transferred from water molecules to reduce $NADP^+$ to NADPH via FNR, and NADPH is mainly consumed by the Calvin cycle. Malate accumulating in chloroplasts can be converted to pyruvate and provides NADPH via NADP-ME4. Pyruvate is then oxidized to acetyl-CoA and produces NADH in the light. NADH produced during this reaction can be consumed by clNAD-MDH during the conversion of OAA to malate. In the light, this step can also be catalyzed by clNADP-MDH. Excess reducing equivalents are exported from chloroplasts to the cytosol in the form of malate and triose-P; the latter is mainly used for sucrose biosynthesis. Malate can also be a source of cytosolic NADPH and NADH via the actions of NADP-ME1–3 and cyNAD-MDH, respectively. np-GAPDH and cytosolic OPPP can also supply cytosolic NADPH. The conversion of PEP into OAA in the cytosol is light dependent, offering an important source of cytosolic OAA during the day. Cytosolic OAA enters the mitochondria and is converted into malate by mtNAD-MDH. This reaction and the mETC consume large amounts of mitochondrial NADH provided by the glycine decarboxylation step of photorespiration via GDC. The excess NADH can also be consumed by NDA1-2 (A1-2), with NDA1 (A1) being significantly induced in the light. During the daytime, the tricarboxylic acid (TCA) cycle is not actually a cycle, as the activities of mtPDC, citrate synthase, NAD-ICDH, and OGDH are suppressed. Surplus mitochondrial NADH can be exported to the cytosol in the form of malate. Some cytosolic malate is imported into peroxisomes to generate NADH via pNAD-MDH and is then consumed in the hydroxypyruvate reduction step of photorespiration via HPR1. The remaining surplus cytosolic malate and synthesized sucrose are stored in the vacuole. Citrate accumulating in the vacuole at night can be released during the day via conversion to isocitrate in the cytosol and mitochondria by cyNADP-ICDH and cmNADP-ICDH, respectively, offering a supply of NADPH. Cytosolic isocitrate is transported to peroxisomes and can supply NADPH via pNADP-ICDH. In the light, cytosolic nitrogen assimilation is the major cytosolic NADH sink. Under stress conditions, the NADPH oxidase RboH can consume cytosolic NADPH. TrxRs also consume NADPH in the cytosol (NTRA), chloroplasts (NTRC), and mitochondria (NTRB). The AsA–GSH cycle can consume both NADH and NADPH in the cytosol, chloroplasts, peroxisomes, and mitochondria regardless of light conditions. In addition, NADPH in the matrix can be consumed by NDC1 (C1); cytosolic NADPH and NADH can be consumed by NDB1 (B1) and NDB2-4 (B2-4), respectively.

DHAP, dihydroxyacetone phosphate; GDC, glycine decarboxylase; HPR1, hydroxypyruvate reductase 1; PEP, phosphoenolpyruvate; plPDC, plastidic pyruvate dehydrogenase complex; PSI, photosystem I; PSII, photosystem II.

the Calvin–Benson–Bassham (CBB) cycle occur in bundle sheath cells (BSCs). In C4 plants, GDC protein is mainly expressed in BSC mitochondria, which primarily function in C1 metabolism, as photorespiration is significantly inhibited by the high CO_2 concentration resulting from the C4 mechanism (Hylton et al., 1988; Schulze et al., 2016). By contrast, the C4 plant *Bienertia sinuspersici* achieves spatial compartmentalization in a single cell using dimorphic chloroplasts (i.e., peripheral and central chloroplasts) (Offermann et al., 2011, 2015).

C4 plants are divided into the NADP-malic enzyme (NADP-ME), NAD-malic enzyme (NAD-ME), and phosphoenolpyruvate carboxykinase (PEPCK) subtypes, but no pure PEPCK C4 species has been identified to date. In the NAD-ME subtype, Asp generated from OAA in MSC chloroplasts is transported to mitochondria in the BSCs and converted back to OAA via AspAT and then to malate by mtNAD-MDH. The malate is then decarboxylated by NAD-ME to release CO_2 and NADH (Rao and Dixon, 2016). Light significantly induces *NAD-MEα* expression in NAD-ME C4 but not C3 *Cleome* species (Hudig et al., 2022). By contrast, in the C3 plant Arabidopsis, the activities and expression of two mitochondrial *NAD-ME* genes (*mtNAD-ME1* and *mtNAD-ME2*) decrease during the day (Tronconi et al., 2008). The high expression of *mtNAD-ME2* could represent an adaptation of NAD-ME C4 plants. For example, *mtNAD-ME2* is expressed at higher levels in *B. sinuspersici* than in Arabidopsis,

and this gene is also expressed at much higher levels in NAD-ME C4 grasses than in C3 grasses (Rao and Dixon, 2016; Watson-Lazowski et al., 2018; Han et al., 2023). Unlike *mtNAD-ME*, *mtNAD-MDH* transcription did not markedly differ between C3 and NAD-ME C4 *Cleome* species, and mtNAD-MDH enzyme activity was high in both species, suggesting that the capacity of mtNAD-MDH is sufficient in both C3 and C4 *Cleome* species (Brautigam et al., 2011; Sommer et al., 2012).

NADH generation from the glyoxylate cycle of plants

In addition to the main TCA cycle in the mitochondrial matrix, plants also employ a unique cycle that bypasses the TCA cycle known as the glyoxylate cycle, which occurs in glyoxysomes (Graham, 2001; Pracharoenwattana et al., 2005; Kunze and Hartig, 2013). Glyoxysomes are specialized microbodies found in plant cells, particularly in oil-rich germinating seeds such as in soybean (*Glycine max*), Arabidopsis, and sunflower (*Helianthus annuus*). Germinating oilseeds mainly rely on the degradation of their oil reserves as a source of carbon backbones and energy. The core glyoxylate cycle involves the conversion of acetyl-CoA to succinate, with each cycle producing one NADH molecule that is generated via the oxidation of malate by glyoxysomal NAD-MDH. In leaves, glyoxysomes disappear and peroxisomes appear when photosynthesis is initiated (Oikawa et al., 2019; De Bellis et al., 2020). During the phototrophic stage, fatty acids are oxidized via β-oxidation in peroxisomes to generate NADH for plant growth under limited carbon conditions (Goepfert and Poirier, 2007; Yu et al., 2019).

NADH generation in mammalian cells

In mammals, glycolysis occurs only in the cytosol and generates NADH via a single GAPDH (Tristan et al., 2011; Ikeda et al., 2012). Subsequently, pyruvate, the end product of glycolysis, is transported into the mitochondria and oxidized by PDC to generate NADH and acetyl-CoA, the latter entering the TCA cycle. As in plants, each turn of the TCA cycle in mammals produces three NADH molecules, which are produced sequentially by ICDH, OGDH, and MDH. Humans contain three ICDHs: hICDH1–hICDH3. hICDH3 generates NADH in mitochondria, and the other two enzymes generate NADPH (Reitman and Yan, 2010). The human MDH isoforms hMDH1 and hMDH2 are both NAD^+ specific, and, while mitochondrial hMDH2 is involved in the TCA cycle, hMDH1 localizes to the cytosol and peroxisomes (McCue and Finzel, 2022). Malate is also decarboxylated via NAD(P)-ME to provide NAD(P)H. Human cells contain three malic enzymes—hNADP-ME1, hNADP-ME2, and hNAD(P)-ME3—with hNADP-ME1 localized to the cytosol and the other two enzymes localized to mitochondria. hNAD(P)-ME3 participates in the TCA cycle and generates NADH, and it also produces NADPH for lipid biosynthesis and glutathione reduction (Hsieh et al., 2019).

In mammals, fatty acid β-oxidation occurs and generates NADH in both mitochondria and peroxisomes (Poirier et al., 2006). As mammals only obtain sugars from their diet, when cells contain insufficient glucose levels, fat reserves are converted into fatty acids via lipolysis. The resulting fatty acids undergo β-oxidation in the mitochondria to produce NADH, thereby supporting ATP production via the mETC (Elmadfa and Kornsteiner, 2009). The oxidation of amino acids also provides NADH in mammalian cells. In mammals, glycine oxidation via GDC functions in mitochondrial folate metabolism (Pai et al., 2015). Unlike plant GDHs, mammalian mitochondrial GDHs use both NAD^+ and $NADP^+$, allowing mitochondria to use glutamate as an NADH source for the mETC when required (Bunik et al., 2016; Plaitakis et al., 2017).

Major sources of NADH for mitochondrial ATP production in mammals versus plants

In mammals, the TCA cycle is the main source of NADH for ATP production in mitochondria. In plants, however, the sources of mitochondrial NADH vary in different tissues, developmental stages, light conditions, and photosynthetic types. During oilseed germination, β-oxidation and the glyoxylate cycle convert stored lipids into succinate, which is transported to mitochondria for NADH production (Voon and Lim, 2019). In the photosynthetic tissues of C3 plants, the glycine decarboxylation step of photorespiration supplies a large amount of NADH to mitochondria, exceeding the NADH-consuming capacity of the mETC, and the surplus NADH is exported to the cytosol in the form of malate (Lim et al., 2020). Hence, in the photosynthetic tissues of C3 plants, photorespiration and the TCA cycle are the major sources of NADH for ATP production in mitochondria during the day and night, respectively. In the non-photosynthetic tissues of plants and in the photosynthetic tissues of C4 plants, the TCA cycle represents the major source of fuel for ATP production in mitochondria during the day.

NADH consumption in plants

Under aerobic conditions, NADH produced in mammal and plant mitochondria is mainly consumed through the mETC (Efremov and Sazanov, 2012; Lapuente-Brun et al., 2013; Sazanov, 2014). Here, NADH is consumed by complex I to generate the proton motive force needed to produce ATP through ATP synthase. However, the system is more sophisticated in plants. Alternative oxidase (AOX) and alternative NAD(P)H dehydrogenases (NDs) in plant mitochondria only transfer electrons without proton translocation and thus do not directly contribute to oxidative phosphorylation. AOX directly transfers electrons from ubiquinone to O_2 to generate water. NDs located on the inner or outer surface of the inner mitochondrial membrane transfer electrons from NAD(P)H in the mitochondrial intermembrane space or matrix to ubiquinone (UQ), which is then transferred to complexes III–IV or AOX (Rasmusson et al., 2008; McDonald et al., 2009; Antos-Krzeminska and Jarmuszkiewicz, 2019). Arabidopsis contains seven ND isoforms divided into three subfamilies: NDA, NDB, and NDC. NADH-dependent NDA1-2 (NDin) are located on the inner surface of the mitochondrial inner membrane. Light significantly induces the transcription of *AtNDA1* and it might function in the dissipation of mitochondrial NADH produced by photorespiration (Elhafez et al., 2006). NADPH-dependent NDB1 (NPDex) and NADH-dependent NDB2-NDB4 (NDex) are located on the outer surface of the inner mitochondrial membrane (Schertl and Braun, 2014; Moller et al., 2021). AtNDBs have a low pH optimum, and the activities of AtNDB1 and AtNDB2 were strongly and moderately stimulated by Ca^{2+}, respectively (Geisler et al., 2007). Some stress conditions, such as hypoxia and ammonium and salt treatments, increase cytosolic Ca^{2+} level, which then activates Ca^{2+}-dependent AtNDB1 and AtNDB2, thereby alleviating ROS generation by consuming cytosolic NAD

(P)H (Igamberdiev and Hill, 2018; Rasmusson et al., 2020; Bachani et al., 2022). The activity of NADPH-dependent NDC1, located on the inner surface of the mitochondrial inner membrane, is also stimulated by Ca^{2+} and low pH (Rasmusson and Moller, 1991). Under certain stress conditions, concurrent upregulation of ND and AOX genes was observed, which helps plants to decrease UQ pool reduction and prevent excessive generation of ROS (Moller, 2001; Van Aken et al., 2009; Vanlerberghe, 2013; Sweetman et al., 2019; Rasmusson et al., 2020).

Fermentation is a common anaerobic pathway that consumes NADH without ATP production. When cellular O_2 concentrations are below a given threshold, pyruvate in the plant cytosol is reduced to lactate by lactate dehydrogenase (LDH) or converted to acetaldehyde by PDC, followed by further reduction to ethanol by acetaldehyde dehydrogenase (Dolferus et al., 2008; Wei et al., 2009).

In plant nitrogen assimilation, nitrate (NO_3^-) reduction is catalyzed by nitrate reductase (NR), the activity of which is light activated in photosynthetic tissues (Riens and Heldt, 1992). NR is present in the plant cytosol and uses NAD(P)H as its electron donor. In most vascular plants, NR uses NADH as an electron donor, whereas a bi-specific form of NR that uses NADH/NADPH is present in monocotyledons and some dicotyledons (e.g., soybean); NR in mosses and fungi uses NADPH as the electron donor (Tischner and Kaiser, 2007). In vascular plants, nitrite (NO_2^-), a product of nitrate reduction, is transported to plastids, converted to ammonium (NH_4^+) by nitrite reductase (NIR), and participates in the GS–GOGAT cycle (Takahashi et al., 2001; Gupta et al., 2022). In the GS–GOGAT cycle, Gln is converted to Glu by GOGAT, which uses reduced ferredoxin (Fd_{red}) as the electron donor in photosynthetic tissues and NADH as the electron donor in non-photosynthetic tissues (Kojima et al., 2014).

In C3 plants, toxic glyoxylate is generated during photorespiration in peroxisomes and is rapidly converted to glycine via glyoxylate aminotransferase. Glyoxylate transported to the cytosol and chloroplasts is detoxified by NAD(P)H-dependent glyoxylate reductase (GR). In Arabidopsis and rice, there are two GR isoforms, cytosolic GR and chloroplast GR, and both prefer NADPH rather than NADH as a cofactor (Simpson et al., 2008; Zhang et al., 2020b). Peroxisomal glycine is transported and converted to serine in mitochondria, which is exported and converted to hydroxypyruvate in peroxisomes and further reduced to glycerate using NADH via hydroxypyruvate reductase (HPR). Glycerate is further converted to 3-phosphoglyceric acid (3-PGA) in chloroplasts to complete the photorespiration cycle. Arabidopsis has three HPR isoforms: peroxisome-localized NADH-dependent AtHPR1, cytosolic NADPH-dependent AtHPR2, and chloroplast-localized NADPH-dependent AtHPR3 (Timm et al., 2011). We suggest that the preferences of enzymes with NAD(P)H dual specificity have evolved as an adaptation to the prevailing concentrations of NADH and NADPH in their respective subcellular compartments. In both the cytosol and chloroplasts, where NADPH levels are higher than those of NADH, NADPH is preferred by GRs and by AtHPR2 and AtHPR3 (Gakière et al., 2018).

NADH consumption in mammals

In mammals, NADH is mainly consumed through the mETC under aerobic conditions via complex I. Under anerobic conditions, mammalian cells perform lactate fermentation to recycle NAD^+ via cytosolic LDH. In addition to functioning in the cytosolic lactate fermentation pathway, LDH is also found in mammalian peroxisomes, where it utilizes peroxisomal NADH to reduce pyruvate (Schueren et al., 2014). In mammals, glyoxylate is also toxic and is reduced to glycolate by the dual-localized bifunctional enzyme glyoxylate reductase/HPR using NAD(P)H in mitochondria and the cytosol. In peroxisomes, glyoxylate is metabolized to glycine by alanine/glyoxylate aminotransferase (Booth et al., 2006; Belostotsky et al., 2012; Salido et al., 2012; Garrelfs et al., 2024).

THE NADPH REDOX SYSTEM

NADPH is a key electron donor for ROS detoxification and a variety of anabolic pathways, including fatty acid and nucleic acid biosynthesis, the CBB cycle, carotenoid biosynthesis, and proline biosynthesis (Aghdam et al., 2020; Moller et al., 2020). A major difference between mammalian cells and plant cells is that plant cells contain chloroplasts, which have a profound influence on the plant NADPH regulatory system (Supplemental Table 2).

NADPH in plant chloroplasts/plastids

In photosynthetic tissues under illumination, photosynthesis is the primary source of NADPH production in chloroplasts (Figure 4). Linear electron flow (LEF; powered by sunlight) is the key electron flow in the photosystems, in which electrons are transferred from water to reduce $NADP^+$ to NADPH by ferredoxin-NADP(H) oxidoreductase (FNR) (Morigasaki et al., 1990; Hanke et al., 2005; Mulo, 2011). By contrast, in non-photosynthetic tissues and in photosynthetic tissues in the dark, OPPP is the major source of NADPH in plastids (Figure 3). Plants have two complete OPPPs: one in the cytosol and one in plastids. Both pathways convert one molecule of glucose-6-phosphate to ribulose-5-phosphate to generate two molecules of NADPH via glucose-6-phosphate dehydrogenase (G6PD) and 6-phosphogluconate dehydrogenase (6PGD). There are six G6PDs in Arabidopsis, namely plastid-localized G6PD1–4 and cytosolic G6PD5 and G6PD6 (Wakao and Benning, 2005). The Arabidopsis genome encodes three 6PGDs: PGD1 and PGD3 localize in the cytosol and plastids, and PGD2 localizes in the cytosol and peroxisomes (Holscher et al., 2016).

Plant chloroplasts/plastids also contain other NADPH-producing enzymes, including NADP-ICDH, NADP-MDH, and NADP-ME. Four NADP-ICDH isozymes were identified in Arabidopsis, including one isoenzyme localized to peroxisomes, one to the cytosol, one to mitochondria, and one to chloroplasts. Notably, the mitochondrion-localized and chloroplast-localized NADP-ICDH isozymes are encoded by the same gene (Hodges et al., 2003; Leterrier et al., 2016).

NADP-MDH is localized to the chloroplast (Ocheretina et al., 2000) (Figure 4). In C3 plants, the enzymatic activity of NADP-MDH is light activated and contributes to the export of surplus reducing equivalents from the chloroplast to the cytosol via malate export (Foyer et al., 2009; Lim et al., 2020; Yokochi et al., 2021). During the evolution of NADP-ME C4 plants, the enzyme activity and transcript abundance of NADP-MDH have been

shaped to support C4 photosynthesis. In this type of photosynthesis, OAA is converted to malate by NADP-MDH in MSCs, which is then transported to BSCs and decarboxylated via NADP-ME to release CO_2 via NADP-ME (Rao and Dixon, 2016). Although the *NADP-MDH* gene exists as a single copy in both C3 and NADP-ME C4 *Flaveria* species, the enzyme activity and transcript abundance of NADP-MDH are much higher in the latter. In addition, light significantly induced the transcription of *NADP-MDH* in C4 *Flaveria* species and maize (*Zea mays*) but had no effect on this gene in C3 *Flaveria* species (Metzler et al., 1989; McGonigle and Nelson, 1995; Lyu et al., 2025).

Similar to *NADP-MDH*, the transcription of *NADP-ME* in NADP-ME C4 *Flaveria* species is induced by light, and its enzyme activity is much higher in NADP-ME C4 *Flaveria* species than in C3 *Flaveria* species (Lyu et al., 2025). Arabidopsis contains only one chloroplast NADP-ME isoform (NADP-ME4). By contrast, in maize, the original *non-C4-NADP-ME* gene was duplicated and an additional *C4-NADP-ME* gene evolved (Wheeler et al., 2005; Alvarez et al., 2013; Böhm et al., 2025). The enzymatic activity of maize C4 NADP-ME is much higher than that of maize non-C4 NADP-ME and AtNADP-ME4 (Maier et al., 2011). In addition, maize *C4-NADP-ME* is expressed at much higher levels in leaves compared to *non-C4-NADP-ME*, and UV treatment significantly increased the expression of maize *C4-NADP-ME* but had little effect on non-C4 and cytosolic *NADP-ME*s (Alvarez et al., 2013).

In chloroplasts, NADPH generated via the light reactions of photosynthesis is mainly used in the CBB cycle via NADP-specific GAPDH (Marri et al., 2009; Zeng et al., 2016). This pool of NADPH is also used by thioredoxins, as well as for lipid and chlorophyll biosynthesis (Mulo, 2011). In non-photosynthetic plastids, root FNR reduces Fd using NADPH derived from the OPPP to drive various metabolic pathways (Hachiya et al., 2016; Guan et al., 2018). In vascular plants, nitrite reduction during nitrogen assimilation also occurs in plastids/chloroplasts, a process catalyzed by NIR using Fd_{red} as an electron donor in photosynthetic chloroplasts and NADPH as an electron donor in non-photosynthetic plastids (Joy and Hageman, 1966; Takahashi et al., 2001).

NADPH is also involved in ROS detoxification, which is important for defense responses and signal transduction. In chloroplasts, ROS is mainly produced via the Mehler reaction, which generates superoxide (O_2^-) from O_2 in photosystem I; the resulting ROS is detoxified via the ascorbate–glutathione (AsA–GSH) cycle and the thioredoxin (Trx) system, both of which use NADPH as an electron donor. In the AsA–GSH cycle, glutathione reductase (GTR) uses NADPH as a source of reducing equivalents to catalyze the reduction of oxidized glutathione (GSSG) to reduced glutathione (GSH) (Noctor et al., 2012). GSH is then converted back to GSSG by dehydroascorbate reductases (DHARs). Electrons from hydrogen peroxide (H_2O_2) are then dissipated by the oxidation of ascorbate (AsA) to monodehydroascorbate (MDHA) via ascorbate peroxidase. AsA is recycled either from dehydroascorbate via DHAR or from MDHA via NAD(P)H-dependent monodehydroascorbate reductase (MDAR) (Noctor et al., 2000). Arabidopsis contains two GTR isoforms: AtGTR1 localizes in the cytosol, nucleus, and peroxisomes and AtGTR2 is targeted to plastids and mitochondria (Foyer and Noctor, 2011; Marty et al., 2019). Arabidopsis also contains five MDAR isoforms: cytosolic/peroxisomal AtMDAR1, cytosolic AtMDAR2 and AtMDAR3, peroxisomal AtMDAR4, and chloroplastic/mitochondrial AtMDAR5. AtMDARs have different NAD(P)H preferences, with AtMDAR1 and AtMDAR5 preferring NADH and AtMDAR2 preferring NADPH (Vanacker et al., 2018; Tanaka et al., 2021). In the Trx system, peroxiredoxins, a class of cysteine-dependent peroxidases, are reduced by NADPH oxidation via thioredoxin reductase (TrxR) and Trx (Hebbelmann et al., 2012). In Arabidopsis and rice, three NADPH-dependent TrxRs (NTRs) with disulfide-bond reductase activity have been reported: NTRA, the major cytosolic NTR; NTRB, the major mitochondrion-localized NTR, sharing a redundant function in the cytosol and mitochondria with NTRA; and chloroplast-localized NTRC (Cha et al., 2015).

NADPH in the cytosol

NADPH levels in eukaryotic cells are usually lower in the cytosol than in organelles (Tao et al., 2017; Lim et al., 2020). As mentioned above, the OPPP is an essential source of NADPH in plant plastids and cytosol; in mammals, the OPPP is only found in the cytosol (TeSlaa et al., 2023). In addition to the OPPP, cytosolic NADPH is also produced via several NADPH-generating enzymes, including non-phosphorylating GAPDH (np-GAPDH), NADP-ICDH, and NADP-ME. np-GAPDH, an enzyme mainly found in autotrophic eukaryotes, catalyzes the oxidation of GAP to 3-PGA in the cytosol and generates one molecule of NADPH (Bustos and Iglesias, 2003; Wieloch, 2021). Cytosolic NADP-ICDH activity has been detected in all Arabidopsis tissues, with high activity in leaves (Mhamdi et al., 2010). Arabidopsis contain three cytosolic NADP-ME isoforms, AtNADP-ME1–3, and only *AtNADP-ME2* is continuously expressed in leaves and roots (Wheeler et al., 2005). In humans, hICDH1 and hNADP-ME1 generate NADPH in the cytosol, and the latter plays a role in cytosolic lipogenesis (Reitman and Yan, 2010; Hsieh et al., 2014).

In plants, cytosolic NADPH provides reducing power for members of the respiratory burst oxidase homolog (RboH) family. RboHs are a class of transmembrane proteins that mediate the transfer of electrons from intracellular NADPH to extracellular O_2 to give O_2^-, which is subsequently catabolized to H_2O_2 by superoxide dismutase (Kaur and Pati, 2016; Foyer and Noctor, 2020). In mammals, the transmembrane enzymes NADPH oxidases catalyze electron transfer from cytosolic NADPH across the cell membrane to generate H_2O_2 (Torres and Dangl, 2005; Nazari et al., 2023). The H_2O_2 is detoxified by the cytosolic AsA–GSH cycle and the Trx systems using NADPH as a cofactor. In Arabidopsis, AtGTR1 and NTRA consume NADPH and function in the cytosolic AsA–GSH cycle and the Trx system, respectively. In humans, a single gene, *hGTR*, encodes both cytosolic and mitochondrion-localized isoenzymes that function in the AsA–GSH cycle in each compartment (Kelner and Montoya, 2000). The two major TrxR isoforms in humans, cytosolic TrxR1 and mitochondrial TrxR2, function in redox signaling in the corresponding organelles (Misevičienė et al., 2022). Notably, in plants, cytosolic NAD(P)H can also be consumed by NDBs located on the outer surface of the inner mitochondrial membrane, thereby participating in cytosolic redox metabolism (Rasmusson et al., 2008, 2020).

NADPH in mitochondria

The mitochondrion is an important site for ROS production during cellular oxidative phosphorylation in both plant and mammalian cells (Lenaz, 2001; Moller, 2001; Navrot et al., 2006). In humans, hNADK2 generates $NADP^+$ in mitochondria, which is then converted to NADPH by mitochondrion-localized NADPH-generating enzymes including hICDH2, hNADP-ME2, hNAD(P)-ME3, and proton-translocating transhydrogenase (Reitman and Yan, 2010; Ohashi et al., 2012; Hsieh et al., 2014, 2019; Francisco et al., 2022). Proton-translocating transhydrogenase, also known as nicotinamide nucleotide transhydrogenase (NNT), catalyzes the reversible transfer of hydride from NADH to $NADP^+$ coupled to inward proton translocation. This enzyme is only found in the inner membranes of animal mitochondria and the plasma membranes of some prokaryotes (Jackson, 2012; Kastaniotis et al., 2017). In mammals, the electrochemical proton gradient (Δp) generated by respiration is mainly consumed by the NNT forward reaction. As a result, the pH of the mammalian mitochondrial matrix is 0.2 units lower than that of the plant mitochondrial matrix, which makes the [NADPH][NAD^+]/[$NADP^+$][NADH] ratio two orders of magnitude greater in mammalian than in plant mitochondria (Jackson, 2003; Shen et al., 2013; Gakière et al., 2018; Zou et al., 2018). In mammals, $NADP^+$ produced by mitochondrial NADK and NADH generated by mitochondrial β-oxidation provide sufficient substrate for NNT, and the NADPH produced by NNT is consumed during fatty acid biosynthesis (Kastaniotis et al., 2017). As fatty acid biosynthesis in plants occurs in plastids and beta-oxidation occurs in peroxisomes, NNT is not needed to provide large amounts of NADPH in plant mitochondria and there is no evidence that it is present (Bykova et al., 1999; Moller et al., 2020).

NADPH in the plant mitochondrial matrix is mainly consumed by NDC and the NADPH-dependent ROS detoxification system, NTRB-Trx system, and AsA–GSH cycle via GTR2 (Moller and Rasmusson, 1998; Moller, 2001; Rasmusson et al., 2008, 2020; Cha et al., 2015; Marty et al., 2019). Although the activity of the mitochondrion-localized NADPH-generating enzyme NADP-ICDH has been detected in Arabidopsis and pea (*Pisum sativum*) (Rasmusson and Moller, 1990; Igamberdiev and Gardestrom, 2003; Leterrier et al., 2016), no mitochondrial NADK has been identified in plants. $NADP^+$ was shown to be transported across the inner membrane of the plant mitochondrion (Bykova and Moller, 2001), but this result has not been verified, and there are no known plant mitochondrial membrane carriers for $NADP^+$ (Moller et al., 2020). Therefore, the origin of plant mitochondrial $NADP^+$ requires further investigation.

NADPH in peroxisomes

Peroxisomes are highly dynamic organelles with pivotal roles in various metabolic pathways, such as fatty acid oxidation and glyoxylate metabolism. Many enzymes involved in these pathways generate distinct types of ROS. In mammalian peroxisomes, H_2O_2 is mainly metabolized into H_2O by catalase or transported into the cytosol by peroxisomal membrane protein 2, thus maintaining redox balance in peroxisomes (Rokka et al., 2009; Fransen et al., 2012). Therefore, in mammals, peroxisomal NADPH mainly functions in anabolic pathways. In humans, NADP-dependent hICDH1, which localizes to both the cytosol and peroxisomes, provides NADPH for the biosynthesis of fat and cholesterol in peroxisomes (Reitman and Yan, 2010; Van Veldhoven, 2010). However, human cells lack NADK in their peroxisomes and thus require a transporter to transport cytosolic $NADP^+$ into peroxisomes (Chornyi et al., 2020). The identity of this transporter remains unknown, and peroxisomal NADPH metabolism in mammalian cells also needs to be elucidated.

By contrast, NADK is present in plant peroxisomes, and the pathways for NADPH production in plant peroxisomes are more numerous. Plant peroxisomal NADP-ICDH (pNADP-ICDH) produces NADPH in peroxisomes (Leterrier et al., 2016). An incomplete OPPP present in plant peroxisomes also generates NADPH. In Arabidopsis, PGD2 is present in peroxisomes. However, Arabidopsis G6PD isoforms do not carry distinct C-terminal peroxisomal targeting signal 1 (PTS1) or N-terminal PTS2 motifs, and the interaction between G6PD4 and G6PD1 facilitates the import of G6PD1 into peroxisomes to complete the OPPP (Corpas et al., 1998; Meyer et al., 2011). Plant peroxisomal NADPH participates in ROS detoxification via the peroxisomal AsA–GSH cycle (Del Rio and Lopez-Huertas, 2016; Marty et al., 2019).

PYRIDINE NUCLEOTIDE POOLS AND RATIOS IN PLANTS

NAD(H) and NADP(H) pools and the ratios of reduced/oxidized forms vary among different tissues and compartments and change dynamically under different conditions. *In vivo*, most NAD(P)H molecules are protein-bound, and the concentration of free NAD(P)H is relatively low (Agius et al., 2001; Kasimova et al., 2006; Smith et al., 2021). In plants, the levels of free and total NADH in cytosol under darkness were estimated to be 0. 5 and 18 μM, respectively, and their levels in mitochondria were 70 and 190 μM, respectively, which were higher than the free NADH levels in human cell cytosol (0.12 μM) and mitochondria (30 μM) (Heineke et al., 1991; Igamberdiev and Gardestrom, 2003; Kasimova et al., 2006; Zhao et al., 2011). The combined levels of free $NADP^+$ (38 μM) and NADPH (150 μM) in plant mitochondria and cytosol were also higher than that of human cell mitochondria (0.19 μM for $NADP^+$ and 37 μM for NADPH) and cytosol (0.1 μM for $NADP^+$ and 3.1 μM for NADPH) (Heineke et al., 1991; Tao et al., 2017; Zou et al., 2018).

Early studies employed *in vitro* methods to measure the subcellular concentrations of pyridine nucleotides in plant organelles rapidly fractionated from protoplasts. Under illumination, free NADPH and $NADP^+$ levels are higher in spinach leaf chloroplasts than in the cytosol and mitochondria, and the NADPH/$NADP^+$ ratio in the stroma under light (0.5) is a double of the ratio in the dark (0.23) (Heineke et al., 1991). The NADH level in mitochondria is higher than in chloroplasts and cytosol, and the NADH/NAD^+ ratio in the cytosol is much lower than that in mitochondria and chloroplasts (Heineke et al., 1991; Szal et al., 2008). Illumination of pea leaves increased mitochondrial total NADH level from 75 to 455 μM and the NADH/NAD^+ ratio from 0.05 to 0.29, and, in cytosol, total NADH level increased from 18 to 55 μM and the ratio from 0.03 to 0.1. These changes

disappeared when photorespiration was inhibited by saturating CO_2 level (Igamberdiev and Gardestrom, 2003). Similarly, the $NADH/NAD^+$ ratio in the mitochondria of barley (*Hordeum vulgare*) protoplasts dropped from 0.22 to 0.07 when photorespiration was inhibited by high CO_2 concentration under illumination (Wigge and Krömer, 1993). In plant and mammalian cells, the ratio of $NADH/NAD^+$ is usually lower than that of $NADPH/NADP^+$ (Wigge and Krömer, 1993; Zou et al., 2018). These two coenzyme couples have a marked difference in their reduction levels in plant matrix, as they are not in thermodynamic equilibrium but in a kinetic steady state (Moller et al., 2020).

The recent application of fluorescent biosensors in plants has enabled more sensitive and efficient monitoring of the dynamic changes in free pyridine nucleotide contents in different subcellular compartments *in planta* (Smith et al., 2021). An increase in the $NADH/NAD^+$ ratio upon illumination was observed in Arabidopsis stroma using the *in planta* biosensor SoNar; this increase disappeared when photorespiration was inhibited. This analysis also revealed that the changes in the cytosolic $NADH/NAD^+$ ratio were dependent on light intensity, with the ratio increasing at 296 mmol m^{-2} s^{-1} and decreasing at 40 mmol m^{-2} s^{-1} (Lim et al., 2020). $NADH/NAD^+$ ratios and NADPH levels are differentially regulated in plants (Lim et al., 2020; Liu et al., 2022). Notably, 3 min of illumination increased the stromal NADPH level and $NADH/NAD^+$ ratio. Both readings dropped after the light was turned off; the $NADH/NAD^+$ ratio dropped to below basal levels, and the NADPH level only decreased during the first minute of darkness and subsequently stabilized above the basal level (Lim et al., 2020). These differences are due to the differential regulation of NAD(P)-MDH activities. While stromal NADP-MDH is activated by light and inactivated in the dark, the activation of NAD-MDHs in various organelles occurs independently of light. Mitochondrial NAD-MDH, due to its equilibrium properties and $NADH/NAD^+$ buffering ability, rapidly recycles NAD^+ from NADH, thereby alleviating the inhibition of mtPDC and GDC activities under high rates of respiration and photorespiration (Hagedorn et al., 2004; Bykova et al., 2014; Igamberdiev et al., 2014). These MDHs allow the rapid transport and storage of surplus reducing equivalents generated from photosynthesis and photorespiration in the form of malate, which accumulates in the vacuole during the day, and supplies NADH to various compartments in the dark (Figures 3 and 4).

CONCLUDING REMARKS AND PERSPECTIVES

The NADH and NADPH systems are robust and complex and have evolved to become increasingly sophisticated. Due to the endosymbiotic origin of chloroplasts, the functions of organelles in plant cells are more specialized than those in mammalian cells. In mammalian cells, mitochondria are enriched in both NADH and NADPH metabolic pathways. By contrast, plant mitochondria serve as powerhouses of the cell by consuming reductants harvested by chloroplasts and providing the cytosol with ATP and are therefore enriched in NADH metabolic pathways (Gardestrom and Igamberdiev, 2016). Plant chloroplasts capture solar energy and are involved in various anabolic pathways, resulting in the marked enrichment of NADPH-related metabolism.

More research on plant NAD(H) and NADP(H) metabolism is needed. The identity of chloroplast NAD^+ transporter is still unclear. Homologs of AtNDT1, AtNDT2, and AtPXN can be searched among chloroplast transmembrane proteins and their NAD^+ transport capacity can be examined. The origin of $NADP^+$ in plant mitochondria remains to be elucidated, and the $NADP^+$ transporter in the plant mitochondrial carrier family remains to be revealed.

Reduction and oxidation of pyridine nucleotides consume or release protons and these processes could affect subcellular pH. Their effect on pH is determined by the net release or uptake of protons in conjunction with concomitant reactions (Igamberdiev and Kleczkowski, 2019; Smith et al., 2021). For example, NADPH produced by Fd-FNR is consumed in the CBB cycle. Hence, the contribution to pH stat via proton release and consumption in the pyridine nucleotide redox reactions warrants further studies.

Under sufficient light, photosynthetic plant cells produce a surplus of reducing equivalents that will generate ROS, deplete $NADP^+$, and impede LEF. This depletion of $NADP^+$ particularly affects C3 photosynthesis, as tremendous NADH generated by photorespiration indirectly restricts stromal $NADP^+$ regeneration from the malate valve (Lim et al., 2020). Therefore, inhibiting photorespiration by introducing photorespiratory bypasses (Xin et al., 2015; Shen et al., 2019) and enhancing NAD(P)H metabolism could improve energy efficiency of C3 plants. For example, overexpression of *AtPAP2* improved photosynthetic efficiency and productivity by enhancing chloroplast and mitochondrial activities, optimizing NAD(P)H metabolism, and increasing the $NADP^+/NADPH$ ratio (Sun et al., 2012; Liang et al., 2015; Voon et al., 2018; Cai et al., 2022). Other strategies such as optimizing NAD(P)H metabolic fluxes, enhancing enzyme stability, and reducing ATP costs, are potential pathways for improving energy-use efficiency in plants.

FUNDING

This research was funded by the Hong Kong Research Grants Council General Research Fund (17102322), a Biotechnology and Biological Sciences Research Council grant (BB/W018748/1), the Area of Excellence Scheme (AoE/M-403/16), and the Innovation and Technology Fund (Funding Support to State Key Laboratory of Agrobiotechnology) of the Hong Kong Special Administrative Region, China. This project was also supported by the National Natural Science Foundation of China (32070394)

ACKNOWLEDGMENTS

No conflict of interest is declared.

AUTHOR CONTRIBUTIONS

D.L. prepared the first draft, figures, and tables. B.L.L. and M.G. reviewed and edited the manuscript, figures, and tables. B.L.L. supervised the project. All authors have read and approved the final version of the manuscript.

SUPPLEMENTAL INFORMATION

Supplemental information is available at *Molecular Plant Online*.

Received: November 3, 2024
Revised: March 31, 2025
Accepted: May 9, 2025
Published: May 13, 2025

REFERENCES

Aghdam, M.S., Palma, J.M., and Corpas, F.J. (2020). NADPH as a quality footprinting in horticultural crops marketability. Trends Food Sci. Technol. **103**:152–161. https://doi.org/10.1016/j.tifs.2020.07.002.

Agius, S., Rasmusson, A., and Moller, I. (2001). NAD(P) turnover in plant mitochondria. Aust. J. Plant Physiol. **28**:461–470. https://doi.org/10.1071/PP00155.

Agrimi, G., Russo, A., Scarcia, P., and Palmieri, F. (2012a). The human gene *SLC25A17* encodes a peroxisomal transporter of coenzyme A, FAD and NAD^+. Biochem. J. **443**:241–247. https://doi.org/10.1042/BJ20111420.

Agrimi, G., Russo, A., Pierri, C.L., and Palmieri, F. (2012b). The peroxisomal NAD^+ carrier of *Arabidopsis thaliana* transports coenzyme A and its derivatives. J. Bioenerg. Biomembr. **44**:333–340. https://doi.org/10.1007/s10863-012-9445-0.

Ahmad, Z., Bashir, K., Matsui, A., Tanaka, M., Sasaki, R., Oikawa, A., Hirai, M.Y., Chaomurilege, Zu, Y., Zu, Y., Kawai-Yamada, M., et al. (2021). Overexpression of *nicotinamidase 3* (*NIC3*) gene and the exogenous application of nicotinic acid (NA) enhance drought tolerance and increase biomass in Arabidopsis. Plant Mol. Biol. **107**:63–84. https://doi.org/10.1007/s11103-021-01179-z.

Akashi, H., and Gojobori, T. (2002). Metabolic efficiency and amino acid composition in the proteomes of *Escherichia coli* and *Bacillus subtilis*. Proc. Natl. Acad. Sci. USA **99**:3695–3700. https://doi.org/10.1073/pnas.062526999.

Alvarez, C.E., Saigo, M., Margarit, E., Andreo, C.S., and Drincovich, M. F. (2013). Kinetics and functional diversity among the five members of the NADP-malic enzyme family from Zea mays, a C4 species. Photosynth. Res. **115**:65–80. https://doi.org/10.1007/s11120-013-9839-9.

Anoman, A.D., Flores-Tornero, M., Rosa-Telléz, S., Muñoz-Bertomeu, J., Segura, J., and Ros, R. (2016). The specific role of plastidial glycolysis in photosynthetic and heterotrophic cells under scrutiny through the study of glyceraldehyde-3-phosphate dehydrogenase. Plant Signal. Behav. **11**:e1128614. https://doi.org/10.1080/15592324.2015.1128614.

Anoman, A.D., Muñoz-Bertomeu, J., Rosa-Téllez, S., Flores-Tornero, M., Serrano, R., Bueso, E., Fernie, A.R., Segura, J., and Ros, R. (2015). Plastidial glycolytic glyceraldehyde-3-phosphate dehydrogenase is an important determinant in the carbon and nitrogen metabolism of heterotrophic cells in Arabidopsis. Plant Physiol. **169**:1619–1637. https://doi.org/10.1104/pp.15.00696.

Antos-Krzeminska, N., and Jarmuszkiewicz, W. (2019). Alternative Type II NAD(P)H Dehydrogenases in the Mitochondria of Protists and Fungi. Protist **170**:21–37. https://doi.org/10.1016/j.protis.2018.11.001.

Ashihara, H., and Deng, W.W. (2012). Pyridine metabolism in tea plants: salvage, conjugate formation and catabolism. J. Plant Res. **125**:781–791. https://doi.org/10.1007/s10265-012-0490-x.

Bachani, J., Mahanty, A., Aftab, T., and Kumar, K. (2022). Insight into calcium signalling in salt stress response. South Afr. J. Bot. **151**:1–8. https://doi.org/10.1016/j.sajb.2022.09.033.

Badawy, A.A.B. (2017). Kynurenine pathway of tryptophan metabolism: Regulatory and functional aspects. Int. J. Tryptophan Res. **10**:1178646917691938. https://doi.org/10.1177/1178646917691938.

Bahn, A., Hagos, Y., Reuter, S., Balen, D., Brzica, H., Krick, W., Burckhardt, B.C., Sabolic, I., and Burckhardt, G. (2008). Identification of a new urate and high affinity nicotinate transporter, hOAT10 (SLC22A13). J. Biol. Chem. **283**:16332–16341. https://doi.org/10.1074/jbc.M800737200.

Bai, P., and Cantó, C. (2012). The role of PARP-1 and PARP-2 enzymes in metabolic regulation and disease. Cell Metab. **16**:290–295. https://doi.org/10.1016/j.cmet.2012.06.016.

Belostotsky, R., Pitt, J.J., and Frishberg, Y. (2012). Primary hyperoxaluria type III-a model for studying perturbations in glyoxylate metabolism. J. Mol. Med. **90**:1497–1504. https://doi.org/10.1007/s00109-012-0930-z.

Berger, F., Lau, C., Dahlmann, M., and Ziegler, M. (2005). Subcellular compartmentation and differential catalytic properties of the three human nicotinamide mononucleotide adenylyltransferase isoforms. J. Biol. Chem. **280**:36334–36341. https://doi.org/10.1074/jbc.M508660200.

Bernhardt, K., Wilkinson, S., Weber, A.p.m., and Linka, N. (2012). A peroxisomal carrier delivers NAD^+ and contributes to optimal fatty acid degradation during storage oil mobilization. Plant J. **69**:1–13. https://doi.org/10.1111/j.1365-313X.2011.04775.x.

Berrin, J.G., Pierrugues, O., Brutesco, C., Alonso, B., Montillet, J.L., Roby, D., and Kazmaier, M. (2005). Stress induces the expression of *AtNADK-1*, a gene encoding a NAD(H) kinase in *Arabidopsis thaliana*. Mol. Genet. Genomics. **273**:10–19. https://doi.org/10.1007/s00438-005-1113-1.

Bjorn, L.O., and Govindjee. (2009). The evolution of photosynthesis and chloroplasts. Curr. Sci. (Bangalore) **96**:1466–1474.

Boehler, C., Gauthier, L.R., Mortusewicz, O., Biard, D.S., Saliou, J.M., Bresson, A., Sanglier-Cianferani, S., Smith, S., Schreiber, V., Boussin, F., and Dantzer, F. (2011). Poly(ADP-ribose) polymerase 3 (PARP3), a newcomer in cellular response to DNA damage and mitotic progression. Proc. Natl. Acad. Sci. USA **108**:2783–2788. https://doi.org/10.1073/pnas.1016574108.

Böhm, J.M., Willms, S., Ferrao, O., Buitrago-Arango, M., Hüdig, M., Poschmann, G., Fazelnia, N., Nagel-Steger, L., Klinke, S., Drakonaki, A., et al. (2025). From dimer to tetramer: the evolutionary trajectory of C4 photosynthetic NADP-ME oligomeric state in Poaceae. Preprint at bioRxiv. https://doi.org/10.1101/2025.01.05.631420.

Booth, M.P.S., Conners, R., Rumsby, G., and Brady, R.L. (2006). Structural basis of substrate specificity in human glyoxylate reductase/hydroxypyruvate reductase. J. Mol. Biol. **360**:178–189. https://doi.org/10.1016/j.jmb.2006.05.018.

Bossi, R.T., Negri, A., Tedeschi, G., and Mattevi, A. (2002). Structure of FAD-bound L-aspartate oxidase: Insight into substrate specificity and catalysis. Biochemistry **41**:3018–3024. https://doi.org/10.1021/bi015939r.

Braidy, N., Berg, J., Clement, J., Khorshidi, F., Poljak, A., Jayasena, T., Grant, R., and Sachdev, P. (2019). Role of nicotinamide adenine dinucleotide and related precursors as therapeutic targets for age-related degenerative diseases: Rationale, biochemistry, pharmacokinetics, and outcomes. Antioxid. Redox Signal. **30**:251–294. https://doi.org/10.1089/ars.2017.7269.

Brautigam, A., Kajala, K., Wullenweber, J., Sommer, M., Gagneul, D., Weber, K.L., Carr, K.M., Gowik, U., Mass, J., Lercher, M.J., et al. (2011). An mRNA blueprint for C4 photosynthesis derived from comparative transcriptomics of closely related C3 and C4 species. Plant Physiol **155**:142–156. https://doi.org/10.1104/pp.110.159442.

Budde, R.J., and Randall, D.D. (1990). Pea leaf mitochondrial pyruvate dehydrogenase complex is inactivated in vivo in a light-dependent manner. Proc. Natl. Acad. Sci. USA **87**:673–676. https://doi.org/10.1073/pnas.87.2.673.

Bunik, V., Artiukhov, A., Aleshin, V., and Mkrtchyan, G. (2016). Multiple forms of glutamate dehydrogenase in animals: structural determinants

and physiological implications. Biology **5**:53. https://doi.org/10.3390/biology5040053.

Bustos, D.M., and Iglesias, A.A. (2003). Phosphorylated non-phosphorylating glyceraldehyde-3-phosphate dehydrogenase from heterotrophic cells of wheat interacts with 14-3-3 proteins. Plant Physiol. **133**:2081–2088. https://doi.org/10.1104/pp.103.030981.

Bykova, N.V., and Møller, I.M. (2001). Involvement of matrix NADP turnover in the oxidation of NAD-linked substrates by pea leaf mitochondria. Physiol. Plant. **111**:448–456. https://doi.org/10.1034/j.1399-3054.2001.1110404.x.

Bykova, N.V., Moller, I.M., Gardestrom, P., and Igamberdiev, A.U. (2014). The function of glycine decarboxylase complex is optimized to maintain high photorespiratory flux via buffering of its reaction products. Mitochondrion **19**:357–364. https://doi.org/10.1016/j.mito.2014.01.001.

Bykova, N.V., Rasmusson, A.G., Igamberdiev, A.U., Gardeström, P., and Møller, I.M. (1999). Two separate transhydrogenase activities are present in plant mitochondria. Biochem. Biophys. Res. Commun. **265**:106–111. https://doi.org/10.1006/bbrc.1999.1627.

Cai, Y., Zhai, Z., Blanford, J., Liu, H., Shi, H., Schwender, J., Xu, C., and Shanklin, J. (2022). Purple acid phosphatase2 stimulates a futile cycle of lipid synthesis and degradation, and mitigates the negative growth effects of triacylglycerol accumulation in vegetative tissues. New Phytol. **236**:1128–1139. https://doi.org/10.1111/nph.18392.

Cha, J.Y., Barman, D.N., Kim, M.G., and Kim, W.Y. (2015). Stress defense mechanisms of NADPH-dependent thioredoxin reductases (NTRs) in plants. Plant Signal. Behav. **10**:e1017698. https://doi.org/10.1080/15592324.2015.1017698.

Chandel, N.S. (2021). NADPH-The forgotten reducing equivalent. Cold Spring Harb. Perspect. Biol. **13**:a040550. https://doi.org/10.1101/cshperspect.a040550.

de Souza Chaves, I., Feitosa-Araújo, E., Florian, A., Medeiros, D.B., da Fonseca-Pereira, P., Charton, L., Heyneke, E., Apfata, J.A.C., Pires, M.V., Mettler-Altmann, T., et al. (2019). The mitochondrial NAD^+ transporter (NDT1) plays important roles in cellular NAD^+ homeostasis in *Arabidopsis thaliana*. Plant J. **100**:487–504. https://doi.org/10.1111/tpj.14452.

Chen, M., Yuan, L., Chen, B., Chang, H., Luo, J., Zhang, H., Chen, Z., Kong, J., Yi, Y., Bai, M., et al. (2025). SLC29A1 and SLC29A2 are human nicotinamide cell membrane transporters. Nat. Commun. **16**:1181. https://doi.org/10.1038/s41467-025-56402-y.

Chini, C.C.S., Zeidler, J.D., Kashyap, S., Warner, G., and Chini, E.N. (2021). Evolving concepts in NAD^+ metabolism. Cell Metab. **33**:1076–1087. https://doi.org/10.1016/j.cmet.2021.04.003.

Chornyi, S., IJlst, L., van Roermund, C.W.T., Wanders, R.J.A., and Waterham, H.R. (2020). Peroxisomal metabolite and cofactor transport in humans. Front. Cell Dev. Biol. **8**:613892. https://doi.org/10.3389/fcell.2020.613892.

Condori-Apfata, J.A., Batista-Silva, W., Medeiros, D.B., Vargas, J.R., Valente, L.M.L., Heyneke, E., Pérez-Diaz, J.L., Fernie, A.R., Araújo, W.L., and Nunes-Nesi, A. (2019). The Arabidopsis E_1 subunit of the 2-oxoglutarate dehydrogenase complex modulates plant growth and seed production. Plant Mol. Biol. **101**:183–202. https://doi.org/10.1007/s11103-019-00900-3.

Corpas, F.J., Barroso, J.B., Sandalio, L.M., Distefano, S., Palma, J.M., Lupiáñez, J.A., and Del Río, L.A. (1998). A dehydrogenase-mediated recycling system of NADPH in plant peroxisomes. Biochem. J. **330**:777–784. https://doi.org/10.1042/bj3300777.

Covarrubias, A.J., Perrone, R., Grozio, A., and Verdin, E. (2021). NAD^+ metabolism and its roles in cellular processes during ageing. Nat. Rev. Mol. Cell Biol. **22**:119–141. https://doi.org/10.1038/s41580-020-00313-x.

De Bellis, L., Luvisi, A., and Alpi, A. (2020). Aconitase: To be or not to be inside plant glyoxysomes, that is the question. Biology **9**:162. https://doi.org/10.3390/biology9070162.

Del Rio, L.A., and Lopez-Huertas, E. (2016). ROS generation in peroxisomes and its role in cell signaling. Plant Cell Physiol. **57**:1364–1376. https://doi.org/10.1093/pcp/pcw076.

Dell'Aglio, E., Giustini, C., Kraut, A., Couté, Y., Costa, A., Decros, G., Gibon, Y., Mazars, C., Matringe, M., Finazzi, G., and Curien, G. (2019). Identification of the Arabidopsis calmodulin-dependent NAD^+ kinase that sustains the elicitor-induced oxidative burst. Plant Physiol. **181**:1449–1458. https://doi.org/10.1104/pp.19.00912.

Dolferus, R., Wolansky, M., Carroll, R., Miyashita, Y., Ismond, K., and Good, A. (2008). Functional analysis of lactate dehydrogenase during hypoxic stress in Arabidopsis. Funct. Plant Biol. **35**:131–140. https://doi.org/10.1071/FP07228.

Douce, R., and Neuburger, M. (1989). The uniqueness of plant mitochondria. Annu. Rev. Plant Physiol. Plant Mol. Biol. **40**:371–414. https://doi.org/10.1146/annurev.pp.40.060189.002103.

Douce, R., Bourguignon, J., Neuburger, M., and Rébeillé, F. (2001). The glycine decarboxylase system: a fascinating complex. Trends Plant Sci. **6**:167–176. https://doi.org/10.1016/S1360-1385(01)01892-1.

Dragicevic, M., Simonovic, A., Bogdanovic, M., Subotic, A., Ghalawenji, N., Dragicevic, I., and Todorovic, S. (2016). Differential regulation of GS-GOGAT gene expression by plant growth regulators in Arabidopsis seedlings. Arch. Biol. Sci. **68**:399–404. https://doi.org/10.2298/abs150608087d.

Du, C.Q., Lin, J.Z., Dong, L.A., Liu, C., Tang, D.Y., Yan, L., Chen, M.D., Liu, S., and Liu, X.M. (2019). Overexpression of an NADP(H)-dependent glutamate dehydrogenase gene, TrGDH, from Trichurus improves nitrogen assimilation, growth status and grain weight per plant in rice. Breed. Sci. **69**:429–438. https://doi.org/10.1270/jsbbs.19014.

Dubois, F., Tercé-Laforgue, T., Gonzalez-Moro, M.-B., Estavillo, J.-M., Sangwan, R., Gallais, A., and Hirel, B. (2003). Glutamate dehydrogenase in plants: Is there a new story for an old enzyme? Plant Physiol. Biochem. **41**:565–576. https://doi.org/10.1016/s0981-9428(03)00075-5.

Efremov, R.G., and Sazanov, L.A. (2012). The coupling mechanism of respiratory complex I-a structural and evolutionary perspective. Biochim. Biophys. Acta **1817**:1785–1795. https://doi.org/10.1016/j.bbabio.2012.02.015.

Elhafez, D., Murcha, M.W., Clifton, R., Soole, K.L., Day, D.A., and Whelan, J. (2006). Characterization of mitochondrial alternative NAD(P)H dehydrogenases in Arabidopsis: intraorganelle location and expression. Plant Cell Physiol. **47**:43–54. https://doi.org/10.1093/pcp/pci221.

Elmadfa, I., and Kornsteiner, M. (2009). Fats and fatty acid requirements for adults. Ann. Nutr. Metab. **55**:56–75. https://doi.org/10.1159/000228996.

Fedorin, D.N., Eprintsev, A.T., and Igamberdiev, A.U. (2022). Light Regulation of Tricarboxylic Acid Cycle Isoenzymes in Plants. Russ. J. Plant Physiol. **69**:110–596. https://doi.org/10.1134/s1021443722060048.

Fisher, D.J., Fernández, R.E., and Maurelli, A.T. (2014). *Chlamydia trachomatis* Transports NAD via the Npt1 ATP/ADP Translocase. J. Bacteriol. **196**:2323. https://doi.org/10.1128/jb.01662-14.

Fjeld, C.C., Birdsong, W.T., and Goodman, R.H. (2003). Differential binding of NAD^+ and NADH allows the transcriptional corepressor carboxyl-terminal binding protein to serve as a metabolic sensor. Proc. Natl. Acad. Sci. USA **100**:9202–9207. https://doi.org/10.1073/pnas.1633591100.

Fontaine, J.X., Tercé-Laforgue, T., Armengaud, P., Clément, G., Renou, J.P., Pelletier, S., Catterou, M., Azzopardi, M., Gibon, Y.,

Lea, P.J., et al. (2012). Characterization of a NADH-dependent glutamate dehydrogenase mutant of Arabidopsis demonstrates the key role of this enzyme in root carbon and nitrogen metabolism. Plant Cell 24:4044–4065. https://doi.org/10.1105/tpc.112.103689.

Foyer, C.H., and Noctor, G. (2011). Ascorbate and glutathione: The heart of the redox hub. Plant Physiol. **155**:2–18. https://doi.org/10.1104/pp.110.167569.

Foyer, C.H., and Noctor, G. (2020). Redox Homeostasis and Signaling in a Higher-CO_2 World. Annu. Rev. Plant Biol. **71**:157–182. https://doi.org/10.1146/annurev-arplant-050718-095955.

Foyer, C.H., Bloom, A.J., Queval, G., and Noctor, G. (2009). Photorespiratory metabolism: genes, mutants, energetics, and redox signaling. Annu. Rev. Plant Biol. **60**:455–484. https://doi.org/10.1146/annurev.arplant.043008.091948.

Francisco, A., Figueira, T.R., and Castilho, R.F. (2022). Mitochondrial $NAD(P)^+$ Transhydrogenase: From Molecular Features to Physiology and Disease. Antioxid. Redox Signal. **36**:864–884. https://doi.org/10.1089/ars.2021.0111.

Frank, R.A.W., Price, A.J., Northrop, F.D., Perham, R.N., and Luisi, B. F. (2007). Crystal structure of the E1 component of the *Escherichia coli* 2-oxoglutarate dehydrogenase multienzyme complex. J. Mol. Biol. **368**:639–651. https://doi.org/10.1016/j.jmb.2007.01.080.

Fransen, M., Nordgren, M., Wang, B., and Apanasets, O. (2012). Role of peroxisomes in ROS/RNS-metabolism: implications for human disease. Biochim. Biophys. Acta **1822**:1363–1373. https://doi.org/10.1016/j.bbadis.2011.12.001.

Fricker, R.A., Green, E.L., Jenkins, S.I., and Griffin, S.M. (2018). The influence of nicotinamide on health and disease in the central nervous system. Int. J. Tryptophan Res. **11**:1178646918776658. https://doi.org/10.1177/1178646918776658.

Gakière, B., Hao, J., de Bont, L., Pétriacq, P., Nunes-Nesi, A., and Fernie, A.R. (2018). NAD^+ biosynthesis and signaling in plants. Crit. Rev. Plant Sci. **37**:259–307. https://doi.org/10.1080/07352689.2018.1505591.

Ganapathy, V., Gopal, E., Miyauchi, S., and Prasad, P.D. (2005). Biological functions of SLC5A8, a candidate tumour suppressor. Biochem. Soc. Trans. **33**:237–240. https://doi.org/10.1042/BST0330237.

Gardestrom, P., and Igamberdiev, A.U. (2016). The origin of cytosolic ATP in photosynthetic cells. Physiol Plant **157**:367–379. https://doi.org/10.1111/ppl.12455.

Gardner, C.D., Hartle, J.C., Garrett, R.D., Offringa, L.C., and Wasserman, A.S. (2019). Maximizing the intersection of human health and the health of the environment with regard to the amount and type of protein produced and consumed in the United States. Nutr. Rev. **77**:197–215. https://doi.org/10.1093/nutrit/nuy073.

Garrelfs, S.F., Chornyi, S., Te Brinke, H., Ruiter, J., Groothoff, J., and Wanders, R.J.A. (2024). Glyoxylate reductase: Definitive identification in human liver mitochondria, its importance for the compartment-specific detoxification of glyoxylate. J. Inherit. Metab. Dis. **47**:280–288. https://doi.org/10.1002/jimd.12711.

Geisler, D.A., Broselid, C., Hederstedt, L., and Rasmusson, A.G. (2007). Ca^{2+}-binding and Ca^{2+}-independent respiratory NADH and NADPH dehydrogenases of *Arabidopsis thaliana*. J. Biol. Chem. **282**:28455–28464. https://doi.org/10.1074/jbc.M704674200.

Gerdes, S.Y., Kurnasov, O.V., Shatalin, K., Polanuyer, B., Sloutsky, R., Vonstein, V., Overbeek, R., and Osterman, A.L. (2006). Comparative genomics of NAD biosynthesis in cyanobacteria. J. Bacteriol. **188**:3012–3023. https://doi.org/10.1128/JB.188.8.3012-3023.2006.

Gerdes, S.Y., Scholle, M.D., D'Souza, M., Bernal, A., Baev, M.V., Farrell, M., Kurnasov, O.V., Daugherty, M.D., Mseeh, F., Polanuyer, B.M., et al. (2002). From genetic footprinting to antimicrobial drug targets: Examples in cofactor biosynthetic pathways. J. Bacteriol. **184**:4555–4572. https://doi.org/10.1128/JB.184.16.4555-4572.2002.

Goepfert, S., and Poirier, Y. (2007). Beta-oxidation in fatty acid degradation and beyond. Curr. Opin. Plant Biol. **10**:245–251. https://doi.org/10.1016/j.pbi.2007.04.007.

Graham, P.J.E.a.I.A. (2001). Re-examining the role of the glyoxylate cycle in oilseeds. Trends Plant Sci. **6**:72–77. https://doi.org/10.1016/S1360-1385(00)01835-5.

Grozio, A., Mills, K.F., Yoshino, J., Bruzzone, S., Sociali, G., Tokizane, K., Lei, H.C., Cunningham, R., Sasaki, Y., Migaud, M.E., and Imai, S. I. (2019). Slc12a8 is a nicotinamide mononucleotide transporter. Nat. Metab. **1**:47–57. https://doi.org/10.1038/s42255-018-0009-4.

Guan, X., Chen, S., Voon, C.P., Wong, K.B., Tikkanen, M., and Lim, B.L. (2018). FdC1 and leaf-type ferredoxins channel electrons from Photosystem I to different downstream electron acceptors. Front. Plant Sci. **9**:410. https://doi.org/10.3389/fpls.2018.00410.

Gupta, K.J., Kaladhar, V.C., Fitzpatrick, T.B., Fernie, A.R., Møller, I.M., and Loake, G.J. (2022). Nitric oxide regulation of plant metabolism. Mol. Plant **15**:228–242. https://doi.org/10.1016/j.molp.2021.12.012.

Hachiya, T., Ueda, N., Kitagawa, M., Hanke, G., Suzuki, A., Hase, T., and Sakakibara, H. (2016). Arabidopsis root-type ferredoxin:NADP(H) oxidoreductase 2 is involved in detoxification of nitrite in roots. Plant Cell Physiol. **57**:2440–2450. https://doi.org/10.1093/pcp/pcw158.

Hagedorn, P.H., Flyvbjerg, H., and Møller, I.M. (2004). Modelling NADH turnover in plant mitochondria. Physiol. Plant. **120**:370–385. https://doi.org/10.1111/j.0031-9317.2003.00252.x.

Hagemann, M., and Bauwe, H. (2016). Photorespiration and the potential to improve photosynthesis. Curr. Opin. Chem. Biol. **35**:109–116. https://doi.org/10.1016/j.cbpa.2016.09.014.

Han, S.Y., Kim, W.Y., Kim, J.S., and Hwang, I. (2023). Comparative transcriptomics reveals the role of altered energy metabolism in the establishment of single-cell C(4) photosynthesis in Bienertia sinuspersici. Front. Plant Sci. **14**:1202521. https://doi.org/10.3389/fpls.2023.1202521.

Hanke, G.T., Okutani, S., Satomi, Y., Takao, T., Suzuki, A., and Hase, T. (2005). Multiple iso-proteins of FNR in Arabidopsis_ evidence for different contributions to chloroplast function and nitrogen assimilation. Plant Cell Environ. **28**:1146–1157. https://doi.org/10.1111/j.1365-3040.2005.01352.x.

Hashida, S.N., Takahashi, H., Kawai-Yamada, M., and Uchimiya, H. (2007). *Arabidopsis thaliana* nicotinate/nicotinamide mononucleotide adenyltransferase (AtNMNAT) is required for pollen tube growth. Plant J. **49**:694–703. https://doi.org/10.1111/j.1365-313X.2006.02989.x.

Hebbelmann, I., Selinski, J., Wehmeyer, C., Goss, T., Voss, I., Mulo, P., Kangasjärvi, S., Aro, E.M., Oelze, M.L., Dietz, K.J., et al. (2012). Multiple strategies to prevent oxidative stress in Arabidopsis plants lacking the malate valve enzyme NADP-malate dehydrogenase. J. Exp. Bot. **63**:1445–1459. https://doi.org/10.1093/jxb/err386.

Heineke, D., Riens, B., Grosse, H., Hoferichter, P., Peter, U., Flügge, U. I., and Heldt, H.W. (1991). Redox transfer across the inner chloroplast envelope membrane. Plant Physiol. **95**:1131–1137. https://doi.org/10.1104/pp.95.4.1131.

Hodges, M., Flesch, V., Gálvez, S., and Bismuth, E. (2003). Higher plant $NADP^+$-dependent isocitrate dehydrogenases, ammonium assimilation and NADPH production. Plant Physiol. Biochem. **41**:577–585. https://doi.org/10.1016/s0981-9428(03)00062-7.

Holscher, C., Lutterbey, M.C., Lansing, H., Meyer, T., Fischer, K., and von Schaewen, A. (2016). Defects in peroxisomal 6-phosphogluconate dehydrogenase isoform PGD2 prevent gametophytic interaction in

Arabidopsis thaliana. Plant Physiol **171**:192–205. https://doi.org/10.1104/pp.15.01301.

Hsieh, J.Y., Shih, W.T., Kuo, Y.H., Liu, G.Y., and Hung, H.C. (2019). Functional roles of metabolic intermediates in regulating the human mitochondrial NAD(P)$^+$-dependent malic enzyme. Sci. Rep. **9**:9081. https://doi.org/10.1038/s41598-019-45282-0.

Hsieh, J.Y., Li, S.Y., Chen, M.C., Yang, P.C., Chen, H.Y., Chan, N.L., Liu, J.H., and Hung, H.C. (2014). Structural characteristics of the nonallosteric human cytosolic malic enzyme. Biochim. Biophys. Acta **1844**:1773–1783. https://doi.org/10.1016/j.bbapap.2014.06.019.

Hudig, M., Tronconi, M.A., Zubimendi, J.P., Sage, T.L., Poschmann, G., Bickel, D., Gohlke, H., and Maurino, V.G. (2022). Respiratory and C4-photosynthetic NAD-malic enzyme coexist in bundle sheath cell mitochondria and evolved via association of differentially adapted subunits. Plant Cell **34**:597–615. https://doi.org/10.1093/plcell/koab265.

Hunt, L., Lerner, F., and Ziegler, M. (2004). NAD-new roles in signalling and gene regulation in plants. New Phytol. **163**:31–44. https://doi.org/10.1111/j.1469-8137.2004.01087.x.

Hylton, C.M., Rawsthorne, S., Smith, a.m., Jones, D.A., and Woolhouse, H.W. (1988). Glycine decarboxylase is confined to the bundle-sheath cells of leaves of C3−C4 intermediate species. Planta **175**:452–459. https://doi.org/10.1007/BF00393064.

Igamberdiev, A.U., and Gardeström, P. (2003). Regulation of NAD- and NADP-dependent isocitrate dehydrogenases by reduction levels of pyridine nucleotides in mitochondria and cytosol of pea leaves. Biochim. Biophys. Acta **1606**:117–125. https://doi.org/10.1016/s0005-2728(03)00106-3.

Igamberdiev, A.U., and Hill, R.D. (2018). Elevation of cytosolic Ca^{2+} in response to energy deficiency in plants: the general mechanism of adaptation to low oxygen stress. Biochem. J. **475**:1411–1425. https://doi.org/10.1042/BCJ20180169.

Igamberdiev, A.U., and Kleczkowski, L.A. (2019). Thermodynamic buffering, stable non-equilibrium and establishment of the computable structure of plant metabolism. Prog. Biophys. Mol. Biol. **146**:23–36. https://doi.org/10.1016/j.pbiomolbio.2018.11.005.

Igamberdiev, A.U., and Bykova, N.V. (2023). Mitochondria in photosynthetic cells: Coordinating redox control and energy balance. Plant Physiol. **191**:2104–2119. https://doi.org/10.1093/plphys/kiac541.

Igamberdiev, A.U., Lernmark, U., and Gardestrom, P. (2014). Activity of the mitochondrial pyruvate dehydrogenase complex in plants is stimulated in the presence of malate. Mitochondrion 19 Pt **B**:184–190. https://doi.org/10.1016/j.mito.2014.04.006.

Ikeda, Y., Yamaji, R., Irie, K., Kioka, N., and Murakami, A. (2012). Glyceraldehyde-3-phosphate dehydrogenase regulates cyclooxygenase-2 expression by targeting mRNA stability. Arch. Biochem. Biophys. **528**:141–147. https://doi.org/10.1016/j.abb.2012.09.004.

Ishikawa, Y., and Kawai-Yamada, M. (2019). Physiological significance of NAD kinases in cyanobacteria. Front. Plant Sci. **10**:847. https://doi.org/10.3389/fpls.2019.00847.

Jackson, J.B. (2003). Proton translocation by transhydrogenase. FEBS Lett. **545**:18–24. https://doi.org/10.1016/s0014-5793(03)00388-0.

Jackson, J.B. (2012). A review of the binding-change mechanism for proton-translocating transhydrogenase. Biochim. Biophys. Acta **1817**:1839–1846. https://doi.org/10.1016/j.bbabio.2012.04.006.

Jiang, R., Jin, B., Sun, Y., Chen, Z., Wan, D., Feng, J., Ying, L., Peng, C., and Gu, L. (2025). SLC7A5 regulates tryptophan uptake and PD-L1 expression levels via the kynurenine pathway in ovarian cancer. Oncol. Lett. **29**:26. https://doi.org/10.3892/ol.2024.14772.

Jiang, Y., Wang, D., Yao, Y., Eubel, H., Künzler, P., Møller, I.M., and Xu, D. (2021). MULocDeep: A deep-learning framework for protein subcellular and suborganellar localization prediction with residue-level interpretation. Comput. Struct. Biotechnol. J. **19**:4825–4839. https://doi.org/10.1016/j.csbj.2021.08.027.

Joy, K.W., and Hageman, R.H. (1966). The purification and properties of nitrite reductase from higher plants, and its dependence on ferredoxin. Biochem. J. **100**:263–273. https://doi.org/10.1042/bj1000263.

Ju, H.Q., Lin, J.F., Tian, T., Xie, D., and Xu, R.H. (2020). NADPH homeostasis in cancer: functions, mechanisms and therapeutic implications. Signal Transduct. Target. Ther. **5**:231. https://doi.org/10.1038/s41392-020-00326-0.

Kasimova, M.R., Grigiene, J., Krab, K., Hagedorn, P.H., Flyvbjerg, H., Andersen, P.E., and Møller, I.M. (2006). The free NADH concentration is kept constant in plant mitochondria under different metabolic conditions. Plant Cell **18**:688–698. https://doi.org/10.1105/tpc.105.039354.

Kastaniotis, A.J., Autio, K.J., Kerätär, J.M., Monteuuis, G., Mäkelä, a. m., Nair, R.R., Pietikäinen, L.P., Shvetsova, A., Chen, Z., and Hiltunen, J.K. (2017). Mitochondrial fatty acid synthesis, fatty acids and mitochondrial physiology. Biochim. Biophys. Acta. Mol. Cell Biol. Lipids **1862**:39–48. https://doi.org/10.1016/j.bbalip.2016.08.011.

Katahira, R., and Ashihara, H. (2009). Profiles of the biosynthesis and metabolism of pyridine nucleotides in potatoes (*Solanum tuberosum* L.). Planta **231**:35–45. https://doi.org/10.1007/s00425-009-1023-2.

Katoh, A., Uenohara, K., Akita, M., and Hashimoto, T. (2006). Early steps in the biosynthesis of NAD in Arabidopsis start with aspartate and occur in the plastid. Plant Physiol. **141**:851–857. https://doi.org/10.1104/pp.106.081091.

Kaur, G., and Pati, P.K. (2016). Analysis of cis-acting regulatory elements of Respiratory burst oxidase homolog (*Rboh*) gene families in Arabidopsis and rice provides clues for their diverse functions. Comput. Biol. Chem. **62**:104–118. https://doi.org/10.1016/j.compbiolchem.2016.04.002.

Kawai, S., Mori, S., Mukai, T., Hashimoto, W., and Murata, K. (2001a). Molecular characterization of *Escherichia coli* NAD kinase. Eur. J. Biochem. **268**:4359–4365. https://doi.org/10.1046/j.1432-1327.2001.02358.x.

Kawai, S., Suzuki, S., Mori, S., and Murata, K. (2001b). Molecular cloning and identification of *UTR1* of a yeast *Saccharomyces cerevisiae* as a gene encoding an NAD kinase. FEMS Microbiol **200**:181–184. https://doi.org/10.1111/j.1574-6968.2001.tb10712.x.

Kelner, M.J., and Montoya, M.A. (2000). Structural organization of the human glutathione reductase gene: Determination of correct cDNA sequence and identification of a mitochondrial leader sequence. Biochem. Biophys. Res. Commun. **269**:366–368. https://doi.org/10.1006/bbrc.2000.2267.

Kim, Y.I., Nam, I.K., Lee, D.K., Bhandari, S., Charton, L., Kwak, S., Lim, J.Y., Hong, K., Kim, S.J., Lee, J.N., et al. (2020). Slc25a17 acts as a peroxisomal coenzyme A transporter and regulates multiorgan development in zebrafish. J. Cell. Physiol. **235**:151–165. https://doi.org/10.1002/jcp.28954.

Kojima, S., Konishi, N., Beier, M.P., Ishiyama, K., Maru, I., Hayakawa, T., and Yamaya, T. (2014). NADH-dependent glutamate synthase participated in ammonium assimilation in Arabidopsis root. Plant Signal. Behav. **9**:e29402. https://doi.org/10.4161/psb.29402.

Konig, A.C., Hartl, M., Pham, P.A., Laxa, M., Boersema, P.J., Orwat, A., Kalitventseva, I., Plochinger, M., Braun, H.P., Leister, D., et al. (2014). The Arabidopsis class II sirtuin is a lysine deacetylase and interacts with mitochondrial energy metabolism. Plant Physiol **164**:1401–1414. https://doi.org/10.1104/pp.113.232496.

Kropotov, A., Kulikova, V., Nerinovski, K., Yakimov, A., Svetlova, M., Solovjeva, L., Sudnitsyna, J., Migaud, M.E., Khodorkovskiy, M., Ziegler, M., and Nikiforov, A. (2021). Equilibrative Nucleoside Transporters Mediate the Import of Nicotinamide Riboside and

Nicotinic Acid Riboside into Human Cells. Int. J. Mol. Sci. **22**:1391. https://doi.org/10.3390/ijms22031391.

Kulikova, V.A., and Nikiforov, A.A. (2020). Role of NUDIX Hydrolases in NAD and ADP-Ribose Metabolism in Mammals. Biochemistry. **85**:883–894. https://doi.org/10.1134/S0006297920080040.

Kunze, M., and Hartig, A. (2013). Permeability of the peroxisomal membrane: Lessons from the glyoxylate cycle. Front. Physiol. **4**:204. https://doi.org/10.3389/fphys.2013.00204.

Kurnasov, O., Goral, V., Colabroy, K., Gerdes, S., Anantha, S., Osterman, A., and Begley, T.P. (2003). NAD biosynthesis: Identification of the tryptophan to quinolinate pathway in bacteria. Chem. Biol. **10**:1195–1204. https://doi.org/10.1016/j.chembiol.2003.11.011.

Lapuente-Brun, E., Moreno-Loshuertos, R., Acín-Pérez, R., Latorre-Pellicer, A., Colás, C., Balsa, E., Perales-Clemente, E., Quirós, p.m., Calvo, E., Rodríguez-Hernández, M.A., et al. (2013). Supercomplex assembly determines electron flux in the mitochondrial electron transport chain. Science **340**:1567–1570. https://doi.org/10.1126/science.1230381.

Larsen, M., Holm, R., Jensen, K.G., Brodin, B., and Nielsen, C.U. (2009). Intestinal gaboxadol absorption via PAT1 (SLC36A1): modified absorption in vivo following co-administration of L-tryptophan. Br. J. Pharmacol. **157**:1380–1389. https://doi.org/10.1111/j.1476-5381.2009.00253.x.

Last, E.R.R.a.R.L. (1995). Tryptophan biosynthesis and molecular genetics. Plant Cell **7**:921–934. https://doi.org/10.1105/tpc.7.7.921.

Lemaitre, T., and Hodges, M. (2006). Expression analysis of *Arabidopsis thaliana* NAD-dependent isocitrate dehydrogenase genes shows the presence of a functional subunit that is mainly expressed in the pollen and absent from vegetative organs. Plant Cell Physiol. **47**:634–643. https://doi.org/10.1093/pcp/pcj030.

Lenaz, G. (2001). The mitochondrial production of reactive oxygen species: Mechanisms and implications in human pathology. IUBMB Life **52**:159–164. https://doi.org/10.1080/15216540152845957.

Leterrier, M., Barroso, J.B., Valderrama, R., Begara-Morales, J.C., Sánchez-Calvo, B., Chaki, M., Luque, F., Viñegla, B., Palma, J.M., and Corpas, F.J. (2016). Peroxisomal NADP-isocitrate dehydrogenase is required for Arabidopsis stomatal movement. Protoplasma **253**:403–415. https://doi.org/10.1007/s00709-015-0819-0.

Li, B.B., Wang, X., Tai, L., Ma, T.T., Shalmani, A., Liu, W.T., Li, W.Q., and Chen, K.M. (2018). NAD kinases: Metabolic targets controlling redox co-enzymes and reducing power partitioning in plant stress and development. Front. Plant Sci. **9**:379. https://doi.org/10.3389/fpls.2018.00379.

Li, W.Y., Wang, X., Li, R., Li, W.Q., and Chen, K.M. (2014). Genome-wide analysis of the *NADK* gene family in plants. PLoS One **9**:e101051. https://doi.org/10.1371/journal.pone.0101051.

Liang, C., Zhang, Y., Cheng, S., Osorio, S., Sun, Y., Fernie, A.R., Cheung, C.Y.M., and Lim, B.L. (2015). Impacts of high ATP supply from chloroplasts and mitochondria on the leaf metabolism of *Arabidopsis thaliana*. Front. Plant Sci. **6**:922. https://doi.org/10.3389/fpls.2015.00922.

Lim, S.L., Voon, C.P., Guan, X., Yang, Y., Gardeström, P., and Lim, B.L. (2020). In planta study of photosynthesis and photorespiration using NADPH and NADH/NAD$^+$ fluorescent protein sensors. Nat. Commun. **11**:3238. https://doi.org/10.1038/s41467-020-17056-0.

Lima, W.C., Varani, a.m., and Menck, C.F.M. (2009). NAD biosynthesis evolution in bacteria: Lateral gene transfer of kynurenine pathway in Xanthomonadales and Flavobacteriales. Mol. Biol. Evol. **26**:399–406. https://doi.org/10.1093/molbev/msn261.

Liu, J., Lim, S.L., Zhong, J.Y., and Lim, B.L. (2022). Bioenergetics of pollen tube growth in Arabidopsis thaliana revealed by ratiometric genetically encoded biosensors. Nat. Commun. **13**:7822. https://doi.org/10.1038/s41467-022-35486-w.

Liu, L., Su, X., Quinn, W.J., Hui, S., Krukenberg, K., Frederick, D.W., Redpath, P., Zhan, L., Chellappa, K., White, E., et al. (2018). Quantitative analysis of NAD Synthesis-breakdown fluxes. Cell Metab. **27**:1067–1080.e5. https://doi.org/10.1016/j.cmet.2018.03.018.

Liu, X., Wei, W., Zhu, W., Su, L., Xiong, Z., Zhou, M., Zheng, Y., and Zhou, D.X. (2017). Histone deacetylase AtSRT1 links metabolic flux and stress response in Arabidopsis. Mol. Plant **10**:1510–1522. https://doi.org/10.1016/j.molp.2017.10.010.

Luo, L., He, Y., Zhao, Y., Xu, Q., Wu, J., Ma, H., Guo, H., Bai, L., Zuo, J., Zhou, J.M., et al. (2019). Regulation of mitochondrial NAD pool via NAD$^+$ transporter 2 is essential for matrix NADH homeostasis and ROS production in Arabidopsis. Sci. China Life Sci. **62**:991–1002. https://doi.org/10.1007/s11427-019-9563-y.

Luongo, T.S., Eller, J.M., Lu, M.J., Niere, M., Raith, F., Perry, C., Bornstein, M.R., Oliphint, P., Wang, L., McReynolds, M.R., et al. (2020). SLC25A51 is a mammalian mitochondrial NAD$^+$ transporter. Nature **588**:174–179. https://doi.org/10.1038/s41586-020-2741-7.

Lyu, M.J.A., Du, H., Yao, H., Zhang, Z., Chen, G., Huang, Y., Ni, X., Chen, F., Zhao, Y.Y., Tang, Q., et al. (2025). A dominant role of transcriptional regulation during the evolution of C(4) photosynthesis in Flaveria species. Nat. Commun. **16**:1643. https://doi.org/10.1038/s41467-025-56901-y.

Magni, G., Orsomando, G.R.,N., and Ruggieri, S. (2008). Enzymology of mammalian NAD metabolism in health and disease. Front. Biosci. **13**:6135–6154. https://doi.org/10.2741/3143.

Maier, A., Zell, M.B., and Maurino, V.G. (2011). Malate decarboxylases: evolution and roles of NAD(P)-ME isoforms in species performing C(4) and C(3) photosynthesis. J. Exp. Bot. **62**:3061–3069. https://doi.org/10.1093/jxb/err024.

Marri, L., Zaffagnini, M., Collin, V., Issakidis-Bourguet, E., Lemaire, S. D., Pupillo, P., Sparla, F., Miginiac-Maslow, M., and Trost, P. (2009). Prompt and easy activation by specific thioredoxins of calvin cycle enzymes of *Arabidopsis thaliana* associated in the GAPDH/CP12/PRK supramolecular complex. Mol. Plant **2**:259–269. https://doi.org/10.1093/mp/ssn061.

Di Martino, C., and Pallotta, M.L. (2011). Mitochondria-localized NAD biosynthesis by nicotinamide mononucleotide adenylyltransferase in Jerusalem artichoke (*Helianthus tuberosus* L.) heterotrophic tissues. Planta **234**:657–670. https://doi.org/10.1007/s00425-011-1428-6.

Marty, L., Bausewein, D., Müller, C., Bangash, S.A.K., Moseler, A., Schwarzländer, M., Müller-Schüssele, S.J., Zechmann, B., Riondet, C., Balk, J., et al. (2019). Arabidopsis glutathione reductase 2 is indispensable in plastids, while mitochondrial glutathione is safeguarded by additional reduction and transport systems. New Phytol. **224**:1569–1584. https://doi.org/10.1111/nph.16086.

Mathialagan, S., Bi, Y.A., Costales, C., Kalgutkar, A.S., Rodrigues, A. D., and Varma, M.V.S. (2020). Nicotinic acid transport into human liver involves organic anion transporter 2 (SLC22A7). Biochem. Pharmacol. **174**:113829. https://doi.org/10.1016/j.bcp.2020.113829.

Matsui, A., and Ashihara, H. (2008). Nicotinate riboside salvage in plants: Presence of nicotinate riboside kinase in mungbean seedlings. Plant Physiol. Biochem. **46**:104–108. https://doi.org/10.1016/j.plaphy.2007.10.008.

McCue, W.M., and Finzel, B.C. (2022). Structural characterization of the human cytosolic malate dehydrogenase I. ACS Omega **7**:207–214. https://doi.org/10.1021/acsomega.1c04385.

McDonald, A.E., Vanlerberghe, G.C., and Staples, J.F. (2009). Alternative oxidase in animals: unique characteristics and taxonomic distribution. J. Exp. Biol. **212**:2627–2634. https://doi.org/10.1242/jeb.032151.

McGonigle, B., and Nelson, T. (1995). C4 Isoform of NADP-Malate Dehydrogenase. Plant Physiol. **108**:1119–1126. https://doi.org/10.1104/pp.108.3.1119.

Mehmel, M., Jovanović, N., and Spitz, U. (2020). Nicotinamide riboside-The current state of research and therapeutic uses. Nutrients **12**:1616. https://doi.org/10.3390/nu12061616.

Metzler, M.C., Rothermel, B.A., and Nelson, T. (1989). Maize NADP-malate dehydrogenase: cDNA cloning, sequence, and mRNA characterization. Plant Mol. Biol. **12**:713–722. https://doi.org/10.1007/BF00044162.

Meyer, T., Hölscher, C., Schwöppe, C., and von Schaewen, A. (2011). Alternative targeting of Arabidopsis plastidic glucose-6-phosphate dehydrogenase G6PD1 involves cysteine-dependent interaction with G6PD4 in the cytosol. Plant J. **66**:745–758. https://doi.org/10.1111/j.1365-313X.2011.04535.x.

Mhamdi, A., Mauve, C., Gouia, H., Saindrenan, P., Hodges, M., and Noctor, G. (2010). Cytosolic NADP-dependent isocitrate dehydrogenase contributes to redox homeostasis and the regulation of pathogen responses in Arabidopsis leaves. Plant Cell Environ. **33**:1112–1123. https://doi.org/10.1111/j.1365-3040.2010.02133.x.

Michishita, E., Park, J.Y., Burneskis, J.M., Barrett, J.C., and Horikawa, I. (2005). Evolutionarily conserved and nonconserved cellular localizations and functions of human SIRT proteins. Mol. Biol. Cell **16**:4623–4635. https://doi.org/10.1091/mbc.e05-01-0033.

Misevičienė, L., Krikštopaitis, K., and Čėnas, N. (2022). The comparative study of redox properties of recombinant human cytosolic and mitochondrial NADPH: Thioredoxin reductases. Chemija **33**:40–45. https://doi.org/10.6001/chemija.v33i2.4707.

Moller, I.M. (2001). Plant mitochondria and oxidative stress. Electron transport, NADPH turnover and metabolism of reactive oxygen species. Annu. Rev. Plant Physiol. Plant Mol. Biol. **52**:561–591. https://doi.org/10.1146/annurev.arplant.52.1.561.

Moller, I., and Rasmusson, A. (1998). The role of NADP in the mitochondrial matrix. Trends Plant Sci. **3**:21–27. https://doi.org/10.1016/S1360-1385(97)01156-4.

Moller, I.M., Rasmusson, A.G., and Van Aken, O. (2021). Plant mitochondria - past, present and future. Plant J. **108**:912–959. https://doi.org/10.1111/tpj.15495.

Moller, I.M., Igamberdiev, A.U., Bykova, N.V., Finkemeier, I., Rasmusson, A.G., and Schwarzlander, M. (2020). Matrix redox physiology governs the regulation of plant mitochondrial metabolism through posttranslational protein modifications. Plant Cell **32**:573–594. https://doi.org/10.1105/tpc.19.00535.

Morigasaki, S., Takata, K., Sanada, Y., Wada, K., Yee, B.C., Shin, S., and Buchanan, B.B. (1990). Novel forms of ferredoxin and ferredoxin-NADP reductase from spinach roots. Arch. Biochem. Biophys. **283**:75–80. https://doi.org/10.1016/0003-9861(90)90614-5.

Mulo, P. (2011). Chloroplast-targeted ferredoxin-NADP$^+$ oxidoreductase (FNR): Structure, function and location. Biochim. Biophys. Acta **1807**:927–934. https://doi.org/10.1016/j.bbabio.2010.10.001.

Navrot, N., Rouhier, N., Gelhaye, E., and Jacquot, J.P. (2006). Reactive oxygen species generation and antioxidant systems in plant mitochondria. Physiol. Plantarum **129**:185–195. https://doi.org/10.1111/j.1399-3054.2006.00777.x.

Nazari, B., Jaquet, V., and Krause, K.H. (2023). NOX family NADPH oxidases in mammals: Evolutionary conservation and isoform-defining sequences. Redox Biol. **66**:102851. https://doi.org/10.1016/j.redox.2023.102851.

Ndrepepa, G. (2021). Aspartate aminotransferase and cardiovascular disease-a narrative review. J. Lab. Precis. Med. **6**:6. https://doi.org/10.21037/jlpm-20-93.

Nemeria, N.S., Ambrus, A., Patel, H., Gerfen, G., Adam-Vizi, V., Tretter, L., Zhou, J., Wang, J., and Jordan, F. (2014). Human 2-oxoglutarate dehydrogenase complex E1 component forms a thiamin-derived radical by aerobic oxidation of the enamine intermediate. J. Biol. Chem. **289**:29859–29873. https://doi.org/10.1074/jbc.M114.591073.

Noctor, G., Veljovic-Jovanovic, S., and Foyer, C.H. (2000). Peroxide processing in photosynthesis: antioxidant coupling and redox signalling. Philos. Trans. R. Soc. Lond. B Biol. Sci. **355**:1465–1475. https://doi.org/10.1098/rstb.2000.0707.

Noctor, G., Hager, J., and Li, S. (2011). Biosynthesis of NAD and Its Manipulation in Plants. Adv. Bot. Res. **B1**:153–201. https://doi.org/10.1016/b978-0-12-386479-6.00002-0.

Noctor, G., Mhamdi, A., Chaouch, S., Han, Y., Neukermans, J., Marquez-Garcia, B., Queval, G., and Foyer, C.H. (2012). Glutathione in plants: an integrated overview. Plant Cell Environ. **35**:454–484. https://doi.org/10.1111/j.1365-3040.2011.02400.x.

Ocheretina, O., Haferkamp, I., Tellioglu, H., and Scheibe, R. (2000). Light-modulated NADP-malate dehydrogenases from mossfern and green algae: insights into evolution of the enzyme's regulation. Gene **258**:147–154. https://doi.org/10.1016/s0378-1119(00)00409-1.

Offermann, S., Okita, T.W., and Edwards, G.E. (2011). Resolving the compartmentation and function of C4 photosynthesis in the single-cell C4 species Bienertia sinuspersici. Plant Physiol. **155**:1612–1628. https://doi.org/10.1104/pp.110.170381.

Offermann, S., Friso, G., Doroshenk, K.A., Sun, Q., Sharpe, R.M., Okita, T.W., Wimmer, D., Edwards, G.E., and van Wijk, K.J. (2015). Developmental and Subcellular Organization of Single-Cell C(4) Photosynthesis in Bienertia sinuspersici Determined by Large-Scale Proteomics and cDNA Assembly from 454 DNA Sequencing. J. Proteome Res. **14**:2090–2108. https://doi.org/10.1021/pr5011907.

Ohashi, K., Kawai, S., and Murata, K. (2012). Identification and characterization of a human mitochondrial NAD kinase. Nat. Commun. **3**:1248. https://doi.org/10.1038/ncomms2262.

Oikawa, K., Hayashi, M., Hayashi, Y., and Nishimura, M. (2019). Re-evaluation of physical interaction between plant peroxisomes and other organelles using live-cell imaging techniques. J. Integr. Plant Biol. **61**:836–852. https://doi.org/10.1111/jipb.12805.

Oka, S.I., Hsu, C.P., and Sadoshima, J. (2012). Regulation of cell survival and death by pyridine nucleotides. Circ. Res. **111**:611–627. https://doi.org/10.1161/CIRCRESAHA.111.247932.

Oka, S.I., Titus, A.S., Zablocki, D., and Sadoshima, J. (2023). Molecular properties and regulation of NAD$^+$ kinase (NADK). Redox Biol. **59**:102561. https://doi.org/10.1016/j.redox.2022.102561.

Pai, Y.J., Leung, K.Y., Savery, D., Hutchin, T., Prunty, H., Heales, S., Brosnan, M.E., Brosnan, J.T., Copp, A.J., and Greene, N.D.E. (2015). Glycine decarboxylase deficiency causes neural tube defects and features of non-ketotic hyperglycinemia in mice. Nat. Commun. **6**:6388. https://doi.org/10.1038/ncomms7388.

Palmieri, F., Rieder, B., Ventrella, A., Blanco, E., Do, P.T., Nunes-Nesi, A., Trauth, A.U., Fiermonte, G., Tjaden, J., Agrimi, G., et al. (2009). Molecular identification and functional characterization of *Arabidopsis thaliana* mitochondrial and chloroplastic NAD$^+$ carrier proteins. J. Biol. Chem. **284**:31249–31259. https://doi.org/10.1074/jbc.M109.041830.

Patel, M.S., Nemeria, N.S., Furey, W., and Jordan, F. (2014). The pyruvate dehydrogenase complexes: Structure-based function and regulation. J. Biol. Chem. **289**:16615–16623. https://doi.org/10.1074/jbc.R114.563148.

Petersen, J., Brinkmann, H., and Cerff, R. (2003). Origin, evolution, and metabolic role of a novel glycolytic GAPDH enzyme recruited by land plant plastids. J. Mol. Evol. **57**:16–26. https://doi.org/10.1007/s00239-002-2441-y.

Plaitakis, A., Kalef-Ezra, E., Kotzamani, D., Zaganas, I., and Spanaki, C. (2017). The glutamate dehydrogenase pathway and its roles in cell and tissue biology in health and disease. Biology **6**:11. https://doi.org/10.3390/biology6010011.

Poirier, Y., Antonenkov, V.D., Glumoff, T., and Hiltunen, J.K. (2006). Peroxisomal beta-oxidation-a metabolic pathway with multiple functions. Biochim. Biophys. Acta **1763**:1413–1426. https://doi.org/10.1016/j.bbamcr.2006.08.034.

Pollak, N., Niere, M., and Ziegler, M. (2007). NAD kinase levels control the NADPH concentration in human cells. J. Biol. Chem. **282**:33562–33571. https://doi.org/10.1074/jbc.M704442200.

Pracharoenwattana, I., Cornah, J.E., and Smith, S.M. (2005). Arabidopsis peroxisomal citrate synthase is required for fatty acid respiration and seed germination. Plant Cell **17**:2037–2048. https://doi.org/10.1105/tpc.105.031856.

Preiss, J., and Handler, P. (1958). Biosynthesis of diphosphopyridine nucleotide. J. Biol. Chem. **233**:493–500. https://doi.org/10.1016/s0021-9258(18)64789-1.

Qiu, X.M., Sun, Y.Y., Ye, X.Y., and Li, Z.G. (2019). Signaling role of glutamate in plants. Front. Plant Sci. **10**:1743. https://doi.org/10.3389/fpls.2019.01743.

Rao, X., and Dixon, R.A. (2016). The Differences between NAD-ME and NADP-ME Subtypes of C_4 Photosynthesis: More than Decarboxylating Enzymes. Front. Plant Sci. **7**:1525. https://doi.org/10.3389/fpls.2016.01525.

Rasmusson, A.G., and Møller, I.M. (1990). NADP-Utilizing enzymes in the matrix of plant mitochondria. Plant Physiol. **94**:1012–1018. https://doi.org/10.1104/pp.94.3.1012.

Rasmusson, A.G., and Møller, I.M. (1991). Effect of calcium ions and inhibitors on internal NAD(P)H dehydrogenases in plant mitochondria. Eur. J. Biochem. **202**:617–623. https://doi.org/10.1111/j.1432-1033.1991.tb16415.x.

Rasmusson, A.G., Geisler, D.A., and Møller, I.M. (2008). The multiplicity of dehydrogenases in the electron transport chain of plant mitochondria. Mitochondrion **8**:47–60. https://doi.org/10.1016/j.mito.2007.10.004.

Rasmusson, A.G., Escobar, M.A., Hao, M., Podgórska, A., and Szal, B. (2020). Mitochondrial NAD(P)H oxidation pathways and nitrate/ammonium redox balancing in plants. Mitochondrion **53**:158–165. https://doi.org/10.1016/j.mito.2020.05.010.

Reidl, J., Schlör, S., Kraiss, A., Schmidt-Brauns, J., Kemmer, G., and Soleva, E. (2000). NADP and NAD utilization in *Haemophilus influenzae*. Mol. Microbiol. **35**:1573–1581. https://doi.org/10.1046/j.1365-2958.2000.01829.x.

Reitman, Z.J., and Yan, H. (2010). Isocitrate dehydrogenase 1 and 2 mutations in cancer: Alterations at a crossroads of cellular metabolism. J. Natl. Cancer Inst. **102**:932–941. https://doi.org/10.1093/jnci/djq187.

Riens, B., and Heldt, H.W. (1992). Decrease of nitrate reductase activity in spinach. Plant Physiol. **98**:573–577. https://doi.org/10.1104/pp.98.2.573.

Rodionov, D.A., De Ingeniis, J., Mancini, C., Cimadamore, F., Zhang, H., Osterman, A.L., and Raffaelli, N. (2008). Transcriptional regulation of NAD metabolism in bacteria: NrtR family of Nudix-related regulators. Nucleic Acids Res. **36**:2047–2059. https://doi.org/10.1093/nar/gkn047.

Rokka, A., Antonenkov, V.D., Soininen, R., Immonen, H.L., Pirilä, P.L., Bergmann, U., Sormunen, R.T., Weckström, M., Benz, R., and Hiltunen, J.K. (2009). Pxmp2 is a channel-forming protein in mammalian peroxisomal membrane. PLoS One **4**:e5090. https://doi.org/10.1371/journal.pone.0005090.

Ruiz, J.M., Sánchez, E., García, P.C., López-Lefebre, L.R., Rivero, R. M., and Romero, L. (2002). Proline metabolism and NAD kinase activity in green bean plants subjected to cold-shock. Phytochemistry **59**:473–478. https://doi.org/10.1016/S0031-9422(01)00481-2.

Sage, R.F. (2016). A portrait of the C4 photosynthetic family on the 50th anniversary of its discovery: species number, evolutionary lineages, and Hall of Fame. J. Exp. Bot. **67**:4039–4056. https://doi.org/10.1093/jxb/erw156.

Salido, E., Pey, A.L., Rodriguez, R., and Lorenzo, V. (2012). Primary hyperoxalurias: Disorders of glyoxylate detoxification. Biochim. Biophys. Acta **1822**:1453–1464. https://doi.org/10.1016/j.bbadis.2012.03.004.

Sanchez, J.P., Duque, P., and Chua, N.H. (2004). ABA activates ADPR cyclase and cADPR induces a subset of ABA-responsive genes in Arabidopsis. Plant J. **38**:381–395. https://doi.org/10.1111/j.1365-313X.2004.02055.x.

Sazanov, L.A. (2014). The mechanism of coupling between electron transfer and proton translocation in respiratory complex I. J. Bioenerg. Biomembr. **46**:247–253. https://doi.org/10.1007/s10863-014-9554-z.

Schertl, P., and Braun, H.P. (2014). Respiratory electron transfer pathways in plant mitochondria. Front. Plant Sci. **5**:163. https://doi.org/10.3389/fpls.2014.00163.

Schueren, F., Lingner, T., George, R., Hofhuis, J., Dickel, C., Gärtner, J., and Thoms, S. (2014). Peroxisomal lactate dehydrogenase is generated by translational readthrough in mammals. Elife **3**:e03640. https://doi.org/10.7554/eLife.03640.

Schulze, S., Westhoff, P., and Gowik, U. (2016). Glycine decarboxylase in C3, C4 and C3-C4 intermediate species. Curr. Opin. Plant Biol. **31**:29–35. https://doi.org/10.1016/j.pbi.2016.03.011.

Selinski, J., and Scheibe, R. (2019). Malate valves: Old shuttles with new perspectives. Plant Biol. **21**:21–30. https://doi.org/10.1111/plb.12869.

Shen, B.R., Wang, L.M., Lin, X.L., Yao, Z., Xu, H.W., Zhu, C.H., Teng, H. Y., Cui, L.L., Liu, E.E., Zhang, J.J., et al. (2019). Engineering a New Chloroplastic Photorespiratory Bypass to Increase Photosynthetic Efficiency and Productivity in Rice. Mol. Plant **12**:199–214. https://doi.org/10.1016/j.molp.2018.11.013.

Shen, J., Zeng, Y., Zhuang, X., Sun, L., Yao, X., Pimpl, P., and Jiang, L. (2013). Organelle pH in the Arabidopsis endomembrane system. Mol. Plant **6**:1419–1437. https://doi.org/10.1093/mp/sst079.

Simpson, J.P., Di Leo, R., Dhanoa, P.K., Allan, W.L., Makhmoudova, A., Clark, S.M., Hoover, G.J., Mullen, R.T., and Shelp, B.J. (2008). Identification and characterization of a plastid-localized Arabidopsis glyoxylate reductase isoform: comparison with a cytosolic isoform and implications for cellular redox homeostasis and aldehyde detoxification. J. Exp. Bot. **59**:2545–2554. https://doi.org/10.1093/jxb/ern123.

Smith, E.N., Schwarzländer, M., Ratcliffe, R.G., and Kruger, N.J. (2021). Shining a light on NAD- and NADP-based metabolism in plants. Trends Plant Sci. **26**:1072–1086. https://doi.org/10.1016/j.tplants.2021.06.010.

Sommer, M., Bräutigam, A., and Weber, A.p.m. (2012). The dicotyledonous NAD malic enzyme C4 plant Cleome gynandra displays age-dependent plasticity of C4 decarboxylation biochemistry. Plant Biol. **14**:621–629. https://doi.org/10.1111/j.1438-8677.2011.00539.x.

Song, J., Keppler, B.D., Wise, R.R., and Bent, A.F. (2015). PARP2 is the predominant poly(ADP-Ribose) polymerase in Arabidopsis DNA damage and immune responses. PLoS Genet. **11**:e1005200. https://doi.org/10.1371/journal.pgen.1005200.

Spaans, S.K., Weusthuis, R.A., van der Oost, J., and Kengen, S.W.M. (2015). NADPH-generating systems in bacteria and archaea. Front. Microbiol. **6**:742. https://doi.org/10.3389/fmicb.2015.00742.

Sun, F., Suen, P.K., Zhang, Y., Liang, C., Carrie, C., Whelan, J., Ward, J.L., Hawkins, N.D., Jiang, L., and Lim, B.L. (2012). A dual-targeted purple acid phosphatase in Arabidopsis thaliana moderates carbon metabolism and its overexpression leads to faster plant growth and higher seed yield. New Phytol. **194**:206–219. https://doi.org/10.1111/j.1469-8137.2011.04026.x.

Sweetlove, L.J., Beard, K.F.M., Nunes-Nesi, A., Fernie, A.R., and Ratcliffe, R.G. (2010). Not just a circle: Flux modes in the plant TCA cycle. Trends Plant Sci. **15**:462–470. https://doi.org/10.1016/j.tplants.2010.05.006.

Sweetman, C., Waterman, C.D., Rainbird, B.M., Smith, p.m.C., Jenkins, C.D., Day, D.A., and Soole, K.L. (2019). AtNDB2 Is the Main External NADH Dehydrogenase in Mitochondria and Is Important for Tolerance to Environmental Stress. Plant Physiol. **181**:774–788. https://doi.org/10.1104/pp.19.00877.

Szal, B., Dabrowska, Z., Malmberg, G., Gardeström, P., and Rychter, a.m. (2008). Changes in energy status of leaf cells as a consequence of mitochondrial genome rearrangement. Planta **227**:697–706. https://doi.org/10.1007/s00425-007-0652-6.

Takahashi, M., Sasaki, Y., Ida, S., and Morikawa, H. (2001). Nitrite reductase gene enrichment improves assimilation of NO_2 in Arabidopsis. Plant Physiol. **126**:731–741. https://doi.org/10.1104/pp.126.2.731.

Tanaka, M., Takahashi, R., Hamada, A., Terai, Y., Ogawa, T., Sawa, Y., Ishikawa, T., and Maruta, T. (2021). Distribution and functions of monodehydroascorbate reductases in plants: Comprehensive reverse genetic analysis of *Arabidopsis thaliana* enzymes. Antioxidants **10**:1726. https://doi.org/10.3390/antiox10111726.

Tao, R., Zhao, Y., Chu, H., Wang, A., Zhu, J., Chen, X., Zou, Y., Shi, M., Liu, R., Su, N., et al. (2017). Genetically encoded fluorescent sensors reveal dynamic regulation of NADPH metabolism. Nat. Methods **14**:720–728. https://doi.org/10.1038/nmeth.4306.

Terce-Laforgue, T., Dubois, F., Ferrario-Mery, S., de Crecenzo, M.A., Sangwan, R., and Hirel, B. (2004). Glutamate dehydrogenase of tobacco is mainly induced in the cytosol of phloem companion cells when ammonia is provided either externally or released during photorespiration. Plant Physiol **136**:4308–4317. https://doi.org/10.1104/pp.104.047548.

TeSlaa, T., Ralser, M., Fan, J., and Rabinowitz, J.D. (2023). The pentose phosphate pathway in health and disease. Nat. Metab. **5**:1275–1289. https://doi.org/10.1038/s42255-023-00863-2.

Timm, S., Florian, A., Jahnke, K., Nunes-Nesi, A., Fernie, A.R., and Bauwe, H. (2011). The hydroxypyruvate-reducing system in Arabidopsis: Multiple enzymes for the same end. Plant Physiol. **155**:694–705. https://doi.org/10.1104/pp.110.166538.

Tischner, R., and Kaiser, W. (2007). Chapter 18 - Nitrate assimilation in plants. In Biology of the Nitrogen Cycle, H. Bothe, S. Ferguson, and W. Newton, eds. (Elsevier), pp. 283–301. https://doi.org/10.1016/B978-044452857-5.50019-9.

Torres, M.A., and Dangl, J.L. (2005). Functions of the respiratory burst oxidase in biotic interactions, abiotic stress and development. Curr. Opin. Plant Biol. **8**:397–403. https://doi.org/10.1016/j.pbi.2005.05.014.

Tourmente, M., Villar-Moya, P., Rial, E., and Roldan, E.R.S. (2015). Differences in ATP generation via glycolysis and oxidative phosphorylation and relationships with sperm motility in mouse species. J. Biol. Chem. **290**:20613–20626. https://doi.org/10.1074/jbc.M115.664813.

Tovar-Mendez, A., Miernyk, J.A., and Randall, D.D. (2003). Regulation of pyruvate dehydrogenase complex activity in plant cells. Eur. J. Biochem. **270**:1043–1049. https://doi.org/10.1046/j.1432-1033.2003.03469.x.

Travelli, C., Colombo, G., Mola, S., Genazzani, A.A., and Porta, C. (2018). NAMPT: A pleiotropic modulator of monocytes and macrophages. Pharmacol. Res. **135**:25–36. https://doi.org/10.1016/j.phrs.2018.06.022.

Tristan, C., Shahani, N., Sedlak, T.W., and Sawa, A. (2011). The diverse functions of GAPDH: views from different subcellular compartments. Cell. Signal. **23**:317–323. https://doi.org/10.1016/j.cellsig.2010.08.003.

Tronconi, M.A., Fahnenstich, H., Gerrard Weehler, M.C., Andreo, C.S., Flügge, U.I., Drincovich, M.F., and Maurino, V.G. (2008). Arabidopsis NAD-malic enzyme functions as a homodimer and heterodimer and has a major impact on nocturnal metabolism. Plant Physiol. **146**:1540–1552. https://doi.org/10.1104/pp.107.114975.

Trovato, M., Funck, D., Forlani, G., Okumoto, S., and Amir, R. (2021). Editorial: Amino acids in plants: Regulation and functions in development and stress defense. Front. Plant Sci. **12**:772810. https://doi.org/10.3389/fpls.2021.772810.

Van Aken, O., Zhang, B., Carrie, C., Uggalla, V., Paynter, E., Giraud, E., and Whelan, J. (2009). Defining the mitochondrial stress response in Arabidopsis thaliana. Mol. Plant **2**:1310–1324. https://doi.org/10.1093/mp/ssp053.

van Roermund, C.W.T., Schroers, M.G., Wiese, J., Facchinelli, F., Kurz, S., Wilkinson, S., Charton, L., Wanders, R.J.A., Waterham, H.R., Weber, A.p.m., and Link, N. (2016). The peroxisomal NAD carrier from Arabidopsis imports NAD in exchange with AMP. Plant Physiol. **171**:2127–2139. https://doi.org/10.1104/pp.16.00540.

Van Veldhoven, P.P. (2010). Biochemistry and genetics of inherited disorders of peroxisomal fatty acid metabolism. J. Lipid Res. **51**:2863–2895. https://doi.org/10.1194/jlr.R005959.

Van Veldhoven, P.P., de Schryver, E., Young, S.G., Zwijsen, A., Fransen, M., Espeel, M., Baes, M., and Van Ael, E. (2020). *Slc25a17* gene trapped mice: PMP34 plays a role in the peroxisomal degradation of phytanic and pristanic acid. Front. Cell Dev. Biol. **8**:144. https://doi.org/10.3389/fcell.2020.00144.

Vanacker, H., Guichard, M., Bohrer, A.S., and Issakidis-Bourguet, E. (2018). Redox regulation of monodehydroascorbate reductase by thioredoxin y in plastids revealed in the context of water stress. Antioxidants **7**:183. https://doi.org/10.3390/antiox7120183.

Vanlerberghe, G.C. (2013). Alternative oxidase: a mitochondrial respiratory pathway to maintain metabolic and signaling homeostasis during abiotic and biotic stress in plants. Int. J. Mol. Sci. **14**:6805–6847. https://doi.org/10.3390/ijms14046805.

Voon, C.P., and Lim, B.L. (2019). ATP translocation and chloroplast biology. Natl. Sci. Rev. **6**:1073–1076. https://doi.org/10.1093/nsr/nwz089.

Voon, C.P., Guan, X., Sun, Y., Sahu, A., Chan, M.N., Gardeström, P., Wagner, S., Fuchs, P., Nietzel, T., Versaw, W.K., et al. (2018). ATP compartmentation in plastids and cytosol of Arabidopsis thaliana revealed by fluorescent protein sensing. Proc. Natl. Acad. Sci. USA **115**:E10778–E10787. https://doi.org/10.1073/pnas.1711497115.

Wagner, R., Feth, F., and Wagner, K.G. (1986). The pyridine-nucleotide cycle in tobacco. Planta **167**:226–232. https://doi.org/10.1007/BF00391419.

Wakao, S., and Benning, C. (2005). Genome-wide analysis of glucose-6-phosphate dehydrogenases in Arabidopsis. Plant J. **41**:243–256. https://doi.org/10.1111/j.1365-313X.2004.02293.x.

Wang, X., Li, W.Y., Zhang, M.M., Gao, Y.T., Liu, W.T., Li, W.Q., Muhammad, I., and Chen, K.M. (2015). Identification and functional Analysis of the NADK gene family in wheat. Plant Mol. Biol. Report. **34**:118–135. https://doi.org/10.1007/s11105-015-0904-8.

Watson-Lazowski, A., Papanicolaou, A., Sharwood, R., and Ghannoum, O. (2018). Investigating the NAD-ME biochemical pathway within C(4) grasses using transcript and amino acid variation in C(4) photosynthetic genes. Photosynth. Res. **138**:233–248. https://doi.org/10.1007/s11120-018-0569-x.

Wei, Y., Lin, M., Oliver, D.J., and Schnable, P.S. (2009). The roles of aldehyde dehydrogenases (ALDHs) in the PDH bypass of Arabidopsis. BMC Biochem. **10**:7. https://doi.org/10.1186/1471-2091-10-7.

Wheeler, M.C.G., Tronconi, M.A., Drincovich, M.F., Andreo, C.S., Flügge, U.I., and Maurino, V.G. (2005). A comprehensive analysis of the NADP-malic enzyme gene family of Arabidopsis. Plant Physiol. **139**:39–51. https://doi.org/10.1104/pp.105.065953.

Wieloch, T. (2021). A cytosolic oxidation-reduction cycle in plant leaves. J. Exp. Bot. **72**:4186–4189. https://doi.org/10.1093/jxb/erab128.

Wigge, B., Krömer, S., and Gardeström, P. (1993). The redox levels and subcellular distribution of pyridine nucleotides in illuminated barley leaf protoplasts studied by rapid fractionation. Physiol. Plantarum **88**:10–18. https://doi.org/10.1111/j.1399-3054.1993.tb01754.x.

Wilson, D.F. (2017). Oxidative phosphorylation: regulation and role in cellular and tissue metabolism. J. Physiol. **595**:7023–7038. https://doi.org/10.1113/JP273839.

Xiao, W., Wang, R.S., Handy, D.E., and Loscalzo, J. (2018). NAD(H) and NADP(H) redox couples and cellular energy metabolism. Antioxid. Redox Signal. **28**:251–272. https://doi.org/10.1089/ars.2017.7216.

Xin, C.P., Tholen, D., Devloo, V., and Zhu, X.G. (2015). The benefits of photorespiratory bypasses: how can they work? Plant Physiol. **167**:574–585. https://doi.org/10.1104/pp.114.248013.

Yokochi, Y., Yoshida, K., Hahn, F., Miyagi, A., Wakabayashi, K.I., Kawai-Yamada, M., Weber, A.p.m., and Hisabori, T. (2021). Redox regulation of NADP-malate dehydrogenase is vital for land plants under fluctuating light environment. Proc. Natl. Acad. Sci. USA **118**: e2016903118. https://doi.org/10.1073/pnas.2016903118.

Yoshimura, K., and Shigeoka, S. (2015). Versatile physiological functions of the Nudix hydrolase family in Arabidopsis. Biosci. Biotechnol. Biochem. **79**:354–366. https://doi.org/10.1080/09168451.2014.987207.

Yu, L., Fan, J., and Xu, C. (2019). Peroxisomal fatty acid beta-oxidation negatively impacts plant survival under salt stress. Plant Signal. Behav. **14**:1561121. https://doi.org/10.1080/15592324.2018.1561121.

Zeng, L., Deng, R., Guo, Z., Yang, S., and Deng, X. (2016). Genome-wide identification and characterization of Glyceraldehyde-3-phosphate dehydrogenase genes family in wheat (*Triticum aestivum*). BMC Genom. **17**:240. https://doi.org/10.1186/s12864-016-2527-3.

Zhang, W., Chen, X., Sun, W., Nie, T., Quanquin, N., and Sun, Y. (2020a). *Escherichia Coli* increases its ATP concentration in weakly acidic environments principally through the glycolytic pathway. Genes **11**:991. https://doi.org/10.3390/genes11090991.

Zhang, Z., Liang, X., Lu, L., Xu, Z., Huang, J., He, H., and Peng, X. (2020b). Two glyoxylate reductase isoforms are functionally redundant but required under high photorespiration conditions in rice. BMC Plant Biol. **20**:357. https://doi.org/10.1186/s12870-020-02568-0.

Zhao, Y., Jin, J., Hu, Q., Zhou, H.M., Yi, J., Yu, Z., Xu, L., Wang, X., Yang, Y., and Loscalzo, J. (2011). Genetically encoded fluorescent sensors for intracellular NADH detection. Cell Metab. **14**:555–566. https://doi.org/10.1016/j.cmet.2011.09.004.

Zou, Y., Wang, A., Shi, M., Chen, X., Liu, R., Li, T., Zhang, C., Zhang, Z., Zhu, L., Ju, Z., et al. (2018). Analysis of redox landscapes and dynamics in living cells and in vivo using genetically encoded fluorescent sensors. Nat. Protoc. **13**:2362–2386. https://doi.org/10.1038/s41596-018-0042-5.

Molecular Plant, Volume *18*

Supplemental information

NAD(H) and NADP(H) in plants and mammals

Danying Lu, Murray Grant, and Boon Leong Lim

Supplemental Information

Supplemental Figure S1. NAD^+ biosynthetic pathways in prokaryotes.

Supplemental Table S1. NADH generation and consumption pathways in Arabidopsis and Humans.

Supplemental Table S2. NADPH generation and consumption pathways in Arabidopsis and Humans.

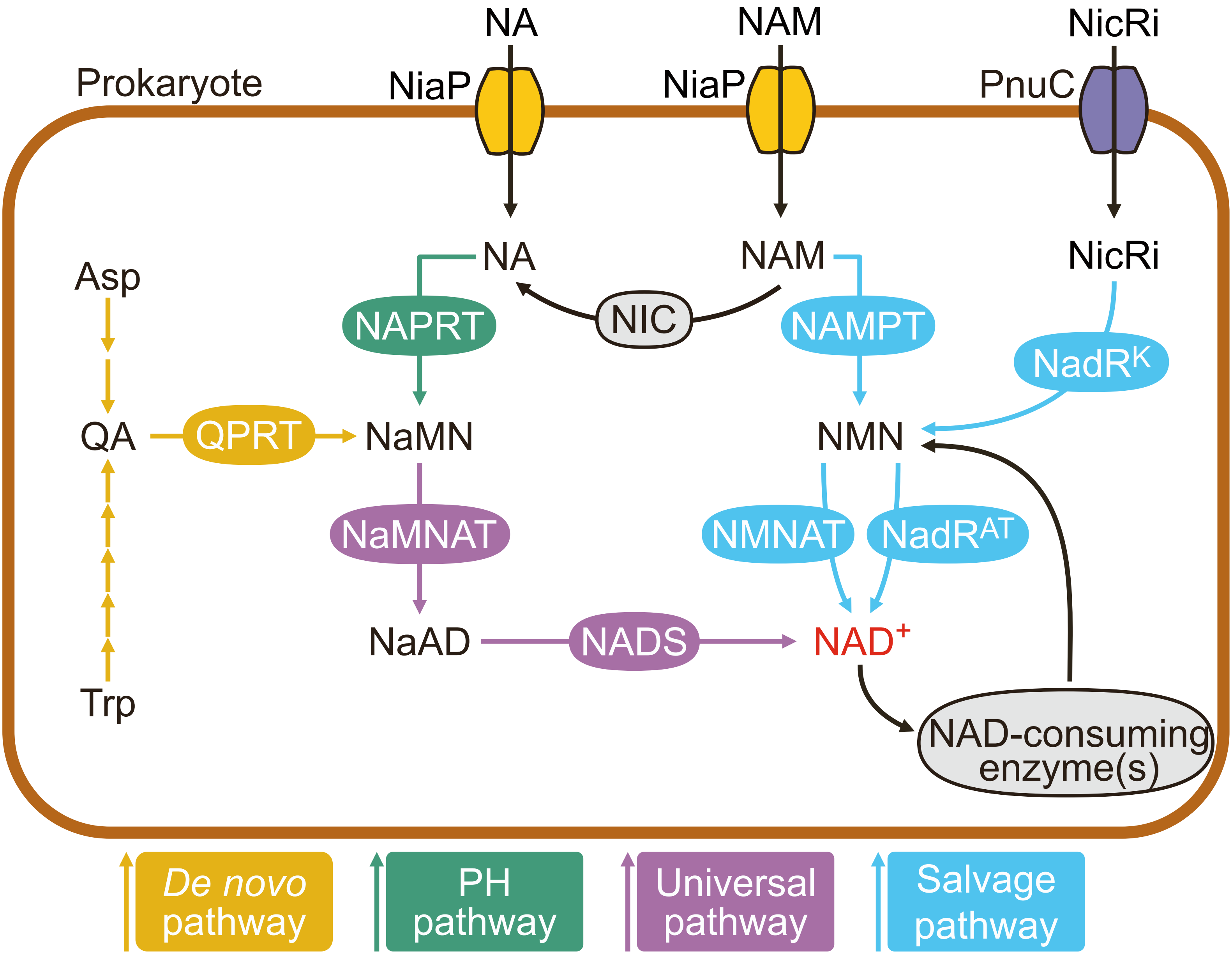

NA
NAM
NicRi
Prokaryote
NiaP
NiaP
PnuC
NA
NAM
NicRi
Asp
NAPRT
NIC
NAMPT
NadR^K
QA
QPRT
NaMN
NMN
NaMNAT
NMNAT
NadR^AT
NaAD
NADS
NAD+
Trp
NAD-consuming enzyme(s)
De novo pathway
PH pathway
Universal pathway
Salvage pathway

Supplemental Figure S1. NAD^+ biosynthetic pathways in prokaryotes.

In prokaryotes, NAD^+ can be synthesized via the *de novo* pathway, the Preiss–Handler (PH) pathway, or the salvage pathway. In the *de novo* pathway, QA is produced from Asp or Trp, which is then converted to NaMN by QPRT. NaMN is converted to NaAD and then to NAD^+ by NaMNAT and NADS, respectively. In the PH pathway, NA is converted to NaMN via NAPRT, which then enters the universal pathway to generate NAD^+. The salvage pathway uses NAM as a substrate to generate NAD^+ via NAMPT and NMNAT, or it uses NicRi as a substrate via two separate domains of NadR: $NadR^{AT}$ and $NadR^{K}$. In prokaryotes, NAM can be converted to NA via NIC. Exogenous NA and NAM are taken up by the transporter NiaP, and NicRi is taken up by the NicRi transporter PnuC.

Asp, aspartate; NA, nicotinic acid; NAM, nicotinamide; NaMN, nicotinate mononucleotide; NaAD, nicotinate adenine dinucleotide; NADS, NAD synthetase; Na/NMNAT, nicotinate/nicotinamide mononucleotide adenylyltransferase; NAPRT, nicotinate phosphoribosyltransferase; NAMPT, nicotinamide phosphoribosyl transferase; $nadR^{AT}$, nicotinamide mononucleotide adenylyltransferase; $nadR^{K}$, nicotinamide riboside kinase; niaP, niacin transporter; NIC, nicotinamidase; NMN, nicotinamide mononucleotide; NicRi, nicotinamide riboside; PnuC, NicRi uptake permease; QA, quinolinic acid; QPRT, quinolate phosphoribosyltransferase; Trp, tryptophan.

Supplemental Table S1. NADH generation and consumption pathways in Arabidopsis and Humans.

Pathway		Enzyme	Arabidopsis			Humans		
			isoform	subcellular location	reference	isoform	subcellular location	reference
**Major NADH generation pathways**	Glycolysis	glyceraldehyde 3-phosphate dehydrogenase (GAPDH)	NAD-GAPDH	cytosol	Zeng et al., 2016	GAPDH	cytosol	Ikeda et al., 2012; Tristan et al., 2011
			NAD-GAPDHp	plastid		-	-	-
	Pyruvate oxidation	pyruvate dehydrogenase complex (PDC)	mtPDC	mitochondria	Tovar-Mendez et al., 2003	PDC	mitochondria	Patel et al., 2014
			plPDC	plastid		-	-	-
	TCA cycle	isocitrate dehydrogenase (ICDH)	NAD-ICDH	mitochondria	Lemaitre and Hodges, 2006	NAD-ICDH	mitochondria	Reitman and Yan, 2010
		2-Oxoglutarate dehydrogenase complex (OGDH)	OGDH	mitochondria	Condori-Apfata et al., 2019	OGDH	mitochondria	Nemeria et al., 2014
		malate dehydrogenase	mtNAD-MDH	mitochondria	Lim et al., 2020;	mtNAD-MDH	mitochondria	McCue and Finzel,

		(MDH)	cyNAD-MDH	cytosol	Selinski and Scheibe, 2019	cpNAD-MDH	cytosol, peroxisome	2022
			plNAD-MDH	plastid		-	-	-
			pNAD-MDH	peroxisome		-	-	-
		NAD-malate enzyme (NAD-ME)	NAD-ME	mitochondria	Tronconi et al., 2008	NAD-ME	mitochondria	Hsieh et al., 2019
	Fatty acid β-oxidation	L-3-hydroxyacyl-CoA dehydrogenase (HADH)	HADH	peroxisome	Goepfert and Poirier, 2007	HADH	mitochondria, peroxisomes	Van Veldhoven, 2010
	Glyoxylate cycle	MDH	NAD-MDH	glyoxysome	De Bellis et al., 2020	-	-	-
	Glycine oxidation	Glycine decarboxylase (GDC)	GDC	mitochondria	Douce et al., 2001	GDC	mitochondria	Pai et al., 2015
	Glutamate oxidation	glutamate dehydrogenase (GDH)	NAD-GDH	mitochondria	Qiu et al., 2019	GDH	mitochondria	Bunik et al., 2016; Plaitakis et al., 2017

**Major NADH consumption pathways**	mETC	Complex I	Complex I	mitochondria	Efremov and Sazanov, 2012; Lapuente-Brun et al., 2013	Complex I	mitochondria	Efremov and Sazanov, 2012; Lapuente-Brun et al., 2013
		Alternative NADH dehydrogenase	NDA1-2	inner side of the mitochondrial inner membrane	Rasmusson et al., 2008; Rasmusson et al., 2020	-	-	-
			NDB2-4	outer side of the mitochondrial inner membrane		-	-	-
	Fermentation	lactate dehydrogenase (LDH)	LDH	cytosol	Dolferus et al., 2008	LDH	cytosol, peroxisome	Schueren et al., 2014
		acetaldehyde dehydrogenase (ADH)	ALDH	cytosol	Wei et al., 2009	-	-	-
	Nitrogen assimilation	nitrate reductase (NR)	NR	cytosol	Tischner and Kaiser, 2007	-	-	-

	GS/GOGAT cycle	GOGAT	root-GOGAT	plastid	Kojima et al., 2014	-	-	-
	Glyoxylate and hydroxypyruvate reduction	glyoxylate reductase (GR)	NAD(P)H-cyGR	cytosol	Simpson et al., 2008	glyoxylate reductase/ hydroxypyruvate reductase (GRHPR)	cytosol, mitochondria	Booth et al., 2006
			NAD(P)H-plGR	plastid				
		NAD(P)H-hydroxypyruvate reductase (HPR)	NADPH-cyHPR	cytosol	Timm et al., 2011			
			NADH-pHPR	peroxisome				
			NADPH-chlHPR	chloroplast				

Supplemental Table S2. NADPH generation and consumption pathways in Arabidopsis and Humans.

Pathway		Arabidopsis				Mammal			
		**Enzyme**	**isoform**	**subcellular location**	**reference**	**Enzyme**	**isoform**	**subcellular location**	**reference**
**Major NADPH generation pathways**	Photosyhthesis	Ferredoxin-NADP(H) oxidoreductase (FNR)	FNR	chloroplast	Morigasaki et al., 1990	-	-	-	-
	The oxidative pentose phosphate pathway (OPPP)	Glucose-6-phosphate dehydrogenase (G6PD)	cyG6PD	cytosol	Wakao and Benning, 2005	Glucose-6-phosphate dehydrogenase	G6PD	cytosol	TeSlaa et al., 2023
			plG6PD	plastid			-	-	-
		6-Phosphogluconate dehydrogenase (6PDG)	PGD	cytosol, plastid, peroxisome	Holscher et al., 2016	6-Phosphogluconate dehydrogenase	PGD	cytosol	TeSlaa et al., 2023
		Malate dehydrogenase (MDH)	NADP-MDH	chloroplast	Lim et al., 2020; Yokochi et al., 2021	-	-	-	-

		NADP-malate enzyme (NADP-ME)	cyNADP-ME	cytosol	Wheeler et al., 2005	NADP-malate enzyme (NADP-ME)	cyNADP-ME	cytosol	Ohashi et al., 2012
			plNADP-ME	plastid			mNADP-ME	mitochondria	
		Isocitrate dehydrogenase (ICDH)	cyNADP-ICDH	cytosol	Lemaitre and Hodges, 2006; Leterrier et al., 2016; Rasmusson and moller, 1990	Isocitrate dehydrogenase (ICDH)	cyNADP-ICDH	cytosol, peroxisome	Reitman and Yan, 2010
			cmNADP-ICDH	chloroplast, mitochondria			mNADP-ICDH	mitochondria	
			pNADP-ICDH	peroxisome		-	-	-	-
		Non-phosphorylating GAPDH	np-GAPDH	cytosol	Wieloch, 2021	-	-	-	-
**Major NADPH consumption pathways**	Calvin cycle	NADP-specific GAPDH	NADP-GAPDH	chloroplast	Zeng et al., 2016	-	-	-	-
	Alternative pathway of mETC	Alternative NADPH dehydrogenase	NDB1	outer side of the mitochondrial inner membrane	Rasmusson et al., 2008; Rasmusson et al., 2020	-	-	-	-
			NDC1	inner side of the mitochondrial		-	-	-	-

				inner membrane					
	Root ferredoxin reduction	Root ferredoxin	Root-FNR	plastid	Guan et al., 2018; Hachiya et al., 2016	-	-	-	-
	Nitrite reduction	nitrite reductase (NIR)	NIR	plastid	Takahashi et al., 2001	-	-	-	-
	Reactive oxygen species (ROS) production	respiratory burst oxidase homologs (RboHs)	RboH	cytosol	Kaur and Pati, 2016	NADPH oxidases (NOXs)	NOX	cytosol	Nazari et al., 2023
	Ascorbate-Glutathione (AsA-GSH) cycle	glutathione reductase (GTR)	GTR1	cytosol, nucleus and peroxisomes	Foyer and Noctor, 2011	glutathione reductase (GTR)	GTR	cytosol, mitochondria	Kelner and Montoya, 2000
			GTR2	plastids, mitochondria	Marty et al., 2019	-	-	-	-
		NAD(P)H-dependent monodehydroascorbate reductase	MDAR1	cytosol, peroxisome	Tanaka et al., 2021	-	-	-	-
			MDAR2	cytosol					
			MDAR3	cytosol					

		(MDAR)	MDAR4	peroxisomal membrane-attached					
			MDAR5	chloroplast, mitochondrial					
	The thioredoxin antioxidant system	thioredoxin reductase (TrxR)	NTRA	major NTR isoform in cytosol, also found in mitochondria and nucleus	Cha et al., 2015	thioredoxin reductase (TrxR)	TrxR1	cytosol	Misevičienė et al., 2022
			NTRB	major NTR isoform in mitochondria, also found in cytosol			TrxR2	mitochondria	
			NTRC	chloroplast					
